# Supplementary material for: Compartment and hub definitions tune metabolic networks for metabolomic interpretations
Source: Gigascience. 2020 Jan 23;9(1):giz137. doi: 10.1093/gigascience/giz137 (PMC6977586; doi:10.1093/gigascience/giz137)
Supplement: giz137_GIGA-D-18-00489_Original_Submission [file giz137_giga-d-18-00489_original_submission.pdf]

GigaScience

Non-Compartmental Metabolic Networks without Hubs Contextualize Metabolomic Measurements

--Manuscript Draft--

**Manuscript Number:**	GIGA-D-18-00489
**Full Title:**	Non-Compartmental Metabolic Networks without Hubs Contextualize Metabolomic Measurements
**Article Type:**	Research
**Funding Information:**	National Institute of Diabetes and Digestive and Kidney Diseases (1T32DK11096601) — Mr. Thomas Cameron Waller, Mr. Jordan Alexander Berg<br>National Cancer Institute (CA228346) — Dr. Jared Paul Rutter
**Abstract:**	Background: Metabolic networks represent all chemical reactions between molecular metabolites in an organism's cells. These networks provide biological context in which to integrate, analyze, and interpret "omics" measurements. While it is practical to simplify metabolic networks by multiple constraints, it is unclear how these simplifications affect the structures or relevance of these networks.<br>Results: We curated and adapted the latest systemic model of human metabolism and developed customizable tools to define metabolic networks with and without simplification for compartmentalization in subcellular organelles and exclusion of prolific metabolite hubs. Non-compartmental networks were smaller, denser, more central, and less modular than their compartmental comparators, and these networks were appropriate for metabolomic measurements that do not discriminate between compartments. Networks without hubs were smaller, less dense and central, and more modular than their comparators with hubs. When present, these hubs dominated paths in their networks, and their exclusion exposed the more subtle influences of other metabolites. In a retrospective, exploratory analysis of metabolomic measurements from studies on human tissues, network clusters identified individual reactions that might experience differential regulation in experimental conditions. Several of these reactions of interest were neither apparent in previous publications of these studies nor in our own analyses by metabolite set enrichment analysis.<br>Conclusions: Exclusion of specific metabolite hubs effectively restores modularity to the non-compartmental network that is most appropriate for metabolomic measurements, improving prospects for detection of relevant clusters. Computational detection of clusters in measurements on these networks promises to identify differential regulation of individual genes and proteins.
**Corresponding Author:**	Thomas Cameron Waller, B.S.<br>University of Utah<br>Salt Lake City, Utah UNITED STATES
**Corresponding Author Secondary Information:**	
**Corresponding Author's Institution:**	University of Utah
**Corresponding Author's Secondary Institution:**	
**First Author:**	Thomas Cameron Waller, B.S.
**First Author Secondary Information:**	
**Order of Authors:**	Thomas Cameron Waller, B.S.
	Jordan Alexander Berg, B.S.
	Brian Earl Chapman, Ph.D.
	Jared Paul Rutter, Ph.D.
**Order of Authors Secondary Information:**	

Powered by Editorial Manager® and ProduXion Manager® from Aries Systems Corporation

Additional Information:	
**Question**	**Response**
Are you submitting this manuscript to a special series or article collection?	No
**Experimental design and statistics**<br><br>Full details of the experimental design and statistical methods used should be given in the Methods section, as detailed in our Minimum Standards Reporting Checklist. Information essential to interpreting the data presented should be made available in the figure legends.<br><br>Have you included all the information requested in your manuscript?	Yes
**Resources**<br><br>A description of all resources used, including antibodies, cell lines, animals and software tools, with enough information to allow them to be uniquely identified, should be included in the Methods section. Authors are strongly encouraged to cite Research Resource Identifiers (RRIDs) for antibodies, model organisms and tools, where possible.<br><br>Have you included the information requested as detailed in our Minimum Standards Reporting Checklist?	Yes
**Availability of data and materials**<br><br>All datasets and code on which the conclusions of the paper rely must be either included in your submission or deposited in publicly available repositories (where available and ethically appropriate), referencing such data using a unique identifier in the references and in the “Availability of Data and Materials” section of your manuscript.	Yes

Powered by Editorial Manager® and ProduXion Manager® from Aries Systems Corporation

Have you have met the above requirement as detailed in our Minimum Standards Reporting Checklist?	

Powered by Editorial Manager® and ProduXion Manager® from Aries Systems Corporation

Manuscript
Click here to download Manuscript waller_gigascience_2018.pdf

OXFORD (GIGA)^n SCIENCE

GigaScience, 2017, 1–25

doi: xx.xxxx/xxxx
Manuscript in Preparation
Research

RESEARCH

Non-Compartmental Metabolic Networks without Hubs Contextualize Metabolomic Measurements

T. Cameron Waller[1, *], Jordan A. Berg[1], Brian E. Chapman[2] and Jared Rutter[1, 3, †]

[1]Department of Biochemistry, University of Utah and [2]Department of Radiology and Imaging Sciences and Department of Biomedical Informatics, University of Utah and [3]Howard Hughes Medical Institute, University of Utah

[*]Corresponding author: tcameronwaller@gmail.com
[†]Corresponding author: rutter@biochem.utah.edu

Abstract

Background: Metabolic networks represent all chemical reactions between molecular metabolites in an organism's cells. These networks provide biological context in which to integrate, analyze, and interpret "omics" measurements. While it is practical to simplify metabolic networks by multiple constraints, it is unclear how these simplifications affect the structures or relevance of these networks. **Results:** We curated and adapted the latest systemic model of human metabolism and developed customizable tools to define metabolic networks with and without simplification for compartmentalization in subcellular organelles and exclusion of prolific metabolite hubs. Non-compartmental networks were smaller, denser, more central, and less modular than their compartmental comparators, and these networks were appropriate for metabolomic measurements that do not discriminate between compartments. Networks without hubs were smaller, less dense and central, and more modular than their comparators with hubs. When present, these hubs dominated paths in their networks, and their exclusion exposed the more subtle influences of other metabolites. In a retrospective, exploratory analysis of metabolomic measurements from studies on human tissues, network clusters identified individual reactions that might experience differential regulation in experimental conditions. Several of these reactions of interest were neither apparent in previous publications of these studies nor in our own analyses by metabolite set enrichment analysis. **Conclusions:** Exclusion of specific metabolite hubs effectively restores modularity to the non-compartmental network that is most appropriate for metabolomic measurements, improving prospects for detection of relevant clusters. Computational detection of clusters in measurements on these networks promises to identify differential regulation of individual genes and proteins.

Key words: metabolism; metabolomics; network; cluster

Background

Cellular metabolism is a complex system, comprising cooperation between many types of biochemical entities. Much of the cell's structure and function rely on large, polymeric molecules–lipids, sugars, nucleic acids, and proteins. Chemical reactions between smaller molecules, metabolites, build these larger molecules and also supply energy to sustain life. Genes and transcripts encode the proteins that act as enzymes to catalyze these chemical reac-

Compiled on: December 7, 2018.
Draft manuscript prepared by the author.

Table 1. Curation of human metabolic model

**Step 1**	**Metabolites**	**Reactions**	**Compartments**	**Processes**
Count:	1725	5772	10	113
MetaNetX:	1682 (97.51%)	4354 (75.43%)		
PubChem or HMDB:	906 (52.52%)			
Gene or Enzyme:		3930 (68.09%)		
**Step 2**	**Metabolites**	**Reactions**	**Compartments**	**Processes**
Count:	1725	5772	10	113
MetaNetX:	1682 (97.51%)	4354 (75.43%)		
PubChem or HMDB:	1007 (58.38%)			
Gene or Enzyme:		3930 (68.09%)		
**Step 3**	**Metabolites**	**Reactions**	**Compartments**	**Processes**
Count:	1722	3486	8	109
MetaNetX:	1679 (97.50%)	2667 (76.51%)		
PubChem or HMDB:	1014 (58.89%)			
Gene or Enzyme:		2641 (75.76%)		

Curation of systemic model of human metabolism. The goal of curation was to adapt the model for definition of networks to represent intracellular metabolism, and to improve integration of metabolomic measurements. Step 1 was after integration of Recon 2M.2 [1] with MetaNetX [2]. Step 2 was after deriving names and references for metabolites from HMDB [3]. Step 3 was after curation of individual metabolites and reactions. Summaries comprise counts of metabolites, reactions, compartments, and processes. Summaries also comprise coverage of metabolites with references to MetaNetX [2], HMDB [3], and PubChem [4], and coverage of reactions with references to MetaNetX [2], Entrez Gene [5], and ExPASy [6, 7].

tions. Within eukaryotic cells, membrane-bound organelles compartmentalize groups of reactions to specific environments that are essential to metabolic regulation. Protein transporters regulate the exchange of metabolites between these compartments.

Metabolism is dynamic and adaptive in human health and disease, and there is a growing need for strategies to investigate its breadth. Traditional, reductionist biology conceptualizes cellular metabolism as a collection of separate pathways or groups of reactions that perform their own unique functions with limited interactions between pathways. However, a growing body of work has demonstrated surprising versatility in the metabolic system, especially in human diseases such as obesity, diabetes, and cancer [8, 9, 10]. Connectivity in metabolism is such that acute perturbations such as mutations or post-translational modifications of individual enzymes or transporters can impose pervasive effects that blur distinctions between typical pathways and cellular compartments. It is common for multiple perturbations to combine cooperatively in complex diseases [11]. Consequently, the appropriate study of metabolic mechanisms in these diseases requires experimentation at a systems level. Modern "omics" technologies measure abundance and modification of genes, transcripts, proteins, and metabolites, with nearly comprehensive coverage [12]. There is a need for strategies to integrate system-wide biological perspective in the analysis and interpretation of these measurements [13, 14].

Network representations of biological systems allow observations of their global structure and behavior, and they also offer biological context for "omics" experiments. As computer-readable, semantic models, these networks are amenable to computational searches and analyses. Interrogation of biological networks has demonstrated patterns of modularity and efficient communication, suggesting potential mechanisms of evolution and selection [15]. These analyses have also demonstrated patterns of selective connections and compartmentalization that favor robust metabolic regulation [16, 17]. As compilations of biological knowledge, these networks also offer context for the interpretation of experimental data, and there has been much work on integration and analysis of measurements from multiple "omics" technologies on biological networks [14]. Clusters or patterns of differential measurements on these networks implicate specific locations or types of biological perturbations [18], and this network strategy enhances sensitivity and specificity beyond strategies that restrict context to enrichment of disjoint sets [19]. While there has been much emphasis on networks that represent gene-gene or protein-protein interactions, metabolic networks have unique value and require specific considerations.

Custom, context-specific metabolic networks can represent different aspects of the metabolic system. Compartmentalization is an important dimension of metabolism in eukaryotic cells, yet standard metabolomic measurements do not discriminate between pools of metabolites in separate compartments. Hence, for the sake of analyzing and interpreting these measurements, it may be reasonable to simplify cellular metabolism by ignoring compartmentalization [20]. Also, a few metabolites, such as water, dioxygen, and carbon dioxide, are especially common reactants and products in metabolic reactions, and these metabolites dominate connectivity in metabolic networks as hubs [15]. Some of these hub metabolites are likely too abundant or too promiscous in the cell to drive specific metabolic regulation, and their prominence obscures more subtle metabolic perturbations. For these reasons, it is frequently practical to exclude these hub metabolites from metabolic networks [20]. While these simplifications for compartmentalization and hubs are common, it is unclear how they affect analyses of metabolic networks. Furthermore, different conditions of metabolic experiments might alter the biological relevance of specific patterns in clusters of measurements. However, there is not an available algorithm or tool to search specifically for metabolically relevant clusters.

Here we describe alternative definitions of metabolic networks and their relevance to metabolic experiments. We hypothesized

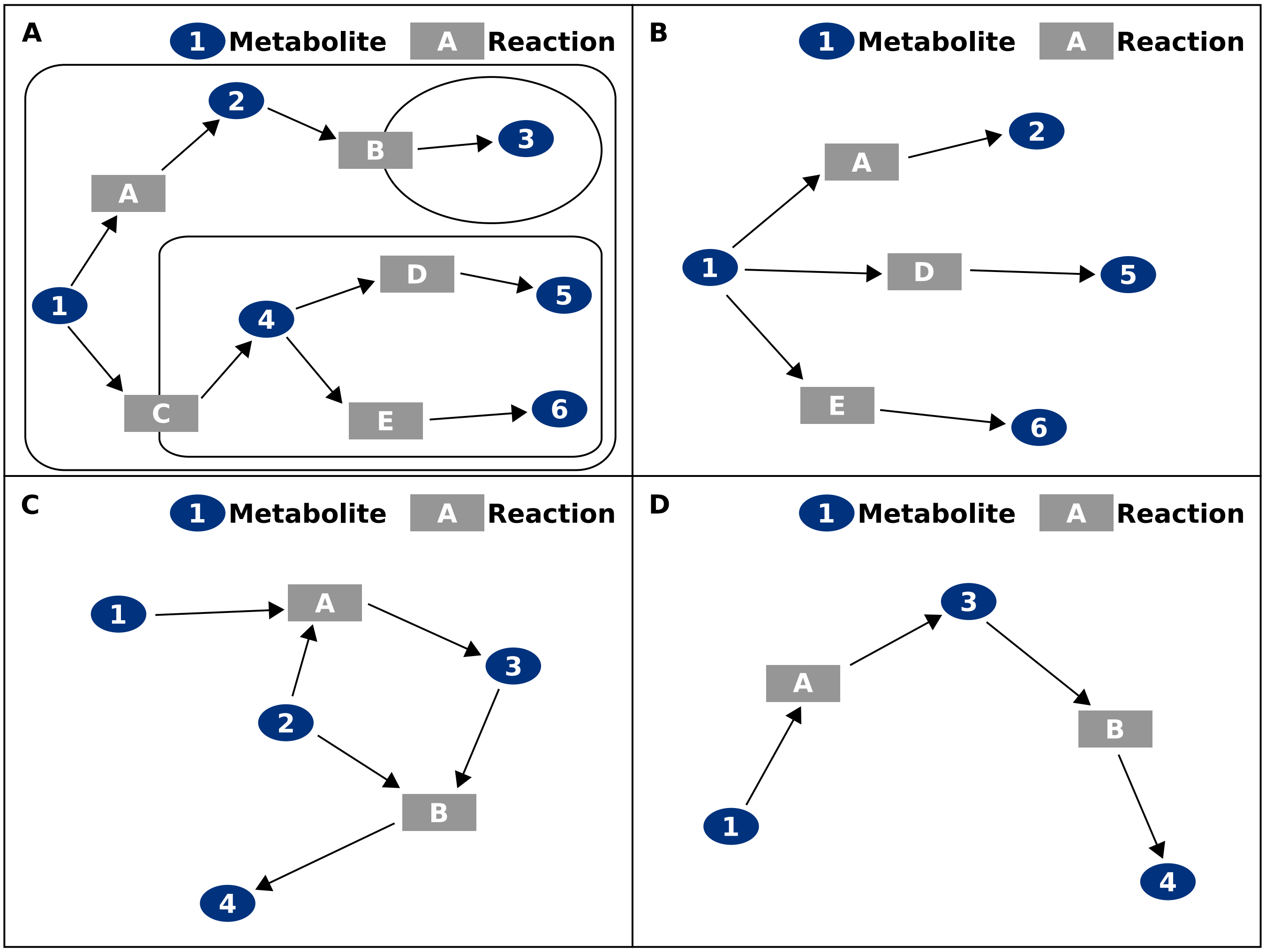

Figure 1. Definition of metabolic networks with simplifications for compartments and hubs. **A.** Compartmental network. Boxes and ellipse represent compartments. A compartmental network distinguishes between compartmental instances of otherwise chemically identical metabolites (metabolites 1 and 4, metabolites 2 and 3) and reactions. Compartmental networks also include reactions to represent transport (reactions B and C) between compartments. **B.** Non-compartmental version of network from **A**. A non-compartmental network combines chemically-identical metabolites and reactions to single consensus representations. **C.** Network with hubs. Metabolite hubs (metabolite 2) participate in many reactions and impart excessive connectivity to the network. **D.** Network from **C** without hubs. Selective exclusion of metabolite hubs simplifies the network and reveals major structural themes such as linear or cyclical pathways.

that alternative representations of metabolism in compartmental and non-compartmental networks with and without hubs would differ in their relevance to metabolomic experiments. We also explored the potential for algorithms to detect biologically relevant clusters of metabolomic measurements on these networks. Our goal was to define these networks and describe their differences, while also providing methods and tools for future use in the community. We curated and adapted the latest systemic model of human metabolism [1] for this purpose. We designed and developed a web application with a dynamic, visual interface to illustrate alternative definitions of metabolic networks. We created a software package with procedures to define these networks with customizable parameters. We analyzed these networks by various graph-theoretical metrics. Finally, we demonstrated the application of one network as biological context in a retrospective analysis of metabolomic measurements from multiple previous studies. This work informs the future development of standard tools for interpretation of "omics" measurements in metabolic experiments.

Data Description

Metabolic model

Systemic metabolic models comprise summaries of all chemical reactions between small-molecular metabolites that occur within an organism. Another term for these models is genome-scale metabolic reconstructions, and a major application is in computational simulations of broader cell growth and finer flux balance analysis [21, 22]. These models also represent concise, computer-readable, semantic indices or summaries that integrate and consolidate biological information from multiple sources [21, 22]. Often they are specific to cellular metabolism in a single species. Information about metabolites includes common names and chemical attributes such as formula, mass, and charge. Information about reactions includes common names, directionality and reversibility, metabolites that participate in the reaction and compartments where they occur. Several studies have refined information about reactions to generate tissue-specific versions

of the human model for greater specificity and accuracy [21, 22]. Importantly, both metabolites and reactions include references to external databases that offer both supporting evidence and supplemental information. Relevant references for metabolites include the Human Metabolome Database (HMDB) [3], PubChem [4], Chemical Entities of Biological Interest (ChEBI) [23], and the Kyoto Encyclopedia of Genes and Genomes (KEGG) [24]. Relevant references for reactions include KEGG, MetaCyc [25], and Reactome [26]. Also relevant to reactions are references for genes, transcripts, and proteins such as Entrez Gene [5], Human Genome Organization (HUGO) Gene Nomenclature Committee (HGNC) [27], Reference Sequence (RefSeq) [28], Ensembl [29], UniProt [30], and ExPASy Enzyme Nomenclature Database (ExPASy) [6, 7].

These models offer advantageous transparency and accessibility. They develop iteratively through the collaborative collection and curation of information from genomic annotations, comparative biology, and review of the scientific literature and databases [21, 22]. They are often open-source and publicly available with persistent version identifiers. Current models are available for multiple model organisms including *Escherichia coli* (bacterium), *Saccharomyces cerevisiae* (yeast), *Mus musculus* (mouse), and *Homo sapiens* (human) [1]. The model of human metabolism has evolved through many iterations and much effort from a broad, collaborative community [31, 32, 33, 34, 35, 1]. Repositories of common information in models for multiple species are useful for standardization, quality control, and comparison. These repositories include BiGG [36], and MetaNetX [2], and relevant tools include MetExplore [37]. Metabolic models are commonly available from repositories in an open format, which is a standard definition of Extensible Markup Language (XML) known as the Systems Biology Markup Language (SBML) [38].

Metabolomic measurements

Metabolomic technologies separate, identify, and quantify small-molecules from biological samples. While some studies utilize Nuclear Magnetic Resonance (NMR), larger studies commonly employ chromatography with gas (GC) or liquid (LC) mobile phases that integrate with various forms of mass spectrometry (MS). Combinations of measurements from multiple technologies, such as GC-MS and LC-MS, detect more analytes in samples, thereby increasing the breadth of a study. Whereas targeted studies specifically search for signals for identifiable analytes, untargeted studies tend to give much broader coverage and instead search for observable differences in analytes before their identification. Targeted studies tend to include up to 800 unique, identifiable analytes [20]. Each type of technology has its own parameters and requirements for processing, and analyzing the data. In particular, normalization to total signal in each sample corrects for loss of material and fluctuation in detector sensitivity. Furthermore, measurements commonly lack absolute calibration such that values only represent relative comparisons between samples. Many data sets from metabolomic studies are inaccessible, but there is an initiative to include more of these data in public repositories, such as the Metabolomics Workbench [39].

Analyses

Tools for definition and analysis of human metabolic networks

MetaboNet [40] is a collection of information and tools that support the majority of our analyses and observations herein. This collection includes instructions to access larger files of source information from external repositories along with internal files of customizable parameters. An installable package in the Python programming language organizes our procedures for curation of the human metabolic model, definition of customizable metabolic networks, analysis of these networks, and processing and integration of metabolomic measurements. This public collection enhances the reproducibility of our work.

Curation and adaptation of the human metabolic model

We chose to base our analyses on systemic metabolic models. As open source projects from contributions of a broad community of experts, these models offer excellent accessibility and transparency. These models are also versatile with potential to develop further.

We curated the latest model of human metabolism and adapted it to provide biological context in metabolic experiments. Step 1: We integrated Recon 2M.2 [1] into the MetaNetX [2] repository to standardize identifiers, control for quality, and include supplemental information about metabolites and reactions. Step 2: We matched metabolites to entries in HMDB [3] to standardize common names and to enhance references both to HMDB and to PubChem [4]. Step 3: We included 197 custom curations for metabolites and 102 custom curations for reactions to increase accuracy and to improve our ability to integrate metabolomic measurements. Our interest in the model was its compilation of basic knowledge about intracellular relations between metabolites and reactions. We adapted the model by removing metabolites and reactions that were primarily relevant to simulations of growth and metabolic flux, such as biomass accumulation, protein assembly and degradation, and exchange with the extracellular space or boundary of the system. This specialization simplified the model's scale substantially (**Table 1**), effectively eliminating potential noise from our subsequent analyses. In our final version of the model, 58.89% of 1722 metabolites included references to entries either in HMDB or in PubChem (**Table 1**). Similarly, 75.76% of 3486 reactions included references to entries either in Entrez Gene [5] or in ExPASy [6, 7] (**Table 1**). We consider this coverage of external references as an informative metric for the accuracy and reliability of information in this model.

Definition of metabolic networks

We chose to represent metabolism in a directional bipartite network [41] with distinct types of nodes for reactions and metabolites (**Figure 1D**). This representation is intuitive for interactions between distinct biological entities for small molecules as metabolites and the genes, transcripts, and proteins that mediate chemical reactions. These nodes store attributes appropriate to their type of biological entity. Directional links depict relations between metabolites and reactions, representing reactant and product metabolites and reaction reversibility (**Figure 1D**). We define these networks with specific constraints to emphasize different aspects of metabolism (**Figure 1**).

Table 2. Definition of metabolite hubs.

Name	Non-Compartmental Degree	Compartmental Degree
Proton	1502	1880
Water	1070	1240
Dioxygen	270	300
Phosphate	189	290
Diphosphate	126	183
Carbon dioxide	123	160
Sulfate	109	131
Hydrogen peroxide	107	132
Ammonium	68	84
Sulfite	7	24
Sodium	0	20
Hydrogen carbonate	0	0
Hydroxide	0	0

Name	Non-Compartmental Degree	Compartmental Degree
Coenzyme-A	473	593
Nicotinamide adenine dinucleotide (NAD1+)	349	421
Nicotinamide adenine dinucleotide reduced (NADH)	345	405
Nicotinamide adenine dinucleotide phosphate (NADP1+)	284	316
Nicotinamide adenine dinucleotide phosphate reduced (NADPH)	282	314
Acetyl coenzyme-A	169	215
(R)-Carnitine	149	276
Flavin adenine dinucleotide (FAD2+)	100	112
Flavin adenine dinucleotide reduced (FADH2)	98	106
Adenosine 5'-triphosphate (ATP)	245	392
Adenosine 5'-diphosphate (ADP)	167	280
Uridine 5'-diphosphate (UDP)	164	191
Adenosine 5'-monophosphate (AMP)	94	137
Cytidine 5'-monophosphate (CMP)	59	83
Adenosine 3',5'-bisphosphate (ABP)	55	57
Guanosine 5'-diphosphate (GDP)	52	75

Names and degrees of metabolite hubs for exclusion from metabolic networks. The degree of a metabolite's node is the count of links to reactions in which it participates as reactant or product. A metabolite's non-compartmental degree is the degree of its node in the non-compartmental network. A metabolite's compartmental degree is the cumulative degree across all nodes for its instances in the compartmental network. Metabolites on top are in Category 1. Metabolites on bottom are in Category 2.

Constraint 1: Compartmentalization

Our first constraint involves compartmentalization. Compartmental networks (**Figure 1A**) include compartment-specific instances of otherwise chemically identical metabolites and reactions. These networks also include reactions to represent transport between compartments. Non-compartmental networks (**Figure 1B**) abbreviate these chemically identical metabolites and reactions in single consensus representations that are each unique. These networks also exclude transport reactions as these are irrelevant without compartments.

Constraint 2: Filters by compartments and processes

Our second constraint involves filters by specific cellular compartments and metabolic processes. These compartments and processes define sets of metabolites and reactions of interest. For example the user might want to consider only reactions and metabolites within the Mitochondrion compartment. Similarly, the user might want to consider only reactions and metabolites that participate in the Citric Acid Cycle process (**Figure 2B**). MetaboNet [40] makes these filters customizable. We included metabolites and reactions from all compartments and processes in our subsequent analyses.

Constraint 3: Exclusion of specific metabolites

Our third constraint relates to the exclusion of specific metabolites from the metabolic network. This exclusion means that the network does not include nodes to represent these metabolites, and consequently there are also no links to or from them. Regardless of exclusion of nodes and links for a metabolite, reactions themselves still include information about all metabolites that participate as reactants and products.

Metabolite hubs are special candidates for exclusion from the metabolic network. A few metabolites are common reactants and products in metabolic reactions, such that they contribute a large proportion of the connectivity in metabolic networks (**Table 2**) [20]. These metabolites are hubs, and they are of special interest because they dominate the structure of metabolic networks. Exclusion of these hubs simplifies connectivity in metabolic networks (**Figure 1C-D, Figure 2A-B**) and improves chances of detecting trends in other, less dominant metabolites. We divided these hubs into 2 conceptual categories on the basis of their relevance to metabolic regulation and experiments.

Category 1 Hubs. Category 1 metabolite hubs are less relevant to metabolic regulation and experiments. Many of these metabolites are prolifically abundant in the cell. While they are all essential

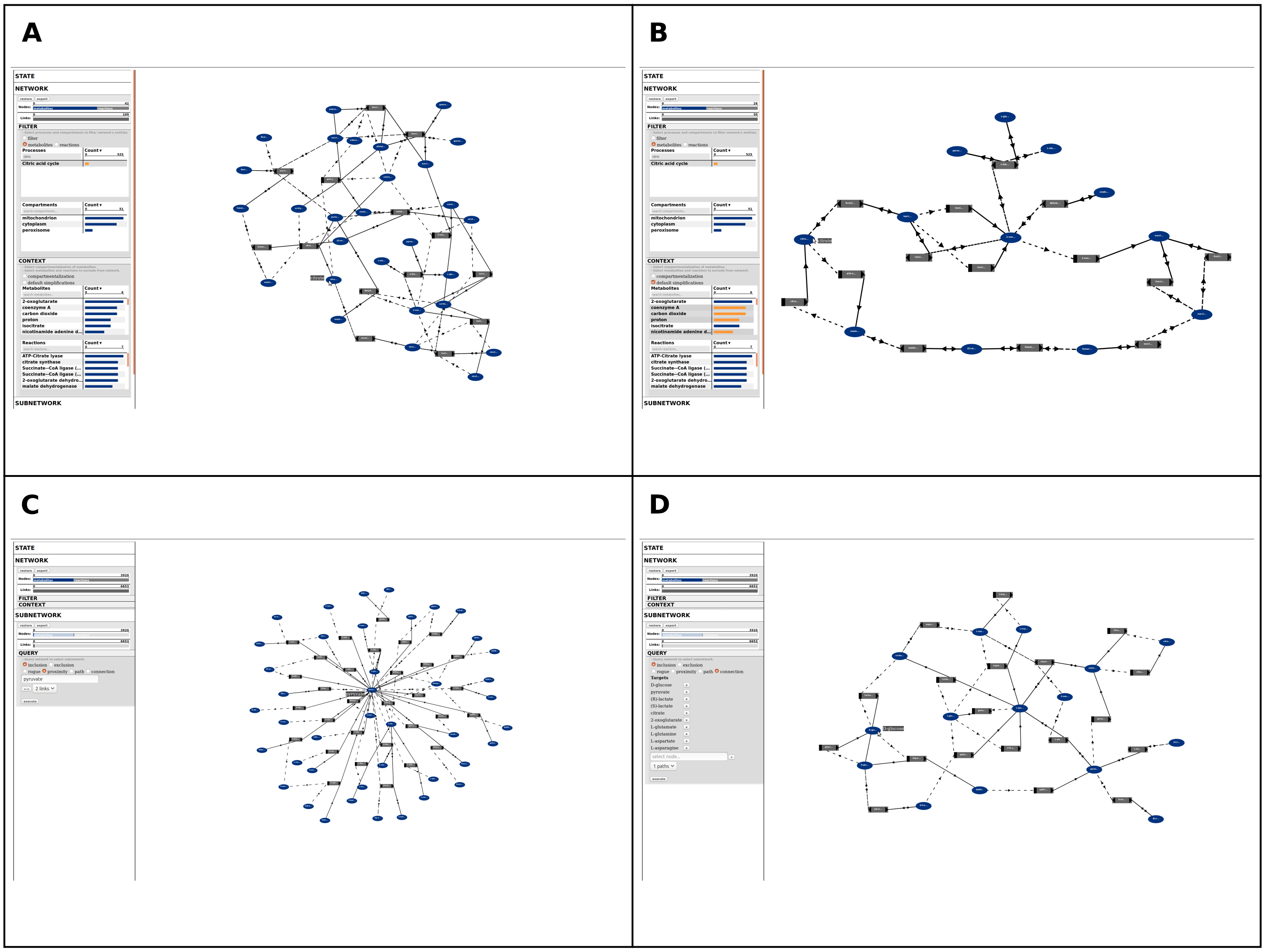

Figure 2. Screen shots of DyMetaboNet web application. DyMetaboNet defines and visualizes custom metabolic networks within the internet browser. **A.** With hubs, the Citric Acid Cycle has dense connectivity that obscures its cyclical structure. **B.** Exclusion of hubs coenzyme-A, carbon dioxide, proton, and nicotinamide adenine dinucleotide (NAD1+) reveals the overall cyclical structure of the Citric Acid Cycle. **C.** Queries by proximity include nodes within specific range of links to a focal node. For example, the user might need to know all reactions in which pyruvate participates. **D.** Pairwise queries for simple shortest paths allow construction of subnetworks between specific metabolites of interest. For example, the user might need to know how D-glucose, pyruvate, lactate, citrate, 2-oxoglutarate, glutamate, glutamine, aspartate, and asparagine relate to each other.

in the chemistry of metabolic reactions, some of these metabolites, such as water, dioxygen, and carbon dioxide, are unlikely to participate in the type of metabolic regulation that metabolic experiments commonly study. Perturbations in the abundance of these metabolites would be difficult to interpret. Also, some of these metabolites are undetectable in metabolomic measurements.

> Category 1 Hubs (**Table 2**): proton, water, dioxygen, phosphate, diphosphate, carbon dioxide, sulfate, hydrogen peroxide, ammonium, sulfite, sodium, hydrogen carbonate, hydroxide

Category 2 Hubs. Category 2 metabolite hubs are more relevant to metabolic regulation and experiments. The abundance of these metabolites in the cell fluctuates in metabolic regulation, and they are relevant to many metabolic experiments. However, some of these metabolites participate in so many reactions that they dominate connectivity in the metabolic network. Exclusion of these metabolites from the metabolic network reveals more subtle trends involving the influences of other metabolites that are of greater interest in some contexts.

> Category 2 Hubs (**Table 2**): coenzyme-A, acetyl coenzyme-A, acyl-carrier protein, carnitine, nicotinamide adenine dinucleotides, flavin adenine dinucleotides, nucleoside phosphates

This constraint for exclusion of specific metabolites is very sensitive and requires customization to the context of each metabolic experiment. Consequently, MetaboNet [40] makes the selection of these metabolites customizable. For our subsequent analyses herein, we chose to evaluate the extreme condition with exclusion of all metabolite hubs in Category 1 and all metabolite hubs in Category 2 with degrees greater than 50 (**Table 2**). Degree is a metric of a node's connectivity in a network, and we discuss it more later on. We found that the exclusion of these hubs simplified metabolic networks profoundly and exposed intrinsic structure that enhanced the potential to detect relevant clusters in our retrospective analyses of metabolomic measurements. This extreme approach may not be appropriate for all experiments, and metabolite hubs in Category 2 deserve particular attention in the

selection of metabolites for exclusion.

Web application to illustrate custom metabolic networks

DyMetaboNet [42] is a prototype application with basic and experimental functionality to illustrate concepts from MetaboNet [40] and explore ideas for future development. It uses source information about the human metabolic model from curation and adaptation in MetaboNet. DyMetaboNet is an accessory to our more extensive methods and analyses in MetaboNet.

We designed and developed the DyMetaboNet [42] web application to offer an accessible, convenient tool to define and explore custom metabolic networks. DyMetaboNet runs within the client's internet browser without the need for any server, and it is not necessary to install special software. DyMetaboNet provides a graphical interface with basic functionality to define and explore metabolic networks (**Figure 2**). The user selects filters for compartments and processes of interest to apply as filters. For example the user might want to consider only reactions and metabolites within the Mitochondrion compartment or those that participate in the Citric Acid Cycle process (**Figure 2B**). The user also selects whether to define a compartmental or non-compartmental network with (**Figure 2A**) or without (**Figure 2B**) common hubs. It is also possible to customize the selection of these hubs. From these parameters, DyMetaboNet defines custom metabolic networks using a similar method to MetaboNet, and presents a basic visual representation of these networks. With this integration of definition and visualization, DyMetaboNet makes it possible to explore interactively the effects of these parameters on metabolic networks. Furthermore, DyMetaboNet supports basic graph-traversal queries to select subnetworks by proximity (breadth-first search) (**Figure 2C**), shortest paths between source and target nodes (directional simple shortest paths), and pairwise shortest paths between multiple target nodes of interest (**Figure 2D**). A query by proximity would be useful where the user needs to know all reactions in which a single metabolite, such as pyruvate, participates (**Figure 2C**). A query by shortest paths would be useful where the user has measurements for 2 or more metabolites and needs to know how these relate to each other (**Figure 2D**). DyMetaboNet also exports tables of information about metabolites and reactions in these networks and subnetworks.

Analysis of metabolic networks

We applied multiple metrics from graph theory to describe our metabolic networks (**Table 3**), and we define these metrics here [41].

- Order: A network's order is its count of nodes, and bipartite networks have distinct counts for each of their two sets of nodes.
- Size: A network's size is its count of links between nodes, and bipartite networks only have links between nodes of different types.
- Density: A network's density is proportional to its size normalized to the maximal size possible in a comparable bipartite network. Networks with greater density have more interconnections, more links per nodes.
- Centrality: Individual nodes within a network have centralities. Degree centrality relates to a node's close-range influence, and it is proportional to the count of links that connect to the node. Betweenness centrality relates to a node's long-

Table 3. Properties of metabolic networks.

Compartments	Hubs	Order	Metabolites	Reactions	Size	Density	Centralization, Degree	Centralization, Between	Path	Cluster	Small World
True	True	6208	2735	3473	18809	9.901E-04	6.970E-05	5.262E-08	6.472E+00	1.051E-01	2.585E+02
True	False	5609	2428	3181	10003	6.476E-04	6.021E-06	2.073E-08	1.299E+01	1.454E-01	4.342 E+02
False	True	3908	1654	2254	13212	1.772E-03	2.944E-04	1.538E-07	4.742E+00	3.691E-02	6.948E+01
False	False	3711	1560	2151	6398	9.533E-04	1.837E-05	4.217E-08	1.457E+01	7.653E-02	1.557E+02

Graph metrics for alternative definitions of metabolic networks. Density, Centralization, Cluster Coefficient, and Small-World Coefficient are relative only to the bipartite set of nodes for metabolites.

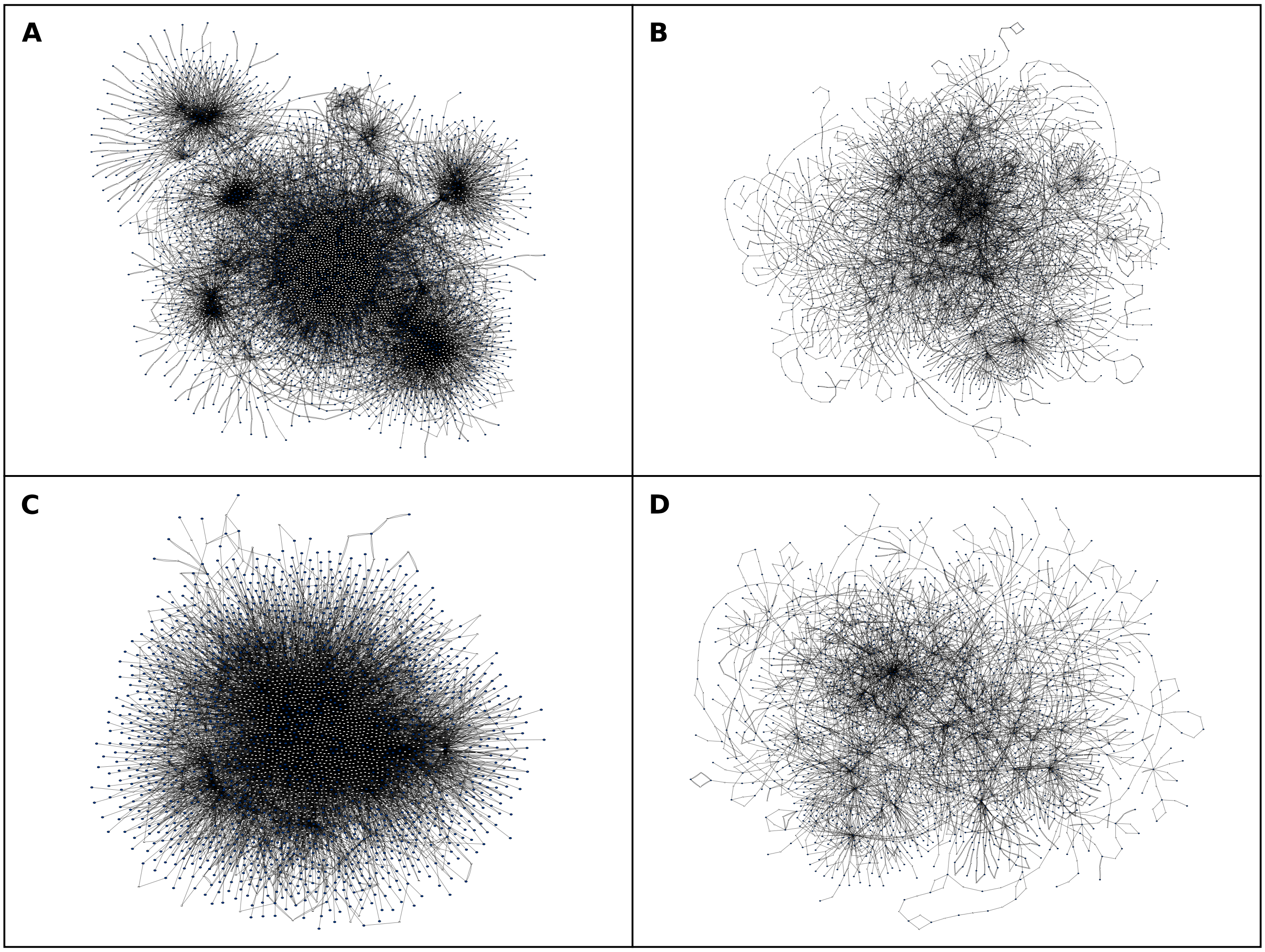

Figure 3. Global structures of metabolic networks. Alternative definitions of metabolic networks differ in global structure. Visual representations of metabolic networks in Cytoscape [43] with identical visual styles and layout parameters. **A.** Compartmental network with hubs. **B.** Compartmental network without hubs. **C.** Non-compartmental network with hubs. **D.** Non-compartmental network without hubs.

range influence. It is proportional to the count of shortest paths between all pairs of other nodes in a network that pass through the focal node. Together, degree and betweenness centralities are useful to rank or prioritize nodes in a network [44].

- Centralization: Centralization is the extent to which a network has a single, central node of maximal centrality, surrounded by all other nodes of minimal centrality. The star network defines maximal centralization.
- Cluster coefficient: Each of a network's nodes has a cluster coefficient to describe the local density of connections proximal to individual nodes.
- Mean cluster coefficient: A network has a mean cluster coefficient across all of its nodes.
- Mean shortest path: A network has a mean length of shortest paths between all pairs of nodes. This mean path length indicates the efficiency of communication, or the pervasiveness of signals.
- Small-world coefficient: Like random networks, small-world networks have small mean path lengths that scale proportionately to the natural logarithm of their orders [45]. However, unlike random networks, small-world networks have large mean cluster coefficients [45]. The sigma small-world coefficient [45] measures the small-world character of a network by comparing both mean cluster coefficient and mean path length to random bipartite networks with identical orders and sizes. A value of the sigma coefficient greater than 1 suggests that a network is small-world.

A bipartite network has distinct metrics of centrality, centralization, path-length, cluster coefficient, and small-world coefficient relative to each of its bipartite sets of nodes. Our metabolic networks have distinct metrics for metabolites and reactions. We chose to concentrate our analyses on these metrics relative to metabolites (**Table 3**), as our primary interest is in the flow of mass within the metabolic network and its measurement in metabolomic experiments.

Real systems commonly follow the pattern of a small-world network. Examples of small-word networks include friendships between people and connections between internet servers. In a small world of friendships, any person knows any other person vicariously through a small number of other people. In a small world of internet servers, any computer can communicate with any other computer by transferring information through

few intermediate servers. Small-world networks are especially interesting for their balance between modularity and short path lengths. This structure favors specialization and versatility while also allowing cooperative communication. These characteristics imply some combination of stochasticity and selection in the formation of these networks.

Like many other biological systems, metabolic networks are small-world [15]. All of our metabolic networks have values of the small-world coefficient much greater than 50 (**Table 3**). Also, both compartmental and non-compartmental networks with hubs have mean path lengths that are less than the natural logarithms of their orders (**Table 3**). This strong small-world character of metabolism suggests that the system relies heavily on modularity, but also that there is extensive and efficient communication and cooperation between modules [15]. Both signals and perturbations can pervade the entire system rapidly.

Compartmentalization confers major structural differences to our metabolic networks. The distinct global structures of compartmental and non-compartmental metabolic networks are strikingly apparent in visual representations (**Figure 3A,C**), and graph-theoretical metrics describe their differences in more detail (**Table 3**). Non-compartmental networks have lesser orders and sizes than their compartmental comparators (**Table 3**). This difference is partly due to the absence of replicate nodes for compartmental instances of chemically-identical metabolites and reactions (**Figure 1**). Also, non-compartmental networks exclude additional reactions that represent transport (**Figure 1**). Non-compartmental networks also have greater density and centralization than their compartmental comparators (**Table 3**). They also have lesser mean cluster coefficients and lesser small-world coefficients (**Table 3**). The non-compartmental network with hubs also has a lesser mean path length than the compartmental network with hubs (**Table 3**). These differences between compartmental and non-compartmental representations of metabolism have biological implications. Compartmentalization shifts metabolism towards less density and more dispersion, such that metabolites and reactions are further apart on average. Intracellular partitions are important to avoid excessive communication and interaction, such as through enzyme promiscuity and spurious allosteric interactions between metabolites and proteins [16]. Conversely, compartmentalization also shifts metabolism towards more modularity, allowing for specialization and regulation within separate chemical environments. Surprisingly, this increase in modularity out-balances the increase in dispersion such that compartmentalization actually enhances the small-world character of metabolic networks, at least by the metric of the sigma coefficient.

Inclusion or exclusion of metabolite hubs also distinguishes major differences in global structures of our metabolic networks. These differences are apparent in their visual representations (**Figure 3C,D**), and in their metrics (**Table 3**). Networks without hubs have lesser orders and sizes than their comparators (**Table 3**) due to the exclusion of nodes for metabolites that qualify as hubs (**Table 2**). Additionally, networks without hubs have lesser density and centralization than their comparators (**Table 3**). These differences are intuitive as hubs themselves are central and contribute greatly to the connectivity of their networks. Networks without hubs also have greater mean path lengths, greater mean cluster coefficients, and greater small-world coefficients (**Table 3**). Without hubs, mean path lengths increase more than 2-fold for the compartmental network and more than 3-fold for the non-compartmental network (**Table 3**). This dramatic change suggests that these hubs dominate the majority of shortest paths in their networks. Conversely, the exclusion of these hubs reveals greater modularity both in compartmental and non-compartmental networks (**Table 3**). Surprisingly, this increase in modularity out-balances the increase in dispersion such that exclusion of hubs actually enhances the small-world character of metabolic networks, at least by the metric of the sigma coefficient (**Table 3**). These latter differences between representations of metabolism with and without metabolite hubs are biologically interesting. These metabolite hubs dominate the structure and behavior of metabolic networks so dramatically that they obscure much of the small-world behavior within the rest of the network. The selection of hub metabolites for exclusion (**Table 2**) from the network is very important as the effect of this simplification is profound.

Influence of metabolites in metabolic networks

Our definitions of metabolic networks differ also in their profiles of dominant metabolites. Our metabolic networks are roughly scale-free [15], as nodes' degrees follow roughly an exponential distribution (**Figure 4A,F**). Exclusion of hubs has its greatest effect on those few nodes with the greatest degrees (**Figure 4A,F**). We explored the effects of this exclusion of hubs on the relative influence or weight of metabolites within these networks. Our rankings of metabolites' influences (**Figure 4B-C,G-H**) combines their close-range degree centrality and their long-range betweenness centrality [44]. In both compartmental and non-compartmental networks, the exclusion of hubs changes dramatically the metabolites with dominant influences (**Figure 4B-E,G-J**). Exclusion of hubs such as proton, water, coenzyme A, nicotinamide adenine dinucleotides, adenosine triphosphate, hydrogen phosphate, and dioxygen (**Figure 4B,D,G,I**) allows for other metabolites to rise to prominence, such as glutamate, pyruvate, glycine, oxoglutarate, and cholesterol (**Figure 4C,E,H,J**).

Glutamate, as a particularly promiscuous non-hub metabolite, is an impressive example of connection and cooperation in metabolism. In both the compartmental and non-compartmental networks without hubs, glutamate is the top-ranking metabolite in terms of both its close and long-range influence (**Figure 4C,H**). In our non-compartmental metabolic network without hubs, this amino acid participates in 67 reactions in 3 different cellular compartments, belonging to 30 different metabolic processes. Glutamate belongs to 25 different sets within MetaboAnalyst's default library for metabolite set enrichment analysis [46]. Whereas analyses of sets isolate Glutamate's various roles, analyses of networks integrate these for a holistic perspective. Glutamate illustrates the importance of studying metabolism as an entire system, not as arbitrarily separate sets of distinct pathways. Perturbations of this central metabolite are likely to have profound effects on the system, but they are also difficult to interpret.

Glutamate's metabolic sets [46]

Malate-Aspartate Shuttle; Glucose-Alanine Cycle; Alanine Metabolism; Glutathione Metabolism; Cysteine Metabolism; Phenylalanine and Tyrosine Metabolism; Folate Metabolism; Urea Cycle; Lysine Degradation; Ammonia Recycling; Amino Sugar Metabolism; Beta-Alanine Metabolism; Aspartate Metabolism; Nicotinate and Nicotinamide Metabolism; Propanoate Metabolism; Histidine Metabolism; Glutamate Metabolism; Arginine and Proline Metabolism; Warburg Effect; Glycine and Serine Metabolism; Tryptophan Metabolism; Valine, Leucine and Isoleucine Degradation; Arachidonic Acid Metabolism; Tyrosine Metabolism; Purine Metabolism

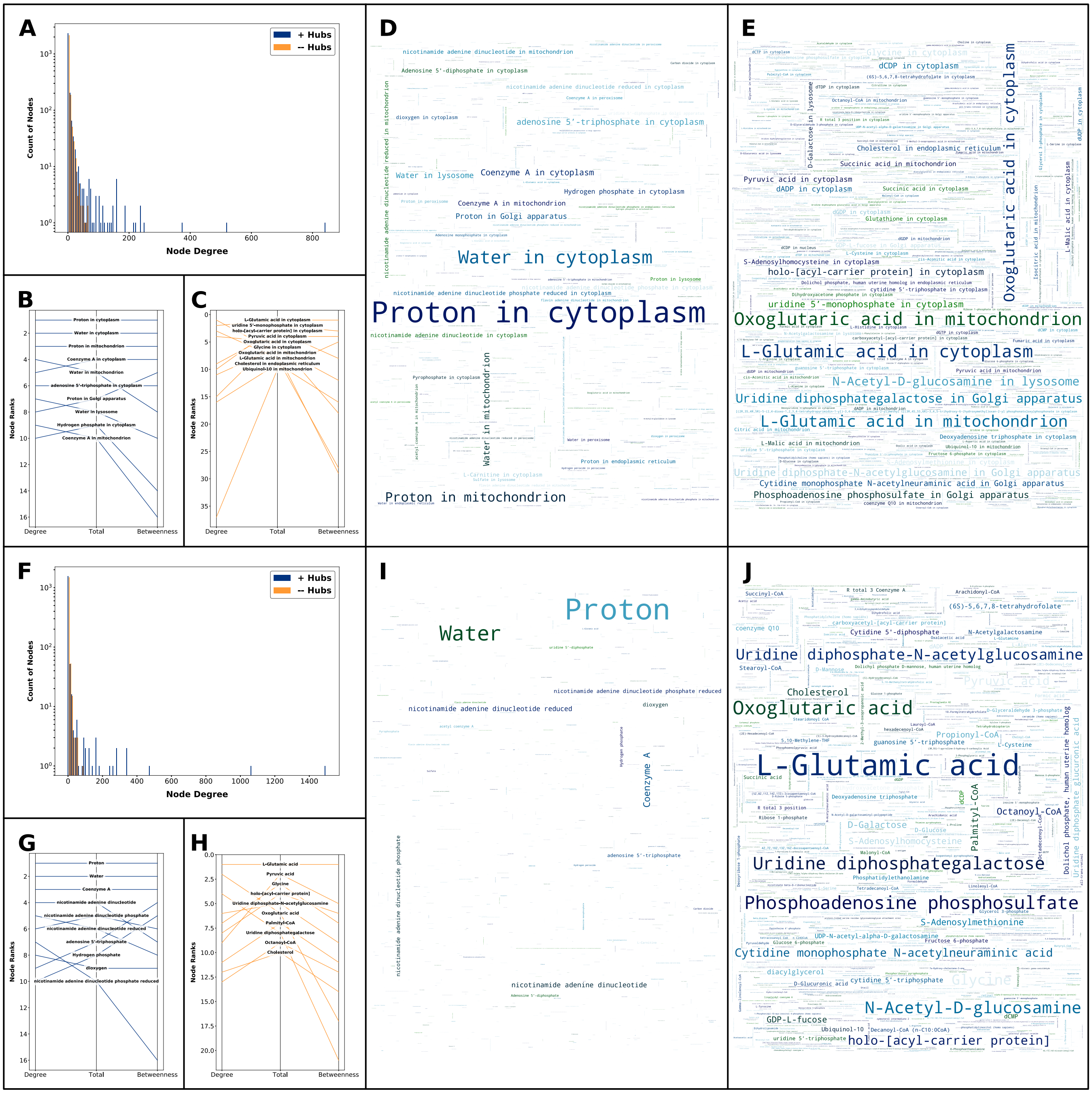

Figure 4. Properties of metabolites' nodes in metabolic networks. Alternative definitions of metabolic networks differ in dominant influence of metabolites' nodes. **A–E.** Compartmental metabolic networks. **F–J.** Non-compartmental metabolic networks. **A, F.** Histograms for counts of metabolite nodes with specific degrees in networks with and without hubs. **B, C, G, H.** Parallel coordinates charts for ranks of metabolites' nodes in metabolic networks by degree centrality ("Degree"), betweenness centrality ("Betweenness"), or a mean of these ranks ("Total"). **B, G.** Ranks of metabolites' nodes in metabolic networks with hubs. **C, H.** Ranks of metabolites' nodes in metabolic networks without hubs. **D, E, I, J.** Word cloud visual representations of influences of metabolites' nodes in metabolic networks with nodes' degrees scaled to font size by a factor of 1.0. **D, I.** Influences of metabolites' nodes in metabolic networks with hubs. **E, J.** Influences of metabolites' nodes in metabolic networks without hubs.

Application of metabolic network to metabolomic experiments

Our purpose in the definition and analysis of metabolic networks was to evaluate their suitability for metabolomic experiments. An appropriate network has potential to facilitate design of experiments and interpretations of metabolomic measurements. It is important for this network to give appropriate context, and proper constraints by compartmentalization and hubs can simplify this context.

Our analyses suggest that the non-compartmental network without hubs is the most appropriate in which to integrate and ex-

plore metabolomic measurements. Standard metabolomic experiments do not distinguish between cellular compartments; rather, a measurement for an analyte, such as glutamate, represents the total abundance of that analyte in all types of cells and subcellular organelles in a sample. Mapping non-compartmental metabolomic measurements onto a compartmental metabolic network would require some type of replication for compartmental instances of each metabolite. It would be difficult or impossible for this replication of measurements to represent compartmental pools of the metabolite accurately, and there is a risk that this replication of measurements would introduce artifacts or bias. For this reason, we chose the non-compartmental metabolic network for integration and analysis of metabolomic measurements. Furthermore, the exclusion of metabolite hubs has potential to enhance the detection of clusters of measurements for metabolites of interest. Hub metabolites dominate connections in the network (**Table 3**) and are central to the majority of shortest paths in the network (**Table 3**). Hence hubs would also dominate topological queries to analyze measurements on the network. For example, knowledge that two analytes relate to each other directly via water would be unhelpful. Hubs also obscure modularity in the network (**Table 3**), suggesting that they would interfere with detection of clusters of measurements on the network. Case-specific selection of metabolite hubs for exclusion simplifies the network to emphasizes the influence of relevant metabolites (**Figure 4C,H**). For these reasons, we chose the metabolic network without hubs for integration and analysis of metabolomic measurements.

Preparation of metabolomic measurements

Having selected the non-compartmental metabolic network without hubs, we prepared to evaluate its utility in real analyses of metabolomic measurements. We searched for publicly-accessible metabolomic measurements from studies that were appropriate for our human metabolic model. After selecting these studies, we processed and analyzed measurements generally and by the standard set-enrichment strategy before integrating them with the network.

We selected multiple previous studies with publicly-available metabolomic measurements for further retrospective analyses. As our model and networks represent human intracellular metabolism, we selected studies of human cellular tissues, rather than plasma, serum, other body fluids, or excrement. We selected 5 studies with samples from fat, lung, liver, and muscle tissues, all from human subjects (**Table 4**) [47, 48, 49, 50, 51, 52, 53]. Each of these studies includes measurements for 125 or more identifiable analytes, of which at least 70 (56%) match to metabolites in our model of human metabolism (**Table 4**). Studies 1, 2, and 5 are of particular interest as they utilize pairs of dependent samples from the same patients in each experimental group. Studies 1, 2, 3, and 4 have previous publications that analyze and interpret trends in metabolites [47, 49, 51].

We processed these measurements to compare experimental groups in each study. We normalized measurements to total signals in each sample and then determined the differential abundance of individual metabolites. We calculated the base-2 logarithms of fold changes and p-values to compare metabolites between experimental groups (**Table 4**). For each study, we visualized these fold changes and p-values simultaneously in volcano plots (**Figure 5A,E,I,M,Q**). These plots effectively emphasize metabolites with both great differential abundance and great precision in their measurements. While our method of processing these measurements was simple and general, we observed trends of accumulation and depletion in metabolites (**Figure 5A,E,I,M,Q**) that were consistent with those in previous publications of these studies [47, 49, 51].

Analysis of metabolomic measurements in sets

Metabolite set enrichment analysis remains an accessible and convenient method to interpret metabolomic measurements within biological context. This analytical strategy is a foundation of the widely-used platform, MetaboAnalyst [46]. We found that MetaboAnalyst's visual, step-wise interface on the web was accessible, convenient, and easy to use. Analysts upload tables of measurements for each metabolite in samples within each experimental group. MetaboAnalyst processes these measurements, compares experimental groups, and evaluates enrichment of metabolites in functionally-relevant sets. MetaboAnalyst's default set library includes 99 sets of metabolites that participate in common metabolic processes [46]. We included this strategy in our retrospective analysis of metabolomic measurements as a comparison to a current standard.

Metabolite set enrichment analysis in MetaboAnalyst [46], demonstrated limitations in this strategy. In this analysis, several sets ranked highly as hits even when measurements were only available for a small fraction of their total metabolites. In the most extreme example for Study 1, a set of 35 metabolites had measurements for only 1 matching metabolite (**Table 5**). This set ranked in the top 5 hits with a p-value less than 1E-06 (**Table 5**). Also in this analysis, a few metabolites dominated multiple metabolite sets that were top hits. For Studies 1-5, a single metabolite belonged to at least the top 3 of 5 sets (**Table 5**). For Studies 1, 2, 4, and 5, a single metabolite belonged to at least the top 4 of 5 sets (**Table 5**). For Studies 1, 2, and 4, all 5 top sets included the same metabolite (**Table 5**). This overlap between sets and the tendency for a small count of metabolites to dominate top hits risks over-interpretation of artifactual measurements and false-positives.

Analysis of metabolomic measurements in network clusters

We finally applied our custom human metabolic network to retrospective analyses of metabolomic measurements. We previously described how we developed and applied our tools in MetaboNet [40] to define the non-compartmental metabolic network without hubs. We found this network to be appropriate to provide context in metabolomic experiments. We also processed metabolomic measurements and evaluated them both generally and by the standard strategy of metabolite set enrichment analysis [46].

To detect clusters of measurements on our metabolic network, we applied general functionality from Cytoscape [43]. Cytoscape [43] was less accessible and convenient to use than MetaboAnalyst [46], as Cytoscape required local installation, installation of the jActiveModules application [54, 55], and familiarization with many settings for both analyses and visualizations. Still, Cytoscape and jActiveModules provided a specific and consistent protocol to search for preliminary clusters of measurements. The jActiveModules algorithm [54] detects enrichment of p-values across multiple nodes in clusters across the network, and this combination is sensitive even to weak differences in measure-

Table 4. Curation of metabolomic measurements.

Study	Tissue	Group, Dividend	Group, Divisor	Pairs	Analytes	Metabolites	Project	Study	Reference
1	Adipose	Viscera	Subcutane	True	132	91 (68.94%)	PR000058	ST000061	[47, 48]
2	Lung	Tumor	Normal	True	177	120 (67.80%)	PR000305	ST000390	[49, 50]
3	Liver	Ischemia	Normal	False	151	109 (72.19%)	PR000322	ST000412	[51, 52]
4	Liver	Steatosis	Normal	False	151	109 (72.19%)	PR000322	ST000412	[51, 52]
5	Muscle	Exercise	Obese	True	125	70 (56.00%)	PR000599	ST000842	[53]

All studies use tissues from people of species Homo sapiens. Fold change calculations represent quotients of measurements from dividend (numerator) groups to divisor (denominator) groups. Information from all studies is accessible on Metabolomics Workbench (cite) in specific records for each project and study.

ments.

Integration and analysis of metabolomic measurements with our metabolic network discovered multiple unique clusters suggestive of specific perturbations at individual reactions. We matched analytes from metabolomic studies to nodes for metabolites in our metabolic network. To these nodes we integrated the fold changes and p-values that we previously calculated to compare the measurements of each metabolite between experimental groups in each study (**Table 4**). We applied the jActiveModules algorithm [54] in Cytoscape [43] to detect preliminary clusters with metabolites and reactions of interest. As this search algorithm and its clusters base on the general enrichments of p-values, we integrated fold changes for an additional dimension in the search. We critiqued and curated these clusters for biological relevance, with particular interest in patterns of both accumulation and depletion in metabolites that related closely by proximal reactions (**Figure 5, Table 6**). These patterns implicated individual reactions in differential regulation between experimental groups (**Table 7**). We identified genes and proteins for enzymes in these reactions and consulted Entrez Gene [5] and UniProt [30] databases to contextualize their roles and regulation in metabolism (**Table 7**).

Here we describe some of the more interesting clusters we found. For the sake of brevity, we limit our discussion to clusters from Studies 1, 2, and 5. We also discuss potential hypotheses and interpretations of these clusters. Importantly, our exploratory analyses of these studies are preliminary and are most suitable for the generation of speculative hypotheses.

Study 1: Visceral versus Subcutaneous Adipose

Visceral and subcutaneous white adipose tissues differ both in their roles in metabolism and in their contributions to morbidity in obesity [56]. Visceral and subcutaneous fat depots develop from distinct cellular lineages and grow in separate parts of the body [57, 58, 56]. Whereas subcutaneous adipose grows between the skin and muscle, visceral adipose grows within the abdominal cavity around internal organs. These fat depots also contribute differently to the morbidity of obesity, with visceral adipose promoting more inflammation and also imparting greater risk for metabolic syndrome, including diabetes, cardiovascular disease, and even cancer [57, 58, 56]. Study 1 collected samples of visceral and subcutaneous adipose tissues from 59 human patients during surgery for colon cancer [47]. The publication of Study 1 emphasized several observations from integration of metabolomic and transcriptomic measurements on visceral adipose relative to subcutaneous adipose [47]. These observations were consistent with a primary role of visceral adipose as a metabolically-active endocrine organ and a primary role of subcutaneous adipose as a metabolically-dormant storage organ [47].

Cluster 1: Glucosidase alpha. Study 1, Cluster 1 (**Figure 5B**) suggests that Glucosidase alpha has differential activity in visceral relative to subcutaneous adipose, and this difference might be a selective therapeutic target in diabetes. Relative to subcutaneous adipose, visceral adipose had depletion of glucose (p-value: **2.557E-02**) and accumulation of both maltotriose (p-value: **1.229E-03**) and maltose (p-value: **1.942E-06**) (**Figure 5B, Table 6**). Glucosidase alpha in this cluster degrades polysaccharides to release glucose. Its activity is especially important to degrade complex sugars in the intestine, and it also functions in liver and muscle tissues to degrade glycogen and release glucose. A class of anti-diabetic drugs inhibit intestinal Glucosidase alpha to moderate the postprandial (after meal) absorption of glucose [58]. One member of this class of inhibitors of Glucosidase alpha, Miglitol, also moderates metabolic syndrome in obesity, partly by direct influence on both brown and white adipose tissues [59]. Visceral adipose absorbs more glucose than does subcutaneous adipose [57], and if the activity of Glucosidase alpha differs between visceral and subcutaneous white adipose as this cluster suggests, then Miglitol might also influence these depots selectively. The original publication of this study acknowledged depletion of glucose but did not emphasize Glucosidase alpha as a candidate enzyme of interest [47]. The metabolite set "Glycolysis" most closely represents this cluster, and this set ranked 10th in metabolite set enrichment analysis.

Cluster 2: Branched-chain amino acid transaminase. Study 1, Cluster 2 (**Figure 5C**) suggests that Branched-chain amino acid (BCAA) transaminase has differential activity in visceral relative to subcutaneous adipose, and this difference might contribute to morbidity in obesity. Relative to subcutaneous adipose, visceral adipose had accumulation of all three BCAAs, leucine (p-value: **5.915E-06**), isoleucine (p-value: **5.240E-05**), and valine (p-value: **3.764E-04**) (**Figure 5C, Table 6**). There was also a slight depletion of ketoleucine (p-value: **6.385E-02**), the initial product of leucine degradation (**Figure 5C, Table 6**). The enzyme BCAA transaminase in this cluster catalyzes the first reaction in degradation of the BCAAs. Metabolic syndrome in obesity correlates with BCAA accumulation, partly attributable to a decrease in the expression of enzymes for BCAA degradation in visceral

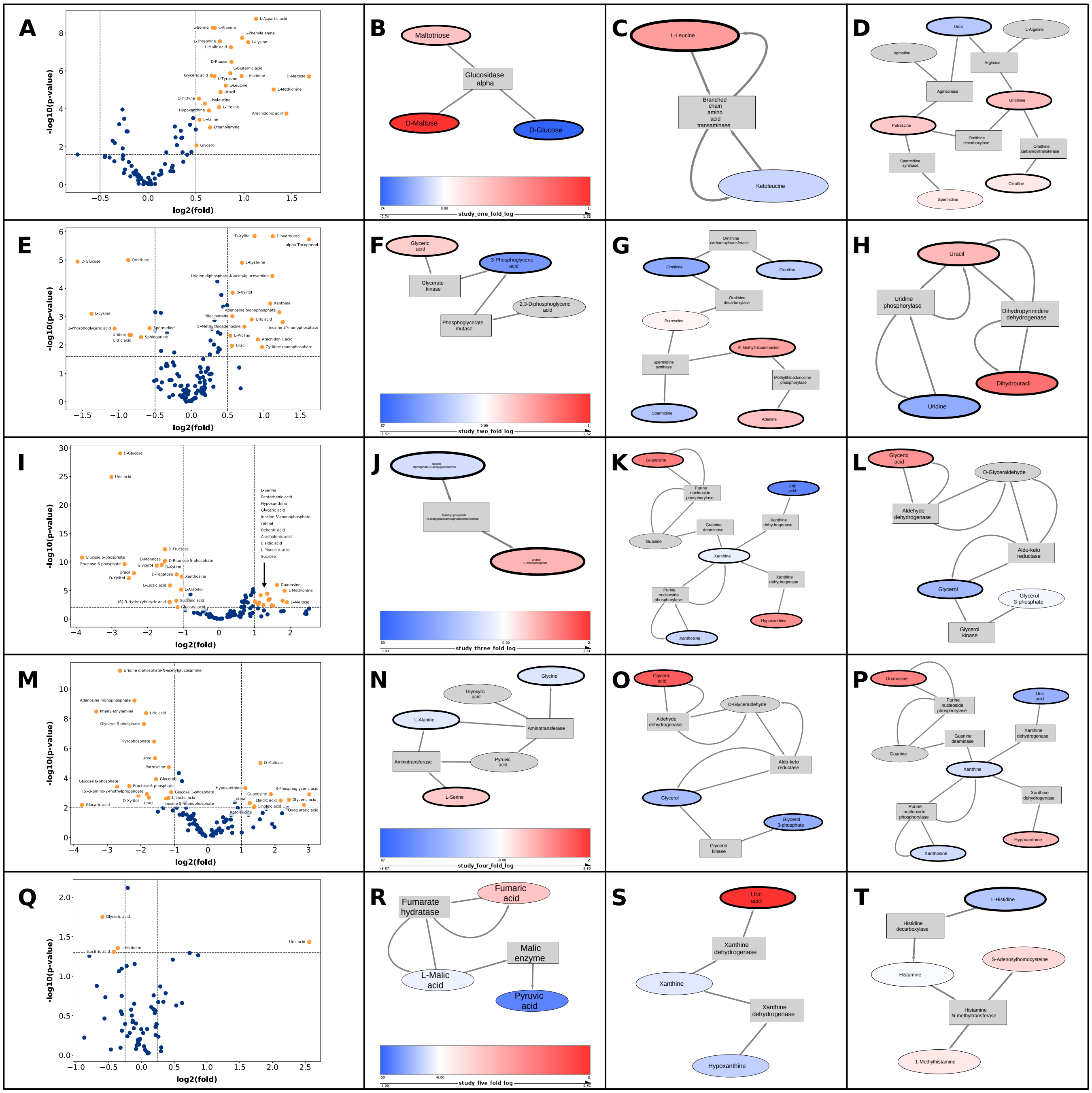

Figure 5. Integration and analysis of metabolomic measurements on metabolic networks. Functional clusters of enrichment in metabolomic measurements are detectable by integration with metabolic networks. **A–D.** Study 1. **E–H.** Study 2. **I–L.** Study 3. **M–P.** Study 4. **Q–T.** Study 5. **A, E, I, M, Q.** Volcano plots for p-values and fold changes of analytes in metabolomic measurements. **B–D, F–H, J–L, N–P, R–T.** Clusters in metabolic network detectable by enrichment of p-values with fill color representation of fold change and border width representation of significance (p-value < 0.05).

adipose tissue [60, 61, 62]. BCAAs influence the master nutrient sensor, Mammalian Target of Rapamycin (mTOR), which in turn regulates anabolism and catabolism of glucose and lipids [63]. The original publication of this study acknowledged accumulation of most amino acids but did not acknowledge accumulation of BCAAs specifically or emphasize BCAA transaminase as a candidate enzyme of interest [47]. The metabolite set "Valine, Leucine and Isoleucine Degradation" most closely represents this cluster, and this set ranked 71st in metabolite set enrichment analysis.

Cluster 3: Arginase. Study 1, Cluster 3 (**Figure 5D**) suggests that Arginase has differential activity in visceral relative to subcutaneous adipose, and this difference might exacerbate inflammation, oxidative stress, and insulin resistance. Relative to subcutaneous adipose, visceral adipose had depletion of urea (p-value: **2.795E-02**) and accumulation of both ornithine (p-value: **2.859E-05**) and putrescine (p-value: **1.900E-02**) (**Figure 5D, Table 6**). Arginase in this cluster converts arginine to urea and ornithine in the last reaction of the Urea Cycle [64]. Urea is a waste

Table 5. Metabolite Set Enrichment Analysis

Study 1	Name	P-Value	Total	Hits	Metabolites
-	Fatty Acid Biosynthesis	8.694E-07	35	6	Palmitic acid, Myristic acid, 3-Hydroxybutyric acid, Caprylic acid, Capric acid, Dodecanoic acid
-	Fatty Acid Elongation in Mitochondria	9.679E-07	35	1	Palmitic acid
-	Fatty Acid Metabolism	9.691E-07	43	3	Palmitic acid, Adenosine monophosphate, Pyrophosphate
-	Steroid Biosynthesis	9.696E-07	48	2	Palmitic acid, Pyrophosphate
-	Bile Acid Biosynthesis	1.054E-06	65	4	Palmitic acid, Pyrophosphate, Taurine, Glycine
**Study 2**	**Name**	**P-Value**	**Total**	**Hits**	**Metabolites**
-	Lysine Degradation	3.961E-06	30	6	L-Glutamic acid, L-Lysine, FAD, L-Pipecolic acid, Aminoadipic acid, Oxoglutaric acid
-	Beta-Alanine Metabolism	8.478E-05	34	8	Dihydrouracil, L-Glutamic acid, L-Histidine, Uracil, FAD, L-Aspartic acid, Oxoglutaric acid, Beta-Alanine
-	Nicotinate and Nicotinamide Metabolism	8.569E-05	37	6	L-Glutamic acid, Adenosine monophosphate, Niacinamide, FAD, Phosphoric acid, L-Glutamine
-	Cysteine Metabolism	8.840E-05	26	4	L-Glutamic acid, Adenosine monophosphate, Phosphoric acid, Oxoglutaric acid
-	Warburg Effect	8.955E-05	58	12	L-Glutamic acid, 3-Phosphoglyceric acid, Citric acid, L-Malic acid, FAD, Phosphoric acid, Succinic acid, Fructose 6-phosphate, Glucose 6-phosphate, L-Glutamine, Fumaric acid, Oxoglutaric acid
**Study 3**	**Name**	**P-Value**	**Total**	**Hits**	**Metabolites**
-	Gluconeogenesis	1.030E-30	35	6	Beta-D-Glucose, Glucose 6-phosphate, Oxoglutaric acid, Malic acid, Phosphoric acid, 3-Phosphoglyceric acid
-	Trehalose Degradation	1.032E-30	11	1	Beta-D-Glucose
-	Glycolysis	1.034E-30	25	4	Beta-D-Glucose, Glucose 6-phosphate, Phosphoric acid, 3-Phosphoglyceric acid
-	Nucleotide Sugars Metabolism	1.293E-11	20	2	Glucose 6-phosphate, Uridine diphosphate glucuronic acid
-	Starch and Sucrose Metabolism	1.404E-11	31	6	D-Fructose, Glucose 6-phosphate, Beta-D-Fructose 6-phosphate, Sucrose, Uridine diphosphate glucuronic acid, 3-Phosphoglyceric acid
**Study 4**	**Name**	**P-Value**	**Total**	**Hits**	**Metabolites**
-	Butyrate Metabolism	1.830E-12	19	2	Adenosine monophosphate, Succinic acid
-	Mitochondrial Beta-Oxidation of Medium Chain Saturated Fatty Acids	1.217E-10	27	2	Adenosine monophosphate, Dodecanoic acid
-	Ethanol Degradation	1.226E-10	19	1	Adenosine monophosphate
-	Mitochondrial Beta-Oxidation of Short Chain Saturated Fatty Acids	1.226E-10	27	1	Adenosine monophosphate
-	Riboflavin Metabolism	1.226E-10	20	1	Adenosine monophosphate
**Study 5**	**Name**	**P-Value**	**Total**	**Hits**	**Metabolites**
-	Spermidine and Spermine Biosynthesis	1.430E-02	18	1	L-Methionine
-	Betaine Metabolism	1.430E-02	21	3	L-Methionine, NAD, S-Adenosylhomocysteine
-	Methionine Metabolism	1.430E-02	43	3	L-Methionine, NAD, S-Adenosylhomocysteine
-	Glycine and Serine Metabolism	1.988E-02	59	7	L-Methionine, Glyceric acid, Pyruvic acid, NAD, Creatine, Oxoglutaric acid, S-Adenosylhomocysteine
-	Phospholipid Biosynthesis	4.631E-02	29	3	DG(16:0/16:0/0:0), LysoPC(16:0), NAD

Metabolite set enrichment analysis in MetaboAnalyst [46]. Top 5 ranking sets for each study ranked by p-value. Column "Total" reports the cardinality or total count of metabolites in each set. Column "Hits" reports the count of metabolites from the set with measurements.

product to eliminate amines from the body, while ornithine supplies synthesis of proline and polyamines for cellular proliferation [64]. Arginase's activity also reduces the availability of arginine for synthesis of nitric oxide by Nitric oxide synthase [64].

Table 6. Cluster Metabolites

Study	Cluster	Analyte	Log-2 Fold	P-Value
1	1	Maltotriose	4.978E-01	1.229E-03
1	1	D-Glucose	-7.350E-01	2.557E-02
1	1	D-Maltose	1.679E+00	1.942E-06
1	2	L-Leucine	8.110E-01	5.915E-06
1	2	Ketoleucine	-1.997E-01	6.385E-02
1	2	L-Isoleucine	5.929E-01	5.240E-05
1	2	L-Valine	5.378E-01	3.764E-04
1	3	Citrulline	1.910E-01	1.960E-02
1	3	Ornithine	5.311E-01	2.859E-05
1	3	Putrescine	4.528E-01	1.900E-02
1	3	Urea	-2.626E-01	2.795E-02
1	3	Spermidine	1.773E-01	8.122E-02
2	1	Glyceric acid	3.996E-01	4.002E-03
2	1	3-Phosphoglyceric acid	-1.057E+00	2.599E-03
2	2	Citrulline	-4.962E-01	6.918E-04
2	2	Ornithine	-8.687E-01	1.011E-05
2	2	Putrescine	9.096E-02	4.030E-01
2	2	Spermidine	-5.751E-01	2.531E-03
2	2	5'-Methylthioadenosine	7.321E-01	2.242E-03
2	2	Adenine	4.932E-01	3.919E-04
2	3	Dihydrouracil	1.122E+00	1.449E-06
2	3	Uracil	5.588E-01	1.057E-02
2	3	Uridine	-8.510E-01	4.385E-03
3	1	Uridine diphosphate-N-acetylglucosamine	-7.965E-01	4.822E-05
3	1	Uridine 5'-monophosphate	9.399E-01	6.016E-03
3	2	Guanosine	1.626E+00	1.066E-06
3	2	Xanthine	-3.705E-01	2.079E-02
3	2	Hypoxanthine	1.420E+00	2.893E-04
3	2	Xanthosine	-1.045E+00	3.631E-08
3	2	Uric acid	-3.008E+00	1.205E-25
3	3	Glyceric acid	1.389E+00	4.517E-04
3	3	Glycerol	-1.741E+00	4.139E-10
3	3	Glycerol 3-phosphate	-2.083E-01	2.727E-01
4	1	L-Serine	7.594E-01	2.077E-02
4	1	L-Alanine	-5.580E-01	4.062E-03
4	1	Glycine	-6.363E-01	9.534E-03
4	2	Glyceric acid	2.284E+00	3.472E-03
4	2	Glycerol	-1.671E+00	5.360E-05
4	2	Glycerol 3-phosphate	-2.033E+00	6.463E-09
4	3	Guanosine	1.745E+00	1.674E-03
4	3	Xanthine	-8.043E-01	9.214E-04
4	3	Hypoxanthine	9.819E-01	1.233E-03
4	3	Xanthosine	-9.010E-01	2.006E-05
4	3	Uric acid	-1.974E+00	1.574E-09
5	1	Fumaric acid	7.357E-01	5.084E-02
5	1	L-Malic acid	-8.227E-02	8.337E-01
5	1	Pyruvic acid	-7.935E-01	5.513E-02
5	2	Hypoxanthine	-3.013E-01	1.777E-01
5	2	Xanthine	-1.462E-01	3.144E-01
5	2	Uric acid	2.562E+00	3.688E-02
5	3	L-Histidine	-3.593E-01	4.395E-02
5	3	Histamine	-3.444E-02	7.294E-01
5	3	1-Methylhistamine	2.873E-01	1.348E-01
5	3	S-Adenosylhomocysteine	4.790E-01	6.171E-02

Differential abundance in metabolomic measurements of metabolites in or relevant to clusters.

Via both polyamines and nitric oxide, Arginase correlates with inflammation and oxidative stress [64]. Inhibition of Arginase ameliorates inflammation and insulin resistance in diabetes and obesity [64]. The original publication of this study did not em-

Table 7. Cluster Reactions

Study 1	Cluster	Reaction	Gene Identifiers	Protein Identifiers
-	1	**Glucosidase alpha**	2548, 2595	P10253, Q8TET4
-	2	**Branched chain amino acid transaminase**	586, 587	
-	3	Ornithine carbamoyltransferase	5009	P00480
-	3	Ornithine decarboxylase	4953	P11926
-	3	**Arginase**	383, 384	P05089, P78540
-	3	Agmatinase	79814	Q9BSE5
-	3	Spermidine synthase	6723	P19623

Study 2	Cluster	Reaction	Gene Identifiers	Protein Identifiers
-	1	**Glycerate kinase**	132158	Q8IVS8
-	1	Phosphoglycerate mutase	669, 5223, 5224	P07738
-	2	Ornithine carbamoyltransferase	5009	P00480
-	2	Ornithine decarboxylase	4953	P11926
-	2	**Spermidine synthase**	6723	P19623
-	2	Methylthioadenosine phosphorylase	4507	Q13126
-	3	Dihydropyrimidine dehydrogenase	1806	
-	3	**Uridine phosphorylase**	7378, 151531	

Study 3	Cluster	Reaction	Gene Identifiers	Protein Identifiers
-	1	Dolichyl-phosphate N-acetyl-glucosamine-phospho-transferase	1798	
-	2	Purine nucleoside phosphorylase	4860	
-	2	Guanine deaminase	9615	
-	2	Xanthine dehydrogenase	7498	P47989
-	3	Aldehyde dehydrogenase	217, 219, 223, 224, 501	
-	3	Aldo-keto reductase	231, 10327	
-	3	Glycerol kinase	2710, 2712	

Study 4	Cluster	Reaction	Gene Identifiers	Protein Identifiers
-	1	Aminotransferase	189	
-	2	Aldehyde dehydrogenase	217, 219, 223, 224, 501	
-	2	Aldo-keto reductase	231, 10327	
-	2	Glycerol kinase	2710, 2712	
-	3	Purine nucleoside phosphorylase	4860	
-	3	Guanine deaminase	9615	
-	3	Xanthine dehydrogenase	7498	P47989

Study 5	Cluster	Reaction	Gene Identifiers	Protein Identifiers
-	1	Fumarate hydratase	2271	P07954
-	1	**Malic enzyme**	4199, 4200, 10873	P48163, P23368, Q16798
-	2	**Xanthine dehydrogenase**	7498	P47989
-	3	**Histidine decarboxylase**	3067	
-	3	Histamine N-methyltransferase	3176	

Reactions in clusters. Bold font denotes reactions of special interest in each cluster. Gene identifiers match records in Entrez Gene [5]. Protein identifiers match records in UniProt [30].

phasize Arginase as a candidate enzyme of interest [47]. The metabolite set "Urea Cycle" most closely represents this cluster, and this set ranked 23rd in metabolite set enrichment analysis.

Study 2: Cancerous versus Normal Lung

Many types of cancer alter metabolic flux dramatically to enhance macromolecular biosynthesis and thereby sustain cellular proliferation in tumors [8, 9]. Tumors also often experience microenvironments of hypoxia [8]. Study 2 collected samples of early-stage adenocarcinoma tumors and adjacent nonmalignant tissue from the lungs of 39 human patients who were either current or former smokers [49]. The publication of Study 2 emphasized several observations from metabolomic measurements on cancerous lung relative to normal lung [49]. There was evidence of enhanced synthesis of nucleotides and increased response to reactive oxygen species along with surprisingly sparse synthesis of polyamines [49].

Cluster 1: Glycerate kinase. Study 2, Cluster 1 (**Figure 5F**) suggests that Glycerate kinase has differential activity in cancerous relative to normal lung, and this difference might partly sustain the synthesis of serine. Relative to normal lung, cancer-

ous lung had accumulation of glyceric acid (p-value: **4.002E-03**) and depletion of 3-phosphoglyceric acid (p-value: **2.599E-03**) (**Figure 5F, Table 6**). 3-Phosphoglyceric acid is the product of dephosphorylation of 1,3-diphosphoglyceric acid by the enzyme Phosphoglycerate kinase in the Glycolysis metabolic pathway. 3-Phosphoglyceric acid is an allosteric inhibitor of 6-Phosphogluconate dehydrogenase [65], which has a role in the Pentose Phosphate metabolic pathway [66]. 3-Phosphoglyceric acid also supplies the main route for synthesis of serine through the enzyme 3-Phosphoglycerate dehydrogenase [67, 8]. Conversely, glyceric acid is a major product of the degradation of serine [67]. Hence, the enzyme Glycerate kinase in this cluster occupies an intersection of dynamic metabolic processes that shift to sustain cancerous metabolism [65, 66, 67, 68]. The original publication of this study acknowledged accumulation of metabolites in the Pentose Phosphate Pathway but did not emphasize Glycerate kinase as a candidate enzyme of interest [49]. The metabolite set "Glycine and Serine Metabolism" most closely represents this cluster, and this set ranked 38th in metabolite set enrichment analysis.

Bisphosphoglycerate mutase might also be more active in cancerous than normal lung to enhance oxygenation or mitigate oxidative stress in tumors. 3-Phosphoglyceric acid is also the product of dephosphorylation of 2,3-diphosphoglyceric acid by Bisphosphoglycerate mutase [68]. Within red blood cells, 2,3-diphosphoglyceric acid binds to Hemoglobin and regulates its affinity for and release of dioxygen [69]. Placental cells also produce and release 2,3-diphosphoglyceric acid to promote the exchange of dioxygen between maternal and fetal blood [69]. By increasing activity of Bisphosphoglycerate mutase, cancerous lung might divert some 1,3-diphosphoglyceric acid away from Glycolysis to produce 2,3-diphosphoglyceric acid and thereby partially remedy the hypoxia in lung tumors [70]. Even minor variations in expression of Bisphosphoglycerate mutase seem to be relevant [68], and a preliminary search of transcriptomic data sets [71] demonstrated over-expression in pancreatic [72, 73] and thyroid [74, 75] cancers. Furthermore, the Tumour suppressor protein 53 (p53)-Induced Glycolysis and Apoptosis Regulator (TIGAR) resists cancerous metabolism [76] in part by degrading 2,3-diphosphoglyceric acid [77]. In tumors, increasing activity of Bisphosphoglycerate mutase and decreasing activity of TIGAR might benefit cancerous metabolism either by enhancing dioxygen delivery or by mitigating oxidative stress [78].

Cluster 2: Spermidine synthase. Study 2, Cluster 2 (**Figure 5G**) suggests that Spermidine synthase has differential activity in cancerous relative to normal lung, and this difference might allow proximal ornithine to supply synthesis of proline [49]. Relative to normal lung, cancerous lung had depletion of citrulline (p-value: **6.918E-04**), ornithine (p-value: **1.011E-05**), and spermidine (p-value: **2.531E-03**) (**Figure 5G, Table 6**). There was also accumulation of 5'-methylthioadenosine (p-value: **2.242E-03**) and adenine (p-value: **3.919E-04**) (**Figure 5G, Table 6**). Spermidine synthase in this cluster converts putrescine to spermidine and experiences inhibition from its product 5'-methylthioadenosine [79]. Accumulation of both 5'-methylthioadenosine and its degradation product adenine might indicate less activity in Methylthioadenosine phosphorylase and purine salvage [49]. The polyamines putrescine, spermidine, and spermine are abundant stabilizers of cell growth and survival, and many types of cancerous cells enhance synthesis of polyamines to facilitate proliferation [80, 8, 79]. The surprising depletion of spermidine in cancerous lung might be due to inhibition of Spermidine synthase by 5'-methylthioadenosine, and this decrease in activity might also allow ornithine to supply proline synthesis rather than polyamine synthesis [49]. Furthermore, inflammation of lung tissue, such as by smoking, enhances degradation of polyamines, and this response itself can favor carcinogenesis [80, 79]. The original publication of this study acknowledged accumulation of 5'-methylthioadenosine and depletion of both spermidine and ornithine [49]. It also emphasized Methylthioadenosine phosphorylase as a candidate enzyme of interest, but it did not emphasize Spermidine synthase as a candidate enzyme of interest [49]. The metabolite set "Spermidine and Spermine Biosynthesis" most closely represents this cluster, and this set ranked 33rd in metabolite set enrichment analysis.

Cluster 3: Uridine phosphorylase. Study 2, Cluster 3 (**Figure 5H**) suggests that Uridine phosphorylase has differential activity in cancerous relative to normal lung, and this difference might support the salvage of pyrimidines for proliferation. Relative to normal lung, cancerous lung had depletion of uridine (p-value: **4.385E-03**) and accumulation of both uracil (p-value: **1.057E-02**) and dihydrouracil (p-value: **1.449E-06**) (**Figure 5H, Table 6**). Uridine phosphorylase in this cluster interconverts uridine to uracil either for pyrimidine degradation or salvage [81]. Cancer cells require nucleotides for growth and proliferation [8, 9], and they must obtain these either by synthesis or salvage. Inhibition of pyrimidine synthesis by the uracil analogue 5-fluorouracil has been an effective chemotherapeutic strategy for decades [82]. The original publication of this study acknowledged accumulation of both uracil and dihydrouracil and emphasized Dihydropyrimidine dehydrogenase as a candidate enzyme of interest, but it did not emphasize Uridine phosphorylase as a candidate enzyme of interest [49]. The metabolite set "Pyrimidine Metabolism" most closely represents this cluster, and this set ranked 40th in metabolite set enrichment analysis.

Study 5: Obese versus Post-Exercise Muscle

Obesity is a major detriment to public health, and exercise is a life-style behavior that can mitigate this and other disease conditions profoundly [83]. Study 5 collected samples of muscle from 10 obese human patients both before and after 3 months of an exercise program [53]. We are unaware of a publication on this study. Surprisingly, we found clusters for Study 5, which had weak fold changes and p-values for all but a few analytes.

Cluster 1: Malic enzyme. Study 5, Cluster 1 (**Figure 5R**) suggests that Malic enzyme has differential activity in obese muscle relative to muscle after exercise, and this difference might balance metabolism in response to slight hypoxia. Relative to muscle in obese patients before exercise, muscle in obese patients after exercise had slight accumulation of fumaric acid (p-value: **5.084E-02**) and slight depletion of pyruvate (p-value: **5.513E-02**) (**Figure 5R, Table 6**). These metabolites both participate in reactions with L-malic acid, which did not change appreciably (p-value: **8.337E-01**). Malic enzyme in this cluster converts malic acid to pyruvic acid, with various isozymes in the Cytosol and Mitochondrion. Malic enzyme cooperates with Pyruvate dehydrogenase and the Malate-Aspartate Shuttle as a metabolic bridge between Glycolysis in the Cytosol and the Citric Acid Cycle in the Mitochondrion. This bridge balances anapleurotic demands for materials and also balances reductive and oxidative nicotinamide adenine dinucleotides for both anabolic and catabolic processes [84, 85, 86]. Interestingly, fumaric acid allosterically activates Malic enzyme in the Mitochondrion [87]. During exercise, mus-

cles might be slightly hypoxic, causing a stall in the Citric Acid Cycle and an imbalance in reduced and oxidized nicotinamide adenine dinucleotides. Malic enzyme might help to restore balance [84, 85, 86]. The metabolite set "Citric Acid Cycle" most closely represents this cluster, and this set ranked 42nd in metabolite set enrichment analysis.

Cluster 2: Xanthine dehydrogenase. Study 5, Cluster 2 (**Figure 5S**) suggests that Xanthine dehydrogenase has differential activity in obese muscle relative to muscle after exercise, and this difference might facilitate autophagy and moderate oxidative stress. Relative to muscle in obese patients before exercise, muscle in obese patients after exercise had accumulation of uric acid (p-value: **3.688E-02**) but no appreciable change in either xanthine (p-value: **3.144E-01**) or hypoxanthine (p-value: **1.777E-01**) (**Figure 5S, Table 6**). Xanthine dehydrogenase in this cluster catalyzes multiple reactions in the degradation of purines, of which uric acid is the terminal product [88]. In skeletal muscle, exercise enhances both the production of reactive oxygen species and the rate of autophagy, and both of these metabolic responses are important for muscle's adaptation to exercise [89, 90]. Xanthine dehydrogenase is likely to have a role in autophagy, and uric acid has complex contributions to oxidative stress [88]. The metabolite set "Purine Metabolism" most closely represents this cluster, and this set ranked 66th in metabolite set enrichment analysis.

Cluster 3: Histidine decarboxylase. Study 5, Cluster 3 (**Figure 5T**) suggests that Histidine decarboxylase has differential activity in obese muscle relative to muscle after exercise, and this difference might facilitate signalling for muscle recovery. Relative to muscle in obese patients before exercise, muscle in obese patients after exercise had depletion of histidine (p-value: **4.395E-02**) but no appreciable change in 1-methylhistamine (p-value: **1.348E-01**) (**Figure 5T, Table 6**). Histamine did not change appreciably. Histidine decarboxylase catalyzes synthesis of histamine from histidine, and Histamine N-methyltransferase catalyzes its degradation to 1-methylhistamine [91]. This cluster might indicate an enhancement in synthesis of histamine and its release from the cell. In skeletal muscle, exercise promotes the synthesis of histamine, where it acts as a local paracrine signal to promote blood circulation, delivery of glucose, and inflammation [91]. The metabolite set "Histidine Metabolism" most closely represents this cluster, and this set ranked 62nd in metabolite set enrichment analysis.

Discussion

In this study, we demonstrated the applicability of human metabolic networks to metabolomic experiments in human tissues. Alternative definitions of metabolic networks emphasized different aspects of the metabolic system and enhanced their context-specific relevance. Integration of metabolomic measurements from multiple retrospective studies on the non-compartmental metabolic network without hubs gave biological context for exploratory analysis and generation of speculative hypotheses for further investigation. It is feasible for specific computational algorithms to search for metabolically-relevant clusters in these measurements, offering automatable assistance to objective analyses of big data.

We demonstrated the impact of compartmentalization and metabolite hubs on the global structure of metabolic networks. Compartments disperse the network (**Figure 3A,C, Table 3**), presumably to prevent interference from disadvantageous interactions [16]. They also establish modularity (**Figure 3A,C, Table 3**) in separate environments of chemical specialization and extensive connectivity, with relevance to metabolic regulation. Hub metabolites such as proton, water, coenzyme A, nicotinamide adenine dinucleotides, adenosine phosphates, hydrogen phosphate, and dioxygen (**Table 2, Figure 4G**) dominate the global structure of metabolic networks both in close-range and long-range connectivity (**Figure 3C,D, Table 3**). These hubs obscure much of the global modularity in the metabolic network (**Figure 3C,D, Table 3**). They also obscure the influence of other metabolites such as glutamic acid, pyruvic acid, glycine, oxoglutaric acid, and cholesterol (**Figure 4H**) that are also relevant to metabolic regulation and experiments. In some instances, it may be appropriate to simplify the representations of specific hub metabolites in the metabolic network. Accurate representations of these hubs are important to observe the relevant structure of metabolic networks. These influences of compartmentalization and hubs are relevant since properties such as shortest paths and modularity directly influence the potential for detection of meaningful clusters in measurements.

Alternative definitions of metabolic networks and alternative integration of measurements might enhance resolution and accuracy in analyses. It is important to account for hubs in metabolic networks. As hubs dominate these networks, we omitted them to avoid their tendency to obscure other metabolites. An alternative strategy would be to represent the relevance of each metabolite in each reaction by the weights of their links. Whereas all links in our networks are equivalent, weights for reactions' links might represent the metabolically significant conversion of chemical mass, thereby diminishing interference of hubs. Link weights might also represent metabolic fluxes; however, kinetic rates of metabolic reactions are extremely variable and would require measurements of specific tissues and metabolic conditions for each experiment. It is also important to coordinate the compartmental context of measurements to the metabolic network. Replication of non-compartmental measurements for integration on a compartmental network is likely to introduce artifacts. An alternative strategy would be to integrate non-compartmental measurements on a compartmental network by estimating proportional sizes of metabolite pools in each cellular compartment. These alternative representations of hubs and compartments in metabolic networks might improve the relevance of network analyses such as mean path length, mean cluster coefficient, and small-world coefficient as well as influential ranks of metabolites.

We also demonstrated a strategy to detect clusters in metabolomic measurements on metabolic networks. Our strategy specifically searched for clusters of few reactions with patterns of accumulation and depletion in proximal metabolites (**Figure 5, Table 6**). This strategy identified individual genes, transcripts, and proteins that were candidates for differential regulation between experimental conditions (**Table 7**). Several of the clusters we identified were novel even after comparison to previous publications of their studies [47, 49, 51]. Interestingly, several of these clusters occupied intersections between major metabolic processes and between separate intracellular compartments (**Figure 5, Table 7**). While we used a general algorithm [54] to search for our initial clusters, a more specific algorithm might reasonably automate cluster detection and quantify confidence in each cluster [18]. A more specific algorithm might replicate our analysis by detecting clusters both by enrichment of p-values and by prioritizing clusters with both accumulation and depletion of analytes. Also, alternative search strategies might excel

at finding broader, more systemic changes, such as patterns of depletion in similar categories of metabolites, or accumulation of many metabolites within a single compartment.

Our own analyses of metabolomic measurements reiterated the advantages of network strategies over set strategies [19]. Cluster detection identified potential biological differences at a resolution of individual chemical reactions (**Table 7**). This strategy was sensitive for even subtle differences in measurements. Metabolite set enrichment analysis in MetaboAnalyst [46] offered a convenient, preliminary strategy to associate measurements to biological context. We found that a simple interpretation of results from this set enrichment analysis risked over-interpretation from a few analytes, with risk for artifacts and false-positives (**Table 5**). Cluster analysis exposed trends in a greater diversity of analytes (**Figure 5, Table 6**). Most of our clusters of interest did not occur in the top 10 hits from metabolite set enrichment analysis (**Table 5, Table 6, Table 7**).

Potential implications

Web applications enhance the accessibility of tools for biomedical data analysis. Web applications do not require installation of custom software packages, and the web document supports versatile, interactive graphical interfaces. Simple web applications also can be independent of servers, which require more development and maintenance. Our web application DyMetaboNet demonstrates some of these advantages.

Systemic biological models offer computer-readable context for metabolic experiments and have potential to develop further to integrate multiple dimensions of biology. Our own work here emphasized chemical reactions between molecular metabolites, yet biological systems also include many relevant functions and relations beyond chemical reactions that might be useful additions to metabolic models. Of particular interest are the many non-enzymatic, allosteric interactions between small-molecules and proteins [16, 92]. Also of interest are similarities between metabolites on the basis of chemical structure or other chemical properties [20]. Many different types of interactions between metabolites, genes, transcripts, and proteins constitute relevant dimensions to the biological system, and it is feasible to integrate these in multi-dimensional networks [93]. Further development of systemic biological models and definition of multi-dimensional networks might benefit the integration and analysis of measurements from multiple types of omic technologies [13, 14].

Methods

Procedures for curation, definition, and analysis of human metabolic networks

We developed the MetaboNet package [40] for reproducible curation, definition, and analysis of human metabolic networks. This package includes editable tables of parameters to customize curation and definition of these networks. Collections of scripts in the Python programming language automate these procedures. MetaboNet employs functionality from the SciPy [94], NumPy [95], NetworkX [96], MatPlotLib [97, 98], and WordCloud [99] packages.

MetaboNet requires sources of information from the Recon 2M.2 model of human metabolism [1], version 4.0 of HMDB [3], and metabolomic measurements from studies in the Metabolomics Workbench [39]. MetaboNet produces exports for integration and further analysis in MetaNetX [2], DyMetaboNet [42], NetworkX [96], Cytoscape [43], and MetaboAnalyst [46]. MetaboNet's README [40] gives more information about installation, customization, and execution of these procedures. MetaboNet is available on GitHub under the GNU General Public License [40].

Curation and adaptation of human metabolic model

We accessed the latest model of human metabolism (**Table 1**). We accessed information for the Recon 2M.2 model of human metabolism [1] from file "Recon2M.2_MNX_Entrez_Gene.xml" (14.2 Megabytes) in record "583326" of the Zenodo repository [100]. The format of this file is consistent with level 2 and version 4 of the Systems Biology Markup Language (SBML) [38], a specification of the Extensible Markup Language (XML). This version of the Recon 2M.2 model uses derivatives of identifiers and names for metabolites from the MetaNetX [2] name space and references records in Entrez Gene [5] for specific genes relevant to reactions.

We used the tools and repository of MetaNetX [2] and version 3.0 of the MNXref namespace to check for consistency and quality and to standardize the identifiers and names of metabolites and reactions. To facilitate integration with MetaNetX, we edited content of the original file for Recon 2M.2 in SBML format. We changed identifiers of metabolites to remove unnecessary prefixes and change the designation of the boundary compartment. We also changed identifiers or names of 104 metabolites and 3 compartments to correct errors and improve mapping to the MetaNetX name space. We imported this new version of Recon 2M.2 to MetaNetX, which matched information about reactions, metabolites, and compartments to its own records. Whereas Recon 2M.2 includes distinct entries for compartmental instances of metabolites, MetaNetX [2] consolidates information for chemically-identical metabolites. After reconciliation and integration to MetaNetX, we exported consensus, standard information about reactions, enzymes, metabolites, and compartments in text tables with tab delimiters. We derived our own version of the metabolic model from this information (**Table 1**).

We curated and enhanced information about metabolites in our model of human metabolism. We made 197 custom curations to information about metabolites, especially to correct and enhance references to external databases. We accessed information for all 114100 records about metabolites in version 4.0 of HMDB [3], file "hmdb_metabolites.xml" (4.2 Gigabytes). We matched the majority of metabolites in the model to records in HMDB, and derived names from these records. Also from records in HMDB we derived references to PubChem [4]. Table 1 describes the extent of curation and coverage of references for metabolites in the model of human metabolism.

We also curated and filtered information about reactions in our model of human metabolism. We made 102 custom curations to information about reactions, especially to clarify names on the basis of their references to genes [5]. We interpreted the behavior of reactions in either chemical conversion or compartmental transport of metabolites. We then included transport reactions in processes (metabolic pathways) that span multiple compartments and include matching metabolites and compartments with the reaction. We also filtered reactions to enhance the model's relevance to our analyses. The original Recon 2M.2 model [1]

included many reactions involving the exchange of metabolites with the model's boundary and the extracellular compartment, the accumulation of biomass, and the assembly and degradation of proteins. While these reactions' are relevant to simulations of metabolic flux, they do not provide relevant context for interpretation of intracellular metabolomic measurements. We removed them from the model. Table 1 describes the extent of curation and filtration of reactions in the model of human metabolism.

We converted the information about the human metabolic model for further analysis in DyMetaboNet [42].

Definition of custom metabolic networks

We defined networks to represent human metabolism. We selected a representation as a directional, bipartite network with distinct types of nodes for reactions and metabolites (**Figure 1**). In this representation, nodes for metabolites only relate to each other through nodes for reactions, such that reactant metabolites have links to their reactions and product metabolites have links from their reactions. Reversible reactions define these links in both directions.

We defined metabolic networks to represent metabolism both with and without compartmentalization (**Figure 1A-B**). Our compartmental networks include distinct nodes to distinguish between chemically-identical metabolites and reactions that occur in separate cellular compartments. Many of these reactions do not mediate any chemical change between metabolites but instead facilitate transport of metabolites between separate compartments. Our non-compartmental representation is much more concise. We only include nodes for chemically-unique metabolites and reactions. Without compartments, many reactions are chemically-redundant, and we represent these redundant replicates by a single, consensus reaction. Also, reactions that mediate compartmental transport of metabolites are irrelevant without compartments, and we exclude these from the network.

We exert customizable criteria for reactions and metabolites to qualify for representation in the network. In our model of metabolism, reactions specify the compartments in which they occur, and they also specify metabolic processes to which they belong. Hence these compartments and processes define sets of reactions and metabolites, and the relevance of these sets depends on the context of experiments. Our procedure accommodates customizable lists of compartments and processes to apply as filters. Similarly, the relevance of individual reactions and metabolites depends on the context of experiments. Our procedure also accommodates customizable lists of reactions and metabolites to include or exclude from the network. By default, we exclude metabolite hubs from the network (**Figure 1C-D, Table 2**). To qualify for representation in the network, reactions must themselves not have designations for exclusion, and they must also belong to sets of compartments and processes that pass filters. Similarly, metabolites must participate in relevant reactions in order to be part of the network. After definition of nodes and links, we selected only the largest connected component from the network.

We converted the information about human metabolic networks for further analyses in NetworkX [96], and Cytoscape [43].

Web application for definition and visual exploration of metabolic networks

We designed and developed the DyMetaboNet web application [42] for basic definition and exploration of human metabolic networks. We implemented the application's interface in the web document and its behavior in the JavaScript programming language. We used the Data-Driven Documents (D3) [101] library for JavaScript to represent dynamic information visually. The application runs in the user's internet browser independently of any server. When the user navigates in the internet browser to the Uniform Resource Locator (URL) of DyMetaboNet's host (https://tcameronwaller.github.io/dymetabonet/), all necessary source files and code download to the user's computer, and the entire application runs locally on the user's computer. The internet browser has a firewall to contain this information from web applications and thereby protect the client's computer. DyMetaboNet imports information about metabolites, reactions, compartments, and processes that MetaboNet [40] exports in a file in JavaScript Object Notation (JSON) format. From controls in its interface, DyMetaboNet defines custom networks by a similar method to MetaboNet [42]. Dynamic queries select subnetworks of interest from these custom networks using our own custom implementations of common algorithms for proximity (breadth-first search) and paths between two or more nodes (simple shortest paths) [102]. DyMetaboNet also exports tables of information about metabolites and reactions in these networks and subnetworks.

Analysis of custom metabolic networks

We applied algorithms and metrics from graph theory to describe our metabolic networks. Bipartite networks [41] such as ours require specific constraints. Where available, we selected implementations of appropriate algorithms in version 2.2 of NetworkX [96]. Where these were unavailable, we implemented our own tools in the MetaboNet package [40]. Several algorithms calculate metrics relative only to a single bipartite set of nodes, either metabolites or reactions. We specify this type of metric by the phrase "single-mode". Furthermore, several algorithms normalize metrics by comparison to their maximal possibility for a bipartite network with directional links and with identical counts of nodes in each of its bipartite sets. We specify this normalization by the phrase "comparison to maximum" or "comparison to maxima". Other algorithms normalize metrics by comparison to their mean across multiple simulations of random bipartite networks with directional links and identical counts of nodes in each of their bipartite sets. We specify this normalization by the phrase "comparison to random".

- To measure density, we used an algorithm from NetworkX [96] that normalizes the network's actual size by comparison to maximum.
- To measure the centralities of individual nodes, we used algorithms from NetworkX [96] that calculate single-mode degree and betweenness centralities and normalize these by comparison to maximum [103]. MetaboNet [40] calculates these centralities relative to the bipartite sets of nodes for both metabolites and reactions respectively. We used these centralities further to rank metabolites by a combination of their close (degree) and long-range (betweenness) influences in the metabolic networks [44].

- To measure centralization of the entire network, we implemented our own versions of algorithms that calculate single-mode degree and betweenness centralities and normalize these by comparison to maximum [104, 103]. MetaboNet [40] calculates these centralizations relative to the bipartite sets of nodes for both metabolites and reactions respectively. We only report the values relative to metabolites (**Table 3**).
- To measure cluster coefficients of individual nodes, we used an algorithm from NetworkX [96] that calculates single-mode coefficients [105].
- To measure mean cluster coefficient of the entire network, we used an algorithm from NetworkX [96] that calculates the mean of single-mode coefficients [105]. MetaboNet [40] calculates these mean cluster coefficients relative to the bipartite sets of nodes for both metabolites and reactions respectively. We only report the values relative to metabolites (**Table 3**).
- To measure the mean path length of the entire network, we implemented our own custom version of an algorithm that calculates the mean of lengths of shortest paths between all single-mode pairs of nodes [96].
- To measure the small-world coefficient of the entire network, we adaptated the sigma coefficient [45] for a bipartite network. Our custom implementation of the sigma coefficient algorithm normalizes mean cluster coefficient and mean path length by comparison to random [96].

Processing of metabolomic measurements

We curated and processed public metabolomic measurements for general analyses. We accessed metabolomic measurements from records for projects and studies within the Metabolomics Workbench [39] (**Table 4**). From these records, we extracted information about pairs and experimental groups of samples, total identifiable and unidentifiable signals for each sample, and measurements of identifiable analytes for each sample. We selected conceptual case and control experimental groups of samples to use for dividend (numerator) and divisor (denominator) respectively (**Table 4**) in calculations of fold changes. We removed analytes with inadequate coverage of measurements. If multiple analytes represented the same chemical entity redundantly, we prioritized the analyte with the least relative variance (index of dispersion or variance-to-mean ratio) in its measurements for the control experimental group. We normalized measurements for each sample to the total sum of signals in that sample. After normalization, we calculated fold changes, base-2 logarithms of fold changes, and p-values between measurements for each analyte in samples from each experimental group. These calculations depended on whether a study's samples were in dependent pairs from the same patient. For pairs of dependent samples, we calculated the mean of base-2 logarithms of fold changes for measurements from each pair, and we calculated the p-value using a two-side t-test for dependent populations. For independent samples, we calculated the base-2 logarithm of the fold change between the means of measurements from each group, and we calculated the p-value using a two-side t-test for independent populations. Our subsequent analyses used the mean base-2 logarithm of fold change and the p-value to compare each analyte between experimental groups. We visualized these values in custom volcano plots that we implemented using version 3.0.2 of MatPlotLib [97, 98].

We integrated metabolomic measurements in metabolic networks for further analysis. Most analytes in Metabolomics Workbench [39] include references to PubChem [4], and we used these references to match analytes to metabolites in our metabolic model. We critiqued all matches between analytes and metabolites for accuracy.

Analysis of metabolomic measurements in metabolic sets

We performed metabolite set enrichment analysis using version 4.0 of MetaboAnalyst [46]. We organized metabolomic measurements in a format appropriate for export to MetaboAnalyst. For compatibility, it was necessary to prepare measurements from all studies as though samples were independent, without pairs. We specified not to use any of the normalization options in MetaboAnalyst. We tested for enrichment in MetaboAnalyst's default library of 99 metabolic sets [46], considering those with 2 or more members. For each study, we summarized the sets with the top 5 ranks by p-value (**Table 5**).

Integration and analysis of metabolomic measurements in metabolic network

We integrated metabolomic measurements from each study (**Table 4**) with our metabolic network and searched for interesting clusters. We used our non-compartmental metabolic network without hubs for analyses of metabolomic measurements. We matched analytes and measurements to metabolites by common references to PubChem [4]. We imported information about the network and measurements into version 3.7.0 of Cytoscape [43] and used version 3.2.1 of the jActiveModules application [54, 55] in Cytoscape to detect raw clusters of metabolites with enrichment in p-values. We detected these raw clusters in sets of 25 at search depths of 2 links with overlap thresholds of 0.25, 0.50, and 0.75. On nodes for metabolites in these raw clusters we represented the base-2 logarithm fold change in bidirectional color saturation. We then searched for raw clusters of 3 or fewer reactions in which the majority of metabolites had measurements, and in which proximal metabolites demonstrated both accumulation and depletion. From these raw clusters we curated final clusters of interest (**Figure 5, Table 6, Table 7**), excluding metabolites without measurements and including proximal metabolites with measurements that are biologically relevant. We curated names and confirmed accuracy of genes for all reactions in these final clusters. We also collected references to Entrez Gene [5] and UniProt [] for these reactions. We summarized measurements and information about metabolites (**Table 6**) and reactions (**Table 6**) within these clusters.

Availability of source code and requirements

Curation of human metabolic model. Definition, analysis, and export of custom metabolic networks. Processing metabolomic measurements and integration with metabolic networks.

- Project name: MetaboNet
- Project home page: https://github.com/tcameronwaller/metabonet
- Operating system(s): Platform independent
- Programming language: Python 3
- Other requirements: SciPy, NumPy, NetworkX, MatPlotLib, WordCloud

- License: GNU General Public License

Dynamic definition and visual exploration of metabolic networks.

- Project name: DyMetaboNet
- Project home page: https://github.com/tcameronwaller/dymetabonet
- Operating system(s): Platform independent
- Programming language: JavaScript
- Other requirements: D3
- License: GNU General Public License

Availability of supporting data and materials

The data sets supporting the results of this article are available in the repositories on GitHub for MetaboNet [40] and DyMetaboNet [42].

Declarations

List of abbreviations

- ABP: Adenosine 3',5'-bisphosphate
- ADP: Adenosine 5'-diphosphate
- AMP: Adenosine 5'-monophosphate
- ATP: Adenosine 5'-triphosphate
- BCAA: Branched Chain Amino Acid
- ChEBI: Chemical Entities of Biological Interest
- CMP: Cytidine 5'-monophosphate
- D3: Data-Driven Documents
- EC: Enzyme Commission
- FAD2+: Flavin adenine dinucleotide
- FADH2: Flavin adenine dinucleotide reduced
- GC: Gas chromatography
- GDP: Guanosine 5'-diphosphate
- HGNC: HUGO Gene Nomenclature Committee
- HHMI: Howard Hughes Medical Institute
- HMDB: Human Metabolome Database
- HUGO: Human Genome Organization
- JSON: JavaScript Object Notation
- KEGG: Kyoto Encyclopedia of Genes and Genomes
- LC: Liquid chromatography
- MS: Mass spectrometry
- NAD1+: Nicotinamide adenine dinucleotide
- NADH: Nicotinamide adenine dinucleotide reduced
- NADP1+: Nicotinamide adenine dinucleotide phosphate
- NADPH: Nicotinamide adenine dinucleotide phosphate reduced
- NCI: National Cancer Institute
- NIDDK: National Institute of Diabetes and Digestive and Kidney Diseases
- NMR: Nuclear Magnetic Resonance
- p53: Tumour suppressor protein 53
- RefSeq: Reference Sequence
- SBML: Systems Biology Markup Language
- TIGAR: Tumour suppressor protein 53-Induced Glycolysis and Apoptosis Regulator
- UDP: Uridine 5'-diphosphate
- URL: Uniform Resource Locator
- XML: Extensible Markup Language

Ethical Approval (optional)

Not applicable.

Consent for publication

Not applicable.

Competing Interests

The authors declare that they have no conflicting or competing interests.

Funding

T.C.W. and J.A.B. received support from the National Institute of Diabetes and Digestive and Kidney Diseases (NIDDK) Interdisciplinary Training Grant T32 Program in Computational Approaches to Diabetes and Metabolism Research, 1T32DK11096601 to Wendy W. Chapman and Simon J. Fisher. B.E.C. received support from the office of the Senior Vice President for University of Utah Health Sciences. T.C.W. and J.R. received support from the National Cancer Institute (NCI) grant CA228346 to J.R. J.R. is also an Investigator of the Howard Hughes Medical Institute (HHMI).

Author's Contributions

- **Conceptualization:** T.C.W., J.R.
- **Supervision:** T.C.W., J.A.B., B.E.C., J.R.
- **Project Administration:** T.C.W., J.A.B., B.E.C., J.R.
- **Investigation:** T.C.W.
- **Formal Analysis:** T.C.W.
- **Software:** T.C.W.
- **Methodology:** T.C.W.
- **Validation:** T.C.W.
- **Data Curation:** T.C.W.
- **Resources:** T.C.W., B.E.C., J.R.
- **Funding Acquisition:** T.C.W., J.A.B., B.E.C., J.R.
- **Writing - Original Draft Preparation:** T.C.W.
- **Writing - Review and Editing:** T.C.W., J.A.B., B.E.C., J.R.
- **Visualization:** T.C.W.

Acknowledgements

We thank Kathryn A. Waller and Alexander Lex for consultation on the visual design and development of DyMetaboNet, Sara L. Johnson for consultation on comparison of our analyses to standard practice, and Kevin G. Hicks and Alex J. Bott for consultation and perspective on metabolomic technologies and metabolic research.

Authors' information (optional)

Not applicable.

References

1. Ryu JY, Kim HU, Lee SY. Framework and resource for more than 11,000 gene-transcript-protein-reaction asso-

ciations in human metabolism. Proceedings of the National Academy of Sciences of the United States of America 2017;114(45):E9740–E9749.
2. Moretti S, Martin O, Van Du Tran T, Bridge A, Morgat A, Pagni M. MetaNetX/MNXref–reconciliation of metabolites and biochemical reactions to bring together genome-scale metabolic networks. Nucleic Acids Research 2016 Jan;44(D1):D523–526.
3. Wishart DS, Feunang YD, Marcu A, Guo AC, Liang K, Vázquez-Fresno R, et al. HMDB 4.0: the human metabolome database for 2018. Nucleic Acids Research 2018 Jan;46(D1):D608–D617.
4. Kim S, Thiessen PA, Bolton EE, Chen J, Fu G, Gindulyte A, et al. PubChem Substance and Compound databases. Nucleic Acids Research 2016 Jan;44(D1):D1202–1213.
5. Brown GR, Hem V, Katz KS, Ovetsky M, Wallin C, Ermolaeva O, et al. Gene: a gene-centered information resource at NCBI. Nucleic Acids Research 2015 Jan;43(Database issue):D36–42.
6. Bairoch A. The ENZYME database in 2000. Nucleic Acids Research 2000 Jan;28(1):304–305.
7. Artimo P, Jonnalagedda M, Arnold K, Baratin D, Csardi G, de Castro E, et al. ExPASy: SIB bioinformatics resource portal. Nucleic Acids Research 2012 Jul;40(Web Server issue):W597–603.
8. Pavlova NN, Thompson CB. The Emerging Hallmarks of Cancer Metabolism. Cell Metabolism 2016 Jan;23(1):27–47.
9. Vander Heiden MG, DeBerardinis RJ. Understanding the Intersections between Metabolism and Cancer Biology. Cell 2017;168(4):657–669.
10. Hotamisligil GS. Inflammation, metaflammation and immunometabolic disorders. Nature 2017;542(7640):177–185.
11. Hu JX, Thomas CE, Brunak S. Network biology concepts in complex disease comorbidities. Nature Reviews Genetics 2016;17(10):615–629.
12. Kaushik AK, DeBerardinis RJ. Applications of metabolomics to study cancer metabolism. Biochimica Et Biophysica Acta Reviews on Cancer 2018 Aug;1870(1):2–14.
13. Ritchie MD, Holzinger ER, Li R, Pendergrass SA, Kim D. Methods of integrating data to uncover genotype-phenotype interactions. Nature Reviews Genetics 2015 Feb;16(2):85–97.
14. Karczewski KJ, Snyder MP. Integrative omics for health and disease. Nature Reviews Genetics 2018 May;19(5):299–310.
15. Barabási AL, Oltvai ZN. Network biology: understanding the cell's functional organization. Nature Reviews Genetics 2004 Feb;5(2):101–113.
16. Alam MT, Olin-Sandoval V, Stincone A, Keller MA, Zelezniak A, Luisi BF, et al. The self-inhibitory nature of metabolic networks and its alleviation through compartmentalization. Nature Communications 2017;8:16018.
17. Gottschling DE, Nyström T. The Upsides and Downsides of Organelle Interconnectivity. Cell 2017;169(1):24–34.
18. Mitra K, Carvunis AR, Ramesh SK, Ideker T. Integrative approaches for finding modular structure in biological networks. Nature Reviews Genetics 2013 Oct;14(10):719–732.
19. García-Campos MA, Espinal-Enríquez J, Hernández-Lemus E. Pathway Analysis: State of the Art. Frontiers in Physiology 2015;6:383.
20. Barupal DK, Fan S, Fiehn O. Integrating bioinformatics approaches for a comprehensive interpretation of metabolomics datasets. Current Opinion in Biotechnology 2018 Dec;54:1–9.
21. Le Novère N. Quantitative and logic modelling of molecular and gene networks. Nature Reviews Genetics 2015 Mar;16(3):146–158.
22. O'Brien EJ, Monk JM, Palsson BO. Using Genome-scale Models to Predict Biological Capabilities. Cell 2015 May;161(5):971–987.
23. Hastings J, Owen G, Dekker A, Ennis M, Kale N, Muthukrishnan V, et al. ChEBI in 2016: Improved services and an expanding collection of metabolites. Nucleic Acids Research 2016 Jan;44(D1):D1214–1219.
24. Kanehisa M, Furumichi M, Tanabe M, Sato Y, Morishima K. KEGG: new perspectives on genomes, pathways, diseases and drugs. Nucleic Acids Research 2017;45(D1):D353–D361.
25. Caspi R, Billington R, Fulcher CA, Keseler IM, Kothari A, Krummenacker M, et al. The MetaCyc database of metabolic pathways and enzymes. Nucleic Acids Research 2018 Jan;46(D1):D633–D639.
26. Fabregat A, Jupe S, Matthews L, Sidiropoulos K, Gillespie M, Garapati P, et al. The Reactome Pathway Knowledgebase. Nucleic Acids Research 2018 Jan;46(D1):D649–D655.
27. Braschi B, Denny P, Gray K, Jones T, Seal R, Tweedie S, et al. Genenames.org: the HGNC and VGNC resources in 2019. Nucleic Acids Research 2018 Oct;.
28. O'Leary NA, Wright MW, Brister JR, Ciufo S, Haddad D, McVeigh R, et al. Reference sequence (RefSeq) database at NCBI: current status, taxonomic expansion, and functional annotation. Nucleic Acids Research 2016 Jan;44(D1):D733–745.
29. Zerbino DR, Achuthan P, Akanni W, Amode MR, Barrell D, Bhai J, et al. Ensembl 2018. Nucleic Acids Research 2018 Jan;46(D1):D754–D761.
30. UniProt Consortium T. UniProt: the universal protein knowledgebase. Nucleic Acids Research 2018 Mar;46(5):2699.
31. Duarte NC, Becker SA, Jamshidi N, Thiele I, Mo ML, Vo TD, et al. Global reconstruction of the human metabolic network based on genomic and bibliomic data. Proceedings of the National Academy of Sciences of the United States of America 2007 Feb;104(6):1777–1782.
32. Hao T, Ma HW, Zhao XM, Goryanin I. Compartmentalization of the Edinburgh Human Metabolic Network. BMC bioinformatics 2010 Jul;11:393.
33. Thiele I, Swainston N, Fleming RMT, Hoppe A, Sahoo S, Aurich MK, et al. A community-driven global reconstruction of human metabolism. Nature Biotechnology 2013 May;31(5):419–425.
34. Mardinoglu A, Agren R, Kampf C, Asplund A, Uhlen M, Nielsen J. Genome-scale metabolic modelling of hepatocytes reveals serine deficiency in patients with non-alcoholic fatty liver disease. Nature Communications 2014;5:3083.
35. Swainston N, Smallbone K, Hefzi H, Dobson PD, Brewer J, Hanscho M, et al. Recon 2.2: from reconstruction to model of human metabolism. Metabolomics: Official Journal of the Metabolomic Society 2016;12:109.
36. King ZA, Lu J, Dräger A, Miller P, Federowicz S, Lerman JA, et al. BiGG Models: A platform for integrating, standardizing and sharing genome-scale models. Nucleic Acids Research 2016 Jan;44(D1):D515–522.
37. Cottret L, Frainay C, Chazalviel M, Cabanettes F, Gloaguen Y, Camenen E, et al. MetExplore: collaborative edition and exploration of metabolic networks. Nucleic Acids Research 2018 Jul;46(W1):W495–W502.

38. Hucka M, Finney A, Sauro HM, Bolouri H, Doyle JC, Kitano H, et al. The systems biology markup language (SBML): a medium for representation and exchange of biochemical network models. Bioinformatics (Oxford, England) 2003 Mar;19(4):524–531.
39. Sud M, Fahy E, Cotter D, Azam K, Vadivelu I, Burant C, et al. Metabolomics Workbench: An international repository for metabolomics data and metadata, metabolite standards, protocols, tutorials and training, and analysis tools. Nucleic Acids Research 2016 Jan;44(D1):D463–470.
40. Waller TC, MetaboNet. GitHub; 2018. https://github.com/tcameronwaller/metabonet.
41. Pavlopoulos GA, Kontou PI, Pavlopoulou A, Bouyioukos C, Markou E, Bagos PG. Bipartite graphs in systems biology and medicine: a survey of methods and applications. GigaScience 2018;7(4):1–31.
42. Waller TC, DyMetaboNet. GitHub; 2018. https://github.com/tcameronwaller/dymetabonet.
43. Shannon P, Markiel A, Ozier O, Baliga NS, Wang JT, Ramage D, et al. Cytoscape: a software environment for integrated models of biomolecular interaction networks. Genome Research 2003 Nov;13(11):2498–2504.
44. Liao H, Mariani MS, Medo M, Zhang YC, Zhou MY. Ranking in evolving complex networks. Physics Reports 2017 May;689:1–54. https://linkinghub.elsevier.com/retrieve/pii/S0370157317300935.
45. Humphries MD, Gurney K. Network 'small-world-ness': a quantitative method for determining canonical network equivalence. PloS One 2008 Apr;3(4):e0002051.
46. Chong J, Soufan O, Li C, Caraus I, Li S, Bourque G, et al. MetaboAnalyst 4.0: towards more transparent and integrative metabolomics analysis. Nucleic Acids Research 2018 Jul;46(W1):W486–W494.
47. Liesenfeld DB, Grapov D, Fahrmann JF, Salou M, Scherer D, Toth R, et al. Metabolomics and transcriptomics identify pathway differences between visceral and subcutaneous adipose tissue in colorectal cancer patients: the ColoCare study. The American Journal of Clinical Nutrition 2015 Aug;102(2):433–443.
48. Fiehn O, Project PR000058, Study ST000061. Metabolomics Workbench; 2014. https://doi.org/10.21228/M80018.
49. Wikoff WR, Grapov D, Fahrmann JF, DeFelice B, Rom WN, Pass HI, et al. Metabolomic markers of altered nucleotide metabolism in early stage adenocarcinoma. Cancer Prevention Research (Philadelphia, Pa) 2015 May;8(5):410–418.
50. Fiehn O, Project PR000305, Study ST000390. Metabolomics Workbench; 2010. https://doi.org/10.21228/M8PG66.
51. Bruinsma BG, Sridharan GV, Weeder PD, Avruch JH, Saeidi N, Özer S, et al. Metabolic profiling during ex vivo machine perfusion of the human liver. Scientific Reports 2016 Mar;6:22415.
52. Fiehn O, Project PR000322, Study ST000412. Metabolomics Workbench; 2016. https://doi.org/10.21228/M8V312.
53. Kachman M, Horowitz J, Project PR000599, Study ST000842. Metabolomics Workbench; 2017. https://doi.org/10.21228/M87Q3F.
54. Ideker T, Ozier O, Schwikowski B, Siegel AF. Discovering regulatory and signalling circuits in molecular interaction networks. Bioinformatics (Oxford, England) 2002;18 Suppl 1:S233–240.
55. Saito R, Smoot ME, Ono K, Ruscheinski J, Wang PL, Lotia S, et al. A travel guide to Cytoscape plugins. Nature Methods 2012 Nov;9(11):1069–1076.
56. Lynes MD, Tseng YH. Deciphering adipose tissue heterogeneity. Annals of the New York Academy of Sciences 2018 Jan;1411(1):5–20.
57. Ibrahim MM. Subcutaneous and visceral adipose tissue: structural and functional differences. Obesity Reviews: An Official Journal of the International Association for the Study of Obesity 2010 Jan;11(1):11–18.
58. Xourgia E, Papazafiropoulou A, Melidonis A. Effects of antidiabetic drugs on epicardial fat. World Journal of Diabetes 2018 Sep;9(9):141–148.
59. Sugimoto S, Nakajima H, Kosaka K, Hosoi H. Review: Miglitol has potential as a therapeutic drug against obesity. Nutrition & Metabolism 2015;12:51.
60. Lynch CJ, Adams SH. Branched-chain amino acids in metabolic signalling and insulin resistance. Nature Reviews Endocrinology 2014 Dec;10(12):723–736.
61. Zhao X, Han Q, Liu Y, Sun C, Gang X, Wang G. The Relationship between Branched-Chain Amino Acid Related Metabolomic Signature and Insulin Resistance: A Systematic Review. Journal of Diabetes Research 2016;2016:2794591.
62. Arany Z, Neinast M. Branched Chain Amino Acids in Metabolic Disease. Current Diabetes Reports 2018 Aug;18(10):76.
63. Lee PL, Jung SM, Guertin DA. The Complex Roles of Mechanistic Target of Rapamycin in Adipocytes and Beyond. Trends in endocrinology and metabolism: TEM 2017;28(5):319–339.
64. Caldwell RW, Rodriguez PC, Toque HA, Narayanan SP, Caldwell RB. Arginase: A Multifaceted Enzyme Important in Health and Disease. Physiological Reviews 2018;98(2):641–665.
65. Hitosugi T, Zhou L, Elf S, Fan J, Kang HB, Seo JH, et al. Phosphoglycerate mutase 1 coordinates glycolysis and biosynthesis to promote tumor growth. Cancer Cell 2012 Nov;22(5):585–600.
66. Lin R, Elf S, Shan C, Kang HB, Ji Q, Zhou L, et al. 6-Phosphogluconate dehydrogenase links oxidative PPP, lipogenesis and tumour growth by inhibiting LKB1-AMPK signalling. Nature Cell Biology 2015 Nov;17(11):1484–1496.
67. Mattaini KR, Sullivan MR, Vander Heiden MG. The importance of serine metabolism in cancer. The Journal of Cell Biology 2016;214(3):249–257.
68. Oslund RC, Su X, Haugbro M, Kee JM, Esposito M, David Y, et al. Bisphosphoglycerate mutase controls serine pathway flux via 3-phosphoglycerate. Nature Chemical Biology 2017 Oct;13(10):1081–1087.
69. Pritlove DC, Gu M, Boyd CaR, Randeva HS, Vatish M. Novel placental expression of 2,3-bisphosphoglycerate mutase. Placenta 2006 Aug;27(8):924–927.
70. Salem A, Asselin MC, Reymen B, Jackson A, Lambin P, West CML, et al. Targeting Hypoxia to Improve Non-Small Cell Lung Cancer Outcome. Journal of the National Cancer Institute 2018 Jan;110(1).
71. Barrett T, Wilhite SE, Ledoux P, Evangelista C, Kim IF, Tomashevsky M, et al. NCBI GEO: archive for functional genomics data sets–update. Nucleic Acids Research 2013 Jan;41(Database issue):D991–995.
72. Wang L, Series GSE16515, Data Set GDS4102, Profile 78838150. Gene Expression Omnibus (GEO); 2009. https://www.ncbi.nlm.nih.gov/geoprofiles/78838150.
73. Pei H, Li L, Fridley BL, Jenkins GD, Kalari KR, Lingle W, et al. FKBP51 affects cancer cell response to chemotherapy by neg-

atively regulating Akt. Cancer Cell 2009 Sep;16(3):259–266.
74. Reyes I, Series GSE3678, Data Set GDS1732, Profile 18862150. Gene Expression Omnibus (GEO); 2005. https://www.ncbi.nlm.nih.gov/geoprofiles/18862150.
75. Kummer NT, Nowicki TS, Azzi JP, Reyes I, Iacob C, Xie S, et al. Arachidonate 5 lipoxygenase expression in papillary thyroid carcinoma promotes invasion via MMP-9 induction. Journal of Cellular Biochemistry 2012 Jun;113(6):1998–2008.
76. Flöter J, Kaymak I, Schulze A. Regulation of Metabolic Activity by p53. Metabolites 2017 May;7(2).
77. Gerin I, Noël G, Bolsée J, Haumont O, Van Schaftingen E, Bommer GT. Identification of TP53-induced glycolysis and apoptosis regulator (TIGAR) as the phosphoglycolate-independent 2,3-bisphosphoglycerate phosphatase. The Biochemical Journal 2014 Mar;458(3):439–448.
78. Bolaños JP. TIGAR's promiscuity. The Biochemical Journal 2014 Mar;458(3):e5–7.
79. Casero RA, Murray Stewart T, Pegg AE. Polyamine metabolism and cancer: treatments, challenges and opportunities. Nature Reviews Cancer 2018 Nov;18(11):681–695.
80. Murray-Stewart TR, Woster PM, Casero RA. Targeting polyamine metabolism for cancer therapy and prevention. The Biochemical Journal 2016;473(19):2937–2953.
81. Garavito MF, Narváez-Ortiz HY, Zimmermann BH. Pyrimidine Metabolism: Dynamic and Versatile Pathways in Pathogens and Cellular Development. Journal of Genetics and Genomics = Yi Chuan Xue Bao 2015 May;42(5):195–205.
82. Luengo A, Gui DY, Vander Heiden MG. Targeting Metabolism for Cancer Therapy. Cell Chemical Biology 2017 Sep;24(9):1161–1180.
83. Zafar U, Khaliq S, Ahmad HU, Manzoor S, Lone KP. Metabolic syndrome: an update on diagnostic criteria, pathogenesis, and genetic links. Hormones (Athens, Greece) 2018 Sep;17(3):299–313.
84. Nielsen TT, Støttrup NB, Løfgren B, Bøtker HE. Metabolic fingerprint of ischaemic cardioprotection: importance of the malate-aspartate shuttle. Cardiovascular Research 2011 Aug;91(3):382–391.
85. Doenst T, Nguyen TD, Abel ED. Cardiac metabolism in heart failure: implications beyond ATP production. Circulation Research 2013 Aug;113(6):709–724.
86. Chicco AJ, Le CH, Gnaiger E, Dreyer HC, Muyskens JB, D'Alessandro A, et al. Adaptive remodeling of skeletal muscle energy metabolism in high-altitude hypoxia: Lessons from AltitudeOmics. The Journal of Biological Chemistry 2018 May;293(18):6659–6671.
87. Yang Z, Lanks CW, Tong L. Molecular mechanism for the regulation of human mitochondrial NAD(P)+-dependent malic enzyme by ATP and fumarate. Structure (London, England: 1993) 2002 Jul;10(7):951–960.
88. Kang DH, Ha SK. Uric Acid Puzzle: Dual Role as Anti-oxidantand Pro-oxidant. Electrolyte & blood pressure: E & BP 2014 Jun;12(1):1–6.
89. Ferraro E, Giammarioli AM, Chiandotto S, Spoletini I, Rosano G. Exercise-induced skeletal muscle remodeling and metabolic adaptation: redox signaling and role of autophagy. Antioxidants & Redox Signaling 2014 Jul;21(1):154–176.
90. Trewin AJ, Berry BJ, Wojtovich AP. Exercise and Mitochondrial Dynamics: Keeping in Shape with ROS and AMPK. Antioxidants (Basel, Switzerland) 2018 Jan;7(1).
91. Luttrell MJ, Halliwill JR. The Intriguing Role of Histamine in Exercise Responses. Exercise and Sport Sciences Reviews 2017;45(1):16–23.
92. Reznik E, Christodoulou D, Goldford JE, Briars E, Sauer U, Segrè D, et al. Genome-Scale Architecture of Small Molecule Regulatory Networks and the Fundamental Trade-Off between Regulation and Enzymatic Activity. Cell Reports 2017 Sep;20(11):2666–2677.
93. Haas R, Zelezniak A, Iacovacci J, Kamrad S, Townsend S, Ralser M. Designing and interpreting 'multi-omic' experiments that may change our understanding of biology. Current Opinion in Systems Biology 2017 Dec;6:37–45. https://linkinghub.elsevier.com/retrieve/pii/S2452310017300835.
94. Jones E, Oliphant TE, Peterson P, SciPy: Open source scientific tools for Python; 2001. http://www.scipy.org/.
95. Oliphant TE. Guide to NumPy; 2015. OCLC: 1030608394.
96. Hagberg AA, Schult DA, Swart PJ. Exploring Network Structure, Dynamics, and Function using NetworkX. In: Varoquaux G, Vaught T, Millman J, editors. Proceedings of the 7th Python in Science Conference Pasadena, California; 2008. p. 11–15. http://conference.scipy.org/proceedings/SciPy2008/paper_2/.
97. Hunter JD. Matplotlib: A 2D Graphics Environment. Computing in Science & Engineering 2007;9(3):90–95. http://ieeexplore.ieee.org/document/4160265/.
98. Caswell TA, Droettboom M, Hunter J, Firing E, Lee A, Stansby D, et al., Matplotlib/Matplotlib V3.0.2. Zenodo; 2018. https://zenodo.org/record/1482099.
99. Mueller A, WordCloud for Python; 2018. https://amueller.github.io/word_cloud/.
100. Ryu JY, Kim HU, Lee SY, Framework and resource for more than 11,000 gene-transcript-protein-reaction associations in human metabolism. Zenodo; 2017. https://zenodo.org/record/583326.
101. Bostock M, Ogievetsky V, Heer J. D^3: Data-Driven Documents. IEEE transactions on visualization and computer graphics 2011 Dec;17(12):2301–2309.
102. Yen JY. Finding the K Shortest Loopless Paths in a Network. Management Science 1971 Jul;17(11):712–716. http://pubsonline.informs.org/doi/abs/10.1287/mnsc.17.11.712.
103. Borgatti SP, Halgin DS, Scott J, Carrington PJ, editors, Analyzing Affiliation Networks. 1 Oliver's Yard, 55 City Road, London EC1Y 1SP United Kingdom: SAGE Publications Ltd; 2014. http://methods.sagepub.com/book/the-sage-handbook-of-social-network-analysis/n28.xml.
104. Borgatti SP, Everett MG. Network analysis of 2-mode data. Social Networks 1997 Aug;19(3):243–269. http://linkinghub.elsevier.com/retrieve/pii/S0378873396003012.
105. Latapy M, Magnien C, Vecchio ND. Basic notions for the analysis of large two-mode networks. Social Networks 2008 Jan;30(1):31–48. http://linkinghub.elsevier.com/retrieve/pii/S0378873307000494.
