# Supplementary material for: Compartment and hub definitions tune metabolic networks for metabolomic interpretations
Source: Gigascience. 2020 Jan 23;9(1):giz137. doi: 10.1093/gigascience/giz137 (PMC6977586; doi:10.1093/gigascience/giz137)
Supplement: giz137_GIGA-D-18-00489_Revision_1 [file giz137_giga-d-18-00489_revision_1.pdf]

GigaScience

Compartment and Hub Definitions Tune Metabolic Networks for Metabolomic Interpretations

--Manuscript Draft--

Manuscript Number:	GIGA-D-18-00489R1
Full Title:	Compartment and Hub Definitions Tune Metabolic Networks for Metabolomic Interpretations
Article Type:	Research
Funding Information:	National Institute of Diabetes and Digestive and Kidney Diseases (1T32DK11096601) — Mr. Thomas Cameron Waller, Mr. Jordan Alexander Berg<br>National Cancer Institute (CA228346) — Dr. Jared Paul Rutter
Abstract:	Background: Metabolic networks represent all chemical reactions that occur between molecular metabolites in an organism's cells. They offer biological context in which to integrate, analyze, and interpret "-omic" measurements, but their large scale and extensive connectivity present unique challenges. While it is practical to simplify these networks by placing constraints on compartments and hubs, it is unclear how these simplifications alter the structure of metabolic networks and the interpretation of metabolomic experiments.<br>Results: We curated and adapted the latest systemic model of human metabolism and developed customizable tools to define metabolic networks with and without compartmentalization in subcellular organelles and with or without inclusion of prolific metabolite hubs. Compartmentalization made networks larger, less dense, and more modular; whereas hubs made networks larger, more dense, and less modular. When present, these hubs also dominated shortest paths in the network, yet their exclusion exposed the subtler prominence of other metabolites that are typically more relevant to metabolomic experiments. We applied the non-compartmental network without metabolite hubs in a retrospective, exploratory analysis of metabolomic measurements from five studies on human tissues. Network clusters identified individual reactions that might experience differential regulation between experimental conditions, several of which were not apparent in the original publications.<br>Conclusions: Exclusion of specific metabolite hubs exposes modularity in both compartmental and non-compartmental metabolic networks, improving detection of relevant clusters in "-omic" measurements. Better computational detection of metabolic network clusters in large data sets has potential to identify differential regulation of individual genes, transcripts, and proteins.
Corresponding Author:	Thomas Cameron Waller, B.S.<br>University of Utah<br>Salt Lake City, Utah UNITED STATES
Corresponding Author Secondary Information:	
Corresponding Author's Institution:	University of Utah
Corresponding Author's Secondary Institution:	
First Author:	Thomas Cameron Waller, B.S.
First Author Secondary Information:	
Order of Authors:	Thomas Cameron Waller, B.S.
	Jordan Alexander Berg, B.S.
	Brian Earl Chapman, Ph.D.
	Jared Paul Rutter, Ph.D.
Order of Authors Secondary Information:	

Powered by Editorial Manager® and ProduXion Manager® from Aries Systems Corporation

Response to Reviewers:	Please refer to file "waller_response.pdf" for letter response to reviewer comments.
Additional Information:	
Question	Response
Are you submitting this manuscript to a special series or article collection?	No
**Experimental design and statistics**<br><br>Full details of the experimental design and statistical methods used should be given in the Methods section, as detailed in our Minimum Standards Reporting Checklist. Information essential to interpreting the data presented should be made available in the figure legends.<br><br>Have you included all the information requested in your manuscript?	Yes
**Resources**<br><br>A description of all resources used, including antibodies, cell lines, animals and software tools, with enough information to allow them to be uniquely identified, should be included in the Methods section. Authors are strongly encouraged to cite Research Resource Identifiers (RRIDs) for antibodies, model organisms and tools, where possible.<br><br>Have you included the information requested as detailed in our Minimum Standards Reporting Checklist?	Yes
**Availability of data and materials**<br><br>All datasets and code on which the conclusions of the paper rely must be either included in your submission or deposited in publicly available repositories (where available and ethically appropriate), referencing such data using a unique identifier in the references and in the "Availability of Data and Materials" section of your manuscript.	Yes

Powered by Editorial Manager® and ProduXion Manager® from Aries Systems Corporation

Have you have met the above requirement as detailed in our Minimum Standards Reporting Checklist?	

Powered by Editorial Manager® and ProduXion Manager® from Aries Systems Corporation

Manuscript with DOI
Click here to view linked References
Click here to access/download;Manuscript;GIGA-D-18-00489_Waller R1 with DOI.pdf

OXFORD (GIGA)^n SCIENCE

GigaScience, 2017, 1–19

doi: xx.xxxx/xxxx
Manuscript in Preparation
Research

RESEARCH

Compartment and Hub Definitions Tune Metabolic Networks for Metabolomic Interpretations

T. Cameron Waller[1,2,*], Jordan A. Berg[2], Alexander Lex[3,4], Brian E. Chapman[5,6] and Jared Rutter[2,7,†]

[1]Department of Medicine, University of California San Diego, La Jolla, California, United States of America and [2]Department of Biochemistry, University of Utah, Salt Lake City, Utah, United States of America and [3]School of Computing, University of Utah, Salt Lake City, Utah, United States of America and [4]Scientific Computing and Imaging Institute, University of Utah, Salt Lake City, Utah, United States of America and [5]Department of Radiology and Imaging Sciences, University of Utah, Salt Lake City, Utah, United States of America and [6]Department of Biomedical Informatics, University of Utah, Salt Lake City, Utah, United States of America and [7]Howard Hughes Medical Institute, University of Utah, Salt Lake City, Utah, United States of America

*Corresponding author: tcwaller@ucsd.edu
†Corresponding author: rutter@biochem.utah.edu

Abstract

Background: Metabolic networks represent all chemical reactions that occur between molecular metabolites in an organism's cells. They offer biological context in which to integrate, analyze, and interpret "-omic" measurements, but their large scale and extensive connectivity present unique challenges. While it is practical to simplify these networks by placing constraints on compartments and hubs, it is unclear how these simplifications alter the structure of metabolic networks and the interpretation of metabolomic experiments.
Results: We curated and adapted the latest systemic model of human metabolism and developed customizable tools to define metabolic networks with and without compartmentalization in subcellular organelles and with or without inclusion of prolific metabolite hubs. Compartmentalization made networks larger, less dense, and more modular; whereas hubs made networks larger, more dense, and less modular. When present, these hubs also dominated shortest paths in the network, yet their exclusion exposed the subtler prominence of other metabolites that are typically more relevant to metabolomic experiments. We applied the non-compartmental network without metabolite hubs in a retrospective, exploratory analysis of metabolomic measurements from five studies on human tissues. Network clusters identified individual reactions that might experience differential regulation between experimental conditions, several of which were not apparent in the original publications.
Conclusions: Exclusion of specific metabolite hubs exposes modularity in both compartmental and non-compartmental metabolic networks, improving detection of relevant clusters in "-omic" measurements. Better computational detection of metabolic network clusters in large data sets has potential to identify differential regulation of individual genes, transcripts, and proteins.

Key words: metabolism; metabolite; metabolomic; network

Compiled on: October 27, 2019.
Draft manuscript prepared by the author.

Background

Life is inherently unstable, and cellular metabolism acts as its vanguard, continually adapting to sustain balance. This system is complex, requiring cooperation and coordination between many types of biochemical entities [1]. Large, polymeric molecules–lipids, sugars, nucleic acids, and peptides–compose the cellular structure and machinery to propagate hereditary information; however, alone these biomacromolecues are lifeless. Genes and transcripts encode the proteins that act as enzymes to catalyze chemical reactions between smaller constituent molecules, metabolites, and these reactions both assemble biomacromolecules and supply energy to drive their functions. Eukaryotic cells further compartmentalize groups of these reactions within membrane-bound organelles, utilizing protein transporters to regulate the exchange of metabolites between environments. This dynamic interdependence constitutes metabolic regulation.

There is a growing need for strategies to investigate metabolic adaptation in human health and disease. Traditional, reductionist biology tends to conceptualize cellular metabolism as a collection of separate pathways or processes (groups of reactions) that perform their own unique functions with little interaction. However, a growing body of work has demonstrated surprising versatility in the metabolic system, especially in human diseases such as obesity, diabetes, and cancer [2, 3, 4]. Connectivity in metabolism is such that local perturbations such as mutations or post-translational modifications of individual enzymes or transporters can impose pervasive effects that blur distinctions between typical pathways and cellular compartments. It is also common for multiple perturbations to combine cooperatively and thereby aggravate complex diseases [5]. Consequently, the appropriate study of metabolic mechanisms in these diseases requires experimentation at a systemic level. Modern "-omic" technologies measure the abundance and modification of genes, transcripts, proteins, and metabolites with nearly comprehensive coverage [6]; however, there is a need for strategies to integrate this system-wide biological context with functional interpretations of these measurements [7, 8].

Biological networks are abstract projections that are useful to study these complex, real systems. Interestingly, even the global structures of molecular biological networks are informative; patterns of efficient communication between specialized modules suggest competing mechanisms of stochastic evolution and natural selection [9]. Furthermore, networks are computer-readable, semantic models; and combining this framework with rich compilations of biological knowledge can provide context for integration, analysis, and interpretation of experimental data [8]. By mapping measurements to these networks, clusters or patterns of differential measurements implicate specific types of perturbation [10]. While there has been more emphasis on networks that represent gene-gene or protein-protein interactions, metabolic networks have their own special considerations as they depict a distinct dimension of cellular biology.

As metabolic networks are abstract projections, their definitions can emphasize different aspects of the metabolic system. This versatility argues for customizability, in particular with regard to compartments and hubs. Subcellular compartmentalization within membranous organelles is an important dimension of metabolism in eukaryotic cells, yet standard metabolomic measurements on bulk samples do not discriminate between pools of metabolites in separate compartments. Hence, for the sake of analyzing and interpreting these measurements, it may or may not be reasonable to simplify cellular metabolism by ignoring compartmentalization [11]. Also, a few metabolites, such as water, dioxygen, and carbon dioxide, are especially common reactants and products in metabolic reactions, and these metabolites dominate connectivity in metabolic networks as hubs [9]. It can be practical to exclude these hub metabolites from metabolic networks in order to expose more subtle network structures [11]. It is unclear how these simplifications for compartmentalization and hubs alter the structures and properties of metabolic networks.

Here we describe alternative definitions of metabolic networks and their relevance in application to experiments. We hypothesized that alternative representations of metabolism using compartmental or non-compartmental network models with or without metabolite hubs would differentially influence the interpretations of metabolomic experiments. We also explored the potential for algorithms to detect biologically relevant clusters of metabolomic measurements on these networks. Our goal was to define these networks and describe their differences, while also providing methods and tools for future use in the community. To this end, we curated and adapted the latest systemic model of human metabolism [12]. We designed and developed a web application, DyMetaboNet [13], with a dynamic, visual interface to illustrate alternative definitions of metabolic networks. We also created a software package, MetaboNet [14], with procedures to define these networks from customizable parameters. We then compared these networks by various graph-theory metrics to understand their differences. Finally, we demonstrated the application of one network definition as biological context in a retrospective analysis of metabolomic measurements from multiple previous studies. All of our data are available in a public archive [15]. This work informs the future development of standard tools for interpretation of -omic measurements in metabolic experiments.

Data Description

Metabolic model

Systemic metabolic models summarize all chemical reactions between small-molecular metabolites that occur within an organism. Another name for these models is genome-scale metabolic reconstructions, with major applications in computational simulations to predict broader cellular growth and to resolve finer metabolic flux balance analysis through specific pathways [23, 24]. These models are also of more general utility as they integrate multiple types of functional information within computer-readable summaries [23, 24]. Information about metabolites includes common names and chemical attributes such as formula, mass, and charge. Information about reactions includes common names, directionality and reversibility, reactant and product metabolites, and compartments where they occur. Importantly, both metabolites and reactions include references to external databases that offer both supporting evidence and supplemental information. Relevant references for metabolites include the Human Metabolome Database (HMDB) [17], PubChem [18], Chemical Entities of Biological Interest (ChEBI) [25], and the Kyoto Encyclopedia of Genes and Genomes (KEGG) [26]. Relevant references for reactions include KEGG [26], MetaCyc [27], and Reactome [28]. Also relevant to reactions are references for genes, transcripts, and proteins such as Entrez Gene [19], Human Genome Organization (HUGO) Gene Nomenclature Committee (HGNC) [29], Reference Sequence (RefSeq) [30], Ensembl [31], UniProt [32], and ExPASy Enzyme Nomenclature Database (Ex-

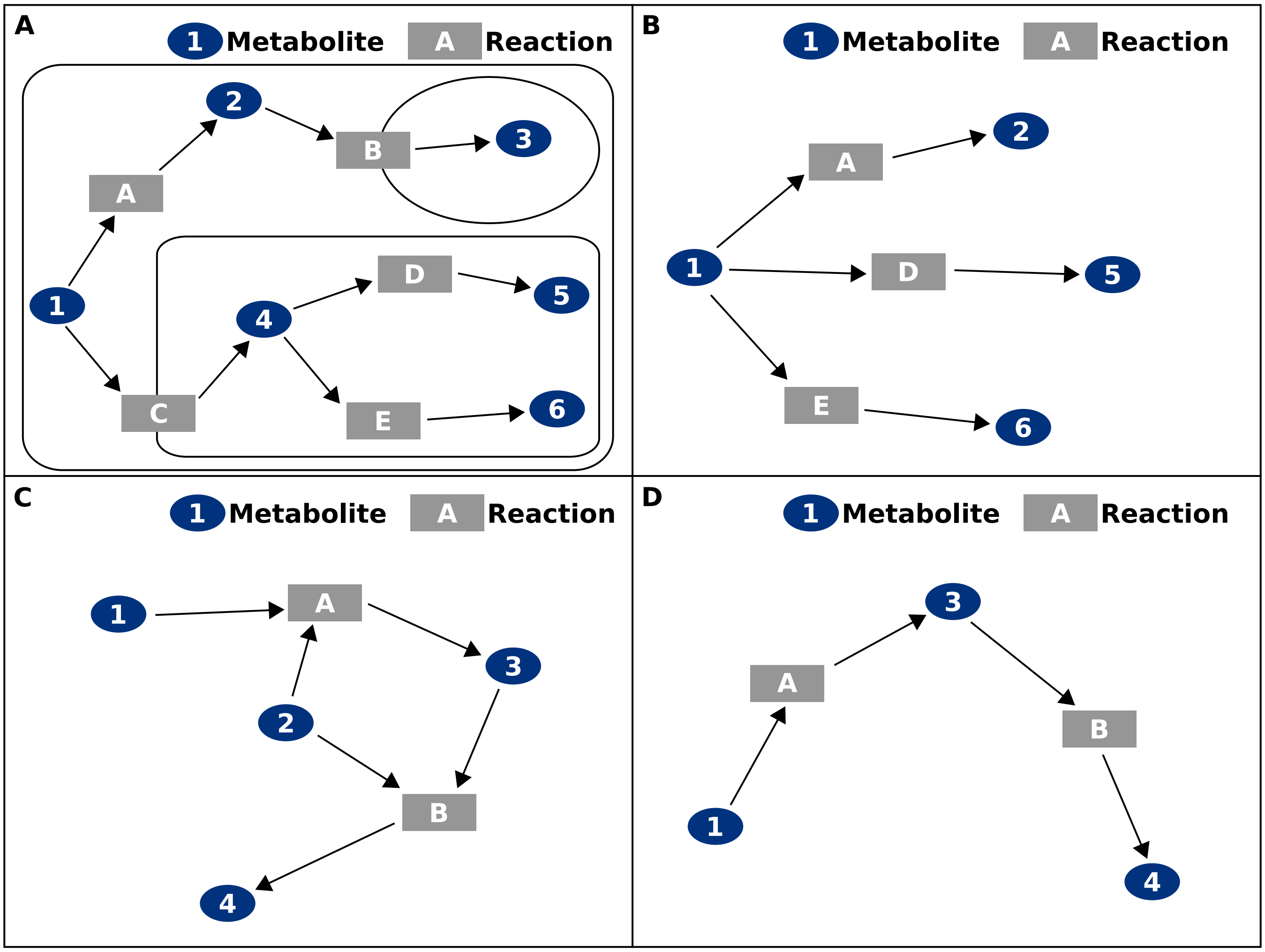

Figure 1. Definition of metabolic networks with simplifications for compartments and hubs. A. Compartmental network. Boxes and ellipse represent compartments. A compartmental network distinguishes between compartmental instances of otherwise chemically identical metabolites (metabolites 1 and 4, metabolites 2 and 3) and reactions. Compartmental networks also include reactions to represent transport (reactions B and C) between compartments. **B.** Non-compartmental version of network from **A**. A non-compartmental network combines representations of otherwise chemically-identical metabolites and reactions to consensus representations (metabolites 1 and 2) and also excludes transport reactions. **C.** Network with hubs. Metabolite hubs (metabolite 2) participate in many reactions and impart excessive connectivity to the network. **D.** Network from **C** without hubs. Selective exclusion of metabolite hubs simplifies the network and reveals major structural themes such as linear or cyclical pathways.

PASy) [20, 21]. Often these metabolic models are specific to cellular metabolism within a single species, and the model of human metabolism has evolved through many iterations and much effort from a broad, collaborative community [33, 34, 35, 36, 37, 12]. Several projects have further defined tissue-specific versions of the human model for greater specificity and accuracy [23, 24]. It is also valuable to integrate and compare models from multiple species, and repositories of common information across multiple species allow standardization, quality control, and comparison. These repositories include BiGG [38], and MetaNetX [16], and relevant tools include MetExplore [39]. Metabolic models are commonly available from repositories in an open format, which is a standard definition of Extensible Markup Language (XML) known as the Systems Biology Markup Language (SBML) [40].

Metabolomic measurements

Metabolomic technologies separate, identify, and quantify small-molecules from biological samples. While some studies utilize Nuclear Magnetic Resonance (NMR), larger studies commonly employ chromatography with gas (GC) or liquid (LC) mobile phases that integrate with various forms of mass spectrometry (MS); and combining measurements from multiple technologies increases the breadth of a study. Each type of technology has its own parameters and requirements for processing and analyzing the data. In particular, normalization to total signal in each sample corrects for loss of material and fluctuation in detector sensitivity. Furthermore, measurements commonly lack absolute calibration such that values only represent relative comparisons between samples. Many data sets from metabolomic studies are not publicly available; however, there are initiatives to include more of these data in public repositories, such as the Metabolomics Workbench [41]. Whereas targeted techniques specifically

Table 1. Curation of human metabolic model

**Step 1**	**Metabolites**	**Reactions**	**Compartments**	**Processes**
Count:	1725	5772	10	113
MetaNetX:	1682 (97.51%)	4474 (77.51%)		
PubChem or HMDB:	951 (55.13%)			
Gene or Enzyme:		3929 (68.07%)		
**Step 2**	**Metabolites**	**Reactions**	**Compartments**	**Processes**
Count:	1725	5772	10	113
MetaNetX:	1682 (97.51%)	4474 (77.51%)		
PubChem or HMDB:	1021 (59.19%)			
Gene or Enzyme:		3929 (68.07%)		
**Step 3**	**Metabolites**	**Reactions**	**Compartments**	**Processes**
Count:	1722	3486	8	109
MetaNetX:	1679 (97.50%)	2771 (79.49%)		
PubChem or HMDB:	1025 (59.52%)			
Gene or Enzyme:		2640 (75.73%)		

Curation of systemic model of human metabolism. The goal of curation was to adapt the model for definition of networks to represent intracellular metabolism, and to improve integration of metabolomic measurements. Step 1 was after integration of Recon 2M.2 [12] with MetaNetX [16]. Step 2 was after deriving names and references for metabolites from HMDB [17]. Step 3 was after curation of individual metabolites and reactions. Summaries comprise counts of metabolites, reactions, compartments, and processes. Summaries also comprise coverage of metabolites with references to MetaNetX [16], HMDB [17], and PubChem [18], and coverage of reactions with references to MetaNetX [16], Entrez Gene [19], and ExPASy [20, 21].

study signals from up to 800 unique and identifiable analytes [11], untargeted studies tend to give much broader coverage and instead search for observable differences in analytes before their identification. Importantly, there are both chemical and technical constraints on the measurable metabolome, and the distribution of detectable metabolites across metabolism is likely nonuniform. This limitation might influence the integration and analysis of metabolomic measurements on metabolic networks.

Analyses

MetaboNet: Tools for definition and analysis of human metabolic networks

MetaboNet [14], is our main collection of parameters and tools to define and analyze metabolic networks. This installable package of code in the Python programming language supports our data transformations and analyses. We host the most current version of this package within a repository on GitHub, and stable version v1.0.0 has a persistent archive [14]. Accompanying the repository on GitHub [14] is a tutorial that explains how to install the package, access necessary external data files, curate the human metabolic model, define customizable metabolic networks, analyze these networks, and integrate metabolomic measurements for functional study. We also published many of MetaboNet's intermediate and final export files in a data archive [15]. Some users may find it more convenient to access these standard export files unless they require further customization of parameters.

Curation and adaptation of the human metabolic model

We curated the latest systemic model of human metabolism and adapted it to provide biological context in metabolic experiments. In particular, our goal was to filter irrelevant reactions from the model while also optimizing our ability to match metabolomic measurements to metabolites. Steps 1 and 2 of curation both comprised enhancements to the information about metabolites and reactions. We accessed the latest model of human metabolism, Recon version 2M.2 [12, 42], and adjusted its format to facilitate importation into MetaNetX [16]. This latter tool was useful to standardize identifiers, control for consensus, and include supplemental reference information about metabolites and reactions. We next matched metabolites to entries in HMDB [17] to standardize common names and to increase coverage of references both to HMDB [17] and to PubChem [18]. Step 3 comprised applying filters and correcting errors. We removed metabolites and reactions that were primarily relevant to simulations of growth and metabolic flux, such as biomass accumulation, protein assembly and degradation, and exchange with the extracellular space or boundary of the system. We next made 197 custom edits for metabolites and 102 custom edits for reactions to improve accuracy and avoid redundancy. Step 3 simplified the model's scale substantially (**Table 1**), effectively reducing noise from our subsequent analyses. Whereas in the original version of the human metabolic model, only 68.07% of 5772 reactions included references to either Entrez Gene [19] or ExPASy [20, 21], 75.73% of our remaining 3486 final reactions include these references (**Table 1**). These external references provide supporting evidence and greater confidence in these final reactions. Similarly, only 55.13% of the original 1725 metabolites included references to either HMDB [17] or PubChem [18], but in our final version of the model 59.52% of 1722 metabolites include these references (**Table 1**). These common metabolite identifiers offer handles by which to match metabolites to metabolomic measurements. A partial explanation for the incomplete coverage of references for metabolites is that both metabolomic experiments and databases likely share a bias for stable, detectable compounds rather than transient metabolic intermediates. Our final adaptation of the human metabolic model is accessible in multiple files and formats within a data archive

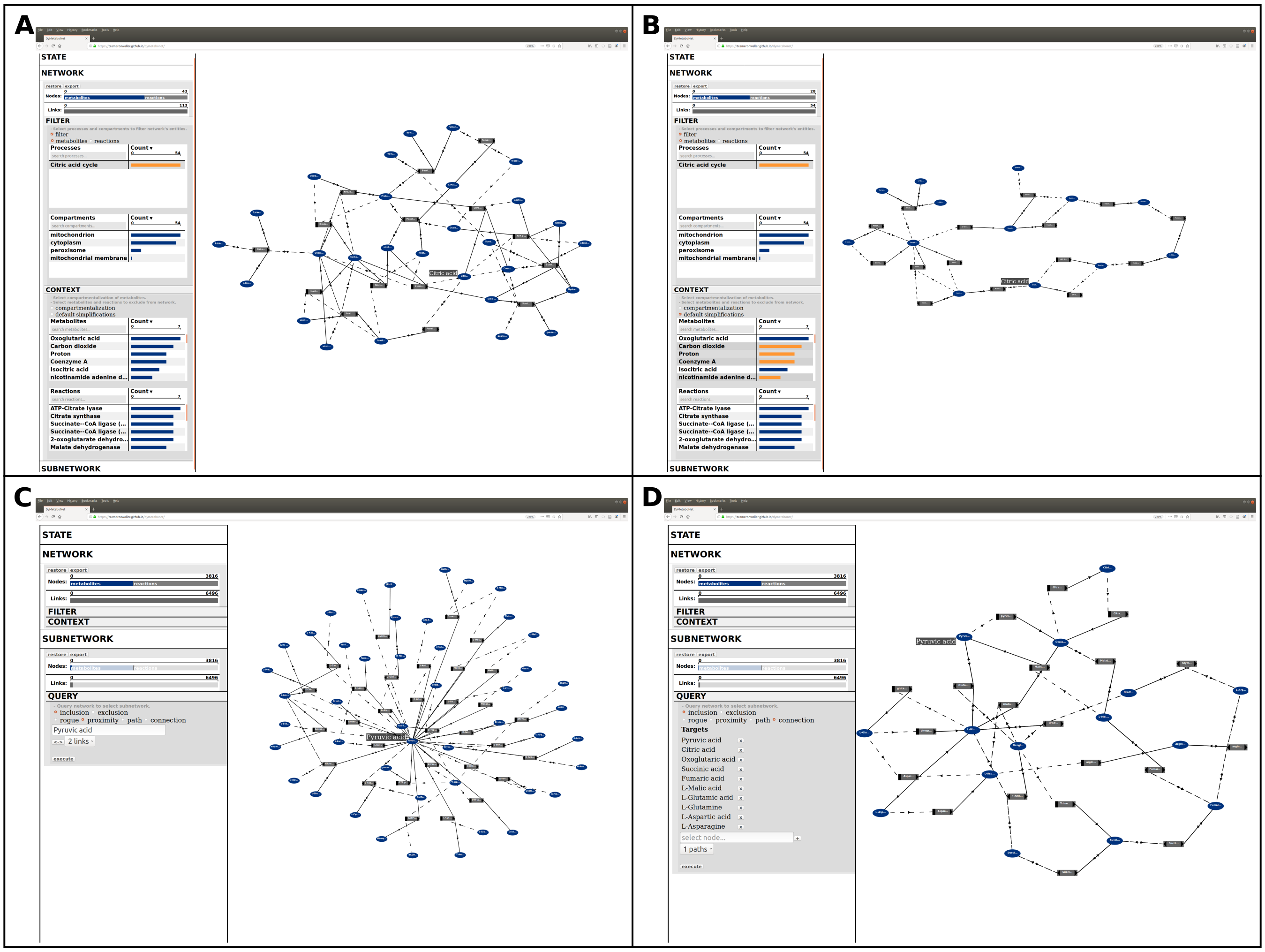

Figure 2. Screen images from DyMetaboNet web application. DyMetaboNet is a web application that defines and visualizes custom metabolic networks within the internet browser. **A.** With hubs, the Citric Acid Cycle has dense connectivity that obscures its cyclical structure. **B.** Exclusion of hubs coenzyme-A, carbon dioxide, proton, and nicotinamide adenine dinucleotide (NAD1+) reveals the overall cyclical structure of the Citric Acid Cycle. **C.** Queries by proximity (breadth-first search) include nodes within specific range of links to a focal node. For example, the user might need to know all reactions in which pyruvate participates. **D.** Connection queries (pairwise simple shortest paths) allow construction of subnetworks between multiple metabolites of interest. For example, the user might need to know how Pyruvate, Citrate, Oxoglutarate, Succinate, Fumarate, Malate, Gutamate, Glutamine, Aspartate, and Asparagine relate to each other. This project's data archive [15] includes a screen-capture video of DyMetaboNet that demonstrates these features and more.

[15]. This model is the basis by which we define and study metabolic networks.

DyMetaboNet: Web application for visual exploration of metabolic networks

To begin our study of human metabolic networks, we designed and developed a tool to visualize the definition and exploration of the human metabolic network. This tool is an experimental prototype that does not intend to replace the broader functionality of major tools in network biology [22]. Rather, our application aims to enhance accessibility and visual interactivity, with integration of basic filters, queries, and visual representations for qualitative exploration. Indeed, this tool emphasized to us some major challenges to the feasibility of applying metabolic networks in metabolomic experiments.

DyMetaboNet [13] is a dynamic, interactive, and qualitative partner to MetaboNet [14]. We host the most current version of this application within a repository on GitHub, and stable version v1.0.0 has a persistent archive [13]. This web application executes code in the JavaScript programming language to control the behavior of visual elements in the web document. DyMetaboNet runs within the user's internet browser without the need to maintain a remote server or install special, local software. The application's graphical interface interactively controls the definition of networks and their visual representation (**Figure 2**), with toggles to represent the network with (**Figure 1A**) or without (**Figure 1B**) compartmentalization, with inclusion (**Figure 1C**) or exclusion (**Figure 1D**) of nodes for specific metabolites, and with filters by cellular compartments and metabolic processes of interest. For example the user might want to consider only reactions and metabolites within the Mitochondrion compartment or those that participate in the Citric Acid Cycle process (**Figure 2B**). As the user alters these controls, DyMetaboNet defines the network accordingly and displays its visual representation nearly in real time, at least for small networks. We acknowledge that there is

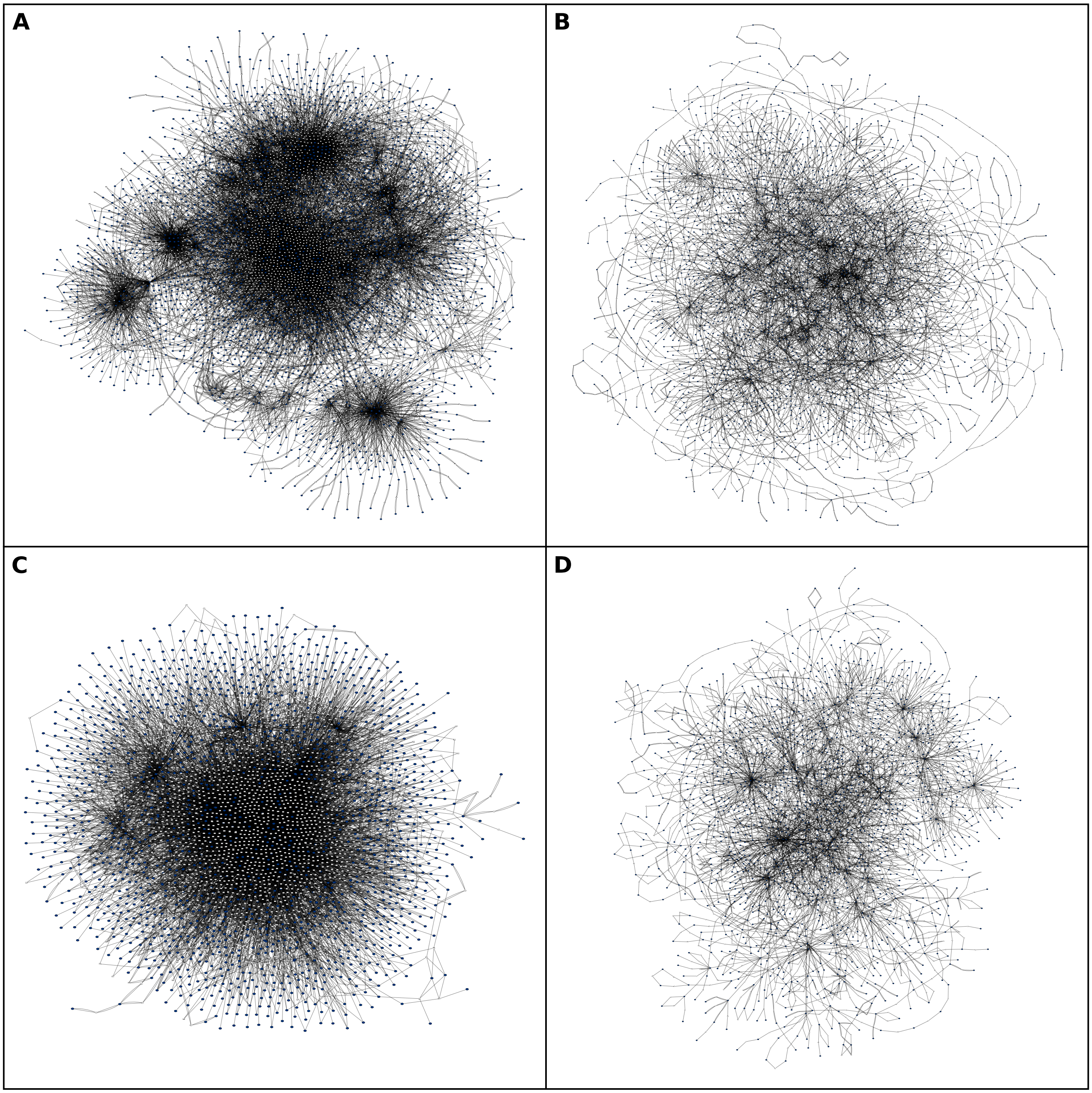

Figure 3. Global structures of metabolic networks. Alternative definitions of metabolic networks differ in global structure. Visual representations of metabolic networks in Cytoscape [22] with identical visual styles and layout parameters. **A.** Compartmental network with hubs. **B.** Compartmental network without hubs. **C.** Non-compartmental network with hubs. **D.** Non-compartmental network without hubs.

substantial latency to compute the layout of larger networks, but visual representation is obscure for networks of this scale anyway. Furthermore, DyMetaboNet supports basic graph-traversal queries to select subnetworks by proximity (breadth-first search) (**Figure 2C**), shortest paths between source and target nodes (directional, simple shortest paths), and pairwise shortest paths between multiple target nodes of interest (**Figure 2D**). A query by proximity might be useful where the user needs to know all reactions in which a single metabolite, such as pyruvate, participates (**Figure 2C**). A query by shortest paths might be useful when the user has measurements for two or more metabolites and needs to know how these relate to each other (**Figure 2D**). The user can export tables of information about metabolites and reactions within these networks and subnetworks. We prepared a screen-capture video demonstration of these features of DyMetaboNet and made this video accessible in a data archive [15]. With its interactive integration of definition, query, and visualization, DyMetaboNet enables a qualitative appreciation for the scale and complexity of the human metabolic network.

During our design and development of DyMetaboNet [13], we

Table 2. Graph-theory properties of metabolic networks.

Network	Order, Total	Order, Metabolites	Order, Reactions	Size	Density	Central, Degree	Central, Between	Path	Cluster	Small World	Assortativity
+ Compartments, + Hubs	6208	2735	3473	18800	9.896E-04	6.912E-05	5.213E-08	6.475E+00	1.052E-01	2.585E+02	9.243E-02
+ Compartments, - Hubs	5609	2428	3181	10003	6.476E-04	6.021E-06	2.073E-08	1.299E+01	1.454E-01	4.342 E+02	2.884E-01
- Compartments, + Hubs	3908	1654	2254	13204	1.771E-03	2.928E-04	1.530E-07	4.743E+00	3.692E-02	6.990E+01	-2.334E-02
- Compartments, - Hubs	3711	1560	2151	6398	9.533E-04	1.837E-05	4.217E-08	1.457E+01	7.653E-02	1.557E+02	1.120E-01

... wrap ...

We define these graph-theory metrics in **Table S2**. Order is respective to bipartite sets of nodes for metabolites and reactions. Values in this table for centralization (degree and betweenness), mean shortest path length, cluster coefficient, small-world coefficient, and degree assortativity are relative only to the bipartite set of nodes for metabolites. Complete metrics for all networks are available in an archive of MetaboNet's complete export data [15].

recognized a need to describe further the variable structure of the metabolic network. It soon became apparent to us that the complexity of the metabolic network involved not only its scale but also its extent of interconnectivity. As DyMetaboNet is a visual interface, both of these aspects made visual representations obscure. Furthermore, a goal of DyMetaboNet was to support graph traversal queries, such as by proximity and shortest paths. Without intervention, we found that such queries were uninformative because common metabolites such as water dominated the network's connectivity and hence its shortest paths. Finally, DyMetaboNet's qualitative perspective emphasized extreme differences between alternative but reasonable definitions of the metabolic network. These observations impressed us as major challenges to the feasibility of contextualizing metabolomic experiments on appropriate metabolic networks. We therefore decided to pursue deeper analysis of the metabolic network's structure and its dependence on reasonable differences in definition.

Definition of metabolic networks

We defined multiple network representations of human metabolism. Networks are abstract simplifications of complex systems, and alternative representations can be reasonable while emphasizing different aspects of the underlying information. We chose to keep some definitions consistent, while altering other constraints to evaluate their influence. All of our definitions represent metabolism in a directional bipartite network [43] with distinct types of nodes for reactions and metabolites (**Figure 1D**). This representation is intuitive for interactions between distinct biological entities. Metabolites are small molecules, whereas reactions are chemical events that comprise roles of genes, transcripts, and proteins. Accordingly, nodes in this network store attributes that match their type of biological entity. Directional links between these nodes depict relations between metabolites and reactions, including which metabolites participate as reactants and products and whether the reaction is reversible (**Figure 1D**). Whereas all links in our networks are weightless, we think it worthwhile to comment briefly on the alternative. Assigning weights to reactions' links might reasonably represent the metabolically significant conversion of chemical mass or the rates of metabolic flux; however, these metrics can be variable (specific to tissue and experimental condition) and difficult to measure. Keeping these aspects (bipartite nodes for metabolites and reactions, directional weightless links) of our definitions consistent allowed us to compare differences when varying other constraints. These additional constraints include compartmentalization (**Figure 1A, Figure 1B**), filters by compartment and process, and exclusion of nodes for specific metabolites (**Figure 1C, Figure 1D**). During our work on DyMetaboNet [13], we found these factors to have a strong effect on the metabolic network's structure.

Constraint 1: Compartmentalization

Our first constraint involves compartmentalization. Compartmental networks (**Figure 1A**) include compartment-specific instances of otherwise chemically identical metabolites and reactions. These networks also include reactions to represent transport between compartments. Non-compartmental networks (**Figure 1B**) aggregate these chemically identical metabolites and reactions into single, consensus representations that are each unique. These networks also exclude transport reactions as these are irrelevant without compartments.

Constraint 2: Filters by compartments and processes

Our second constraint involves filters by specific cellular compartments and metabolic processes. These compartments and processes define sets of metabolites and reactions of interest. For example the user might want to consider only reactions and metabolites within the Mitochondrion compartment. Similarly, the user might want to consider only reactions and metabolites that participate in the Citric Acid Cycle process (**Figure 2B**). MetaboNet [14] makes these filters customizable. In subsequent analyses, we included metabolites and reactions from all compartments and processes to establish a perspective on the entirety of cellular metabolism.

Constraint 3: Exclusion of specific metabolites

Our third constraint relates to the exclusion of specific metabolites from the metabolic network. This exclusion means that the network does not include nodes to represent these metabolites, and consequently there are also no links to or from them. Regardless of exclusion of nodes and links for a metabolite, reactions themselves still include information about all metabolites that participate as reactants and products.

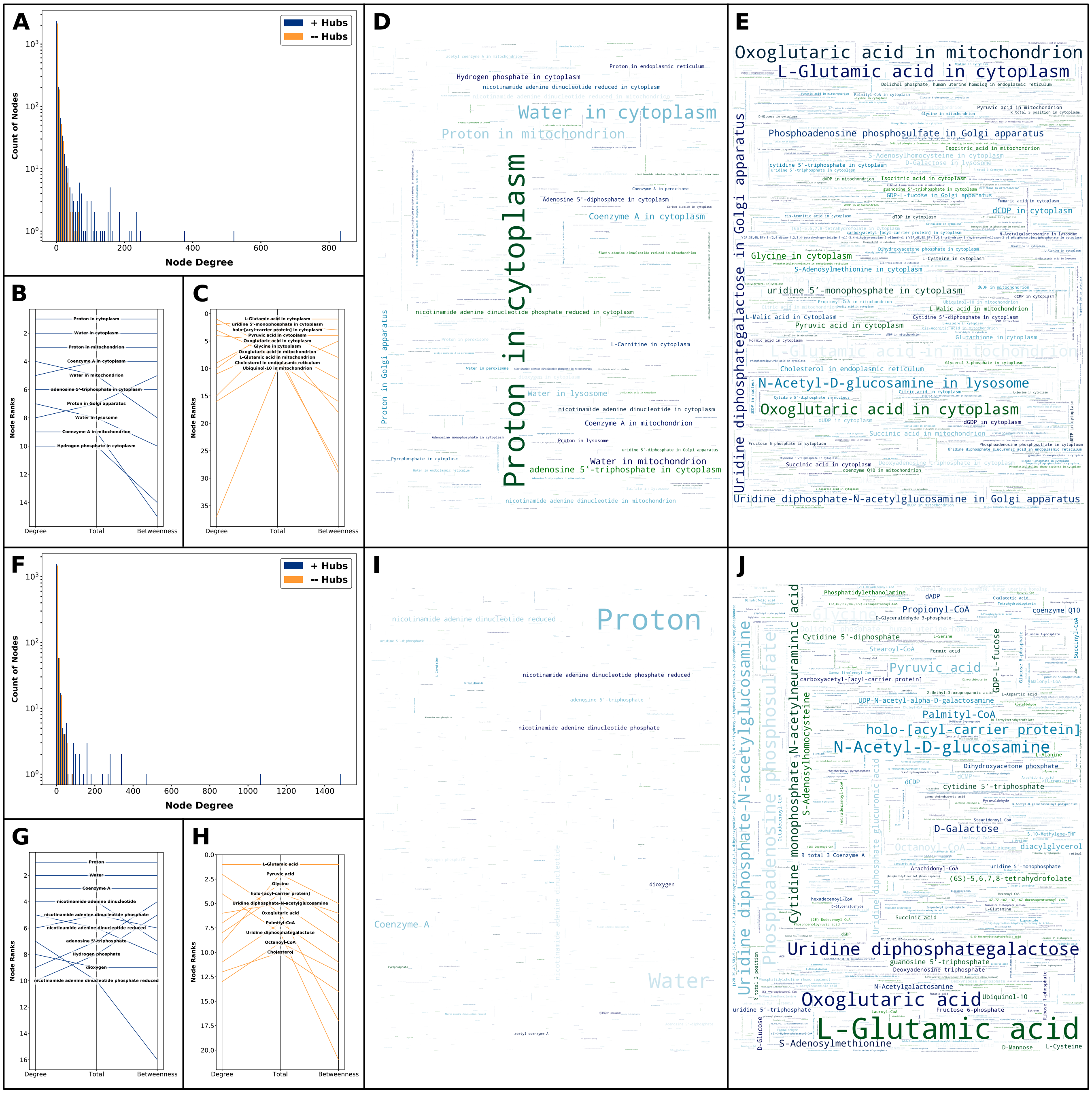

Figure 4. Properties of metabolites' nodes in metabolic networks. Alternative definitions of metabolic networks differ in dominant influence of metabolites' nodes. **A–E.** Upper panels represent compartmental metabolic networks. **F–J.** Lower panels represent non-compartmental metabolic networks. **A, F.** Histograms for counts of metabolite nodes with specific degrees in networks with and without hubs. **B, C, G, H.** Parallel coordinates charts for ranks of metabolites' nodes in metabolic networks by degree centrality ("Degree"), betweenness centrality ("Betweenness"), or a mean of these ranks ("Total"). **B, G.** Ranks of metabolites' nodes in metabolic networks with hubs. **C, H.** Ranks of metabolites' nodes in metabolic networks without hubs. **D, E, I, J.** Word cloud visual representations of the influences of metabolites' nodes in metabolic networks with nodes' degrees scaled to font size by a factor of 1.0. **D, I.** Influences of metabolites' nodes in metabolic networks with hubs. **E, J.** Influences of metabolites' nodes in metabolic networks without hubs.

Metabolite hubs are special candidates for exclusion from the metabolic network. A few metabolites are common reactants and products in metabolic reactions, such that they contribute a large proportion of the connectivity in metabolic networks (**Table S1**) [11]. These metabolites are hubs, and they are of special interest because they dominate the network's structure. Exclusion of these hubs simplifies connectivity (**Figure 1C–D, Figure 2A–B**) and improves resolution to detect trends in other, less dominant metabolites. We divided these hubs into 2 conceptual categories on the basis of their relevance to metabolic regulation and experiments.

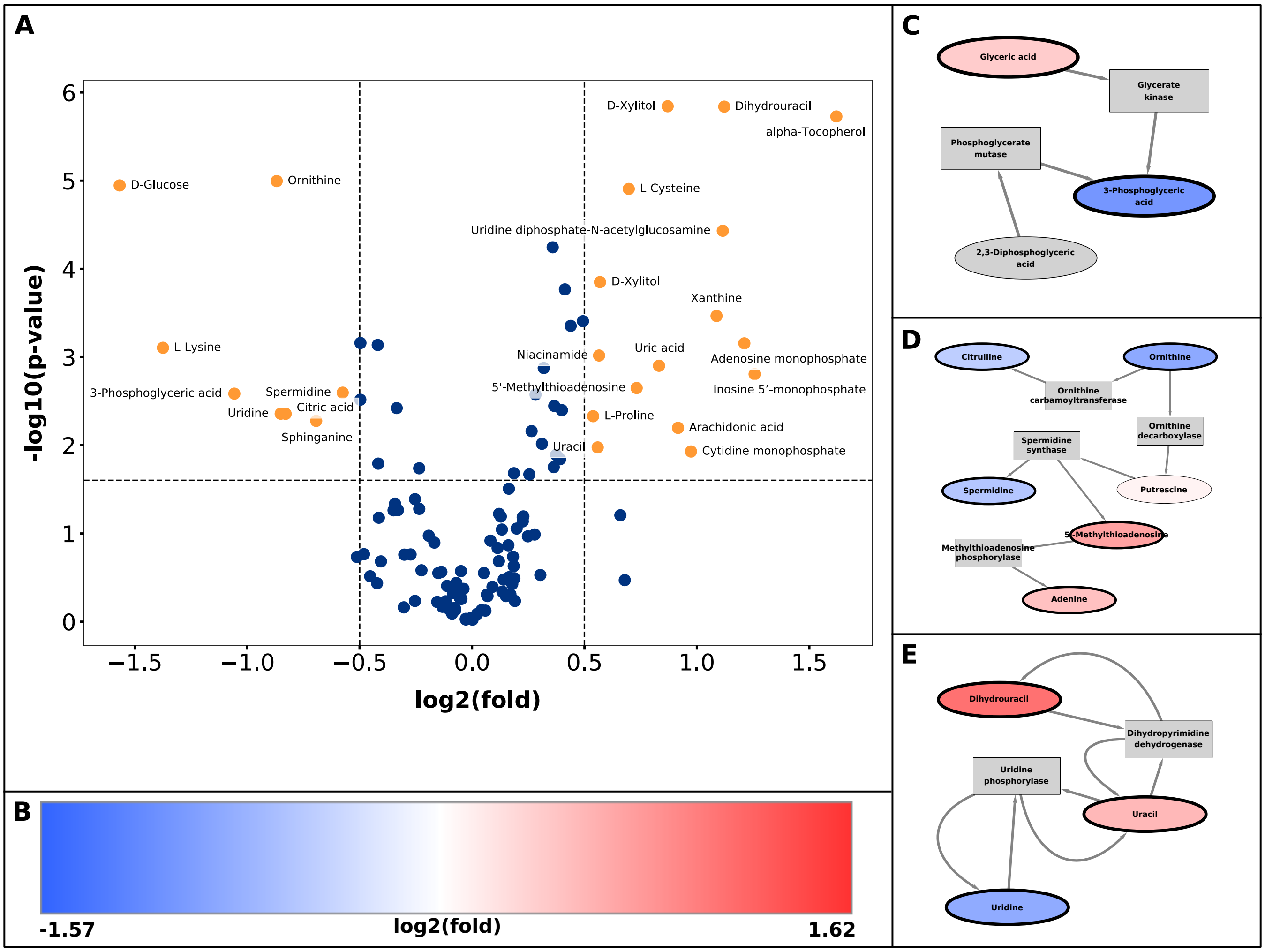

Figure 5. Integration and analysis of metabolomic measurements on metabolic networks, Study 1. Metabolomic Study 1 (**Table S3**) compared the abundances of 177 metabolites between cancerous and normal lung tissues. Clusters of enrichment in fold changes are detectable by integrating measurements within the non-compartmental network without hubs (**Figure 3D**). **A.** Volcano plot of p-values and fold changes in metabolites. **B.** Scale for color representation of fold changes on nodes in clusters. Extremes of color scale represent the minimal and maximal fold changes in the entire study. **C–E.** Clusters in metabolic network are detectable by enrichment of p-values and fold changes. Metabolite nodes in clusters represent fold changes by color fill, and they represent p-value by border thickness ($p\text{-value} < 0.05$).

Category 1 Hubs. Category 1 metabolite hubs are less relevant to metabolic regulation and experiments. Many of these metabolites are prolifically abundant in the cell. While they are all chemically essential to metabolic reactions, some of these metabolites, such as water, dioxygen, and carbon dioxide, are unlikely to participate in the type of metabolic regulation that is commonly relevant to experiments. Furthermore, perturbations in the abundance of these metabolites would be difficult to interpret, and some of these metabolites are undetectable in metabolomic measurements.

> Category 1 Hubs (**Table S1**): proton, water, dioxygen, phosphate, diphosphate, carbon dioxide, sulfate, hydrogen peroxide, ammonium, sulfite, sodium, hydrogen carbonate, hydroxide

Category 2 Hubs. Category 2 metabolite hubs are more relevant to metabolic regulation and experiments. The abundance of these metabolites in the cell fluctuates in metabolic regulation, and they are relevant to many metabolic experiments. However, some of these metabolites participate in so many reactions that they dominate connectivity in the metabolic network. Exclusion of these metabolites from the metabolic network reveals more subtle trends involving the influences of other metabolites that are of greater interest in some contexts.

> Category 2 Hubs (**Table S1**): coenzyme-A, acetyl coenzyme-A, acyl-carrier protein, carnitine, nicotinamide adenine dinucleotides, flavin adenine dinucleotides, nucleoside phosphates

This constraint for exclusion of specific metabolites is very sensitive and requires customization to the context of each metabolic experiment. Consequently, MetaboNet [14] makes the selection of these metabolites customizable. For our subsequent analyses herein, we chose to evaluate the extreme condition with exclusion of all metabolite hubs in Category 1 and all metabolite hubs in Category 2 with degrees greater than 50 (**Table S1, Table S2**). Degree is a metric of a node's connectivity in a network that we discuss in greater detail later on. We found that the exclusion of these hubs simplified metabolic networks profoundly and exposed intrinsic structure that enhanced the potential to detect

relevant clusters in our retrospective analyses of metabolomic measurements. This extreme approach may not be appropriate for all experiments, and metabolite hubs in Category 2 deserve particular attention in the selection of metabolites for exclusion.

Analysis of metabolic networks

We next set out to describe and compare our metabolic networks both qualitatively and quantitatively. MetaboNet [14] exports networks to file formats compatible for import to Cytoscape, and we used this latter tool to visualize global networks at high resolution (**Figure 3**). Within MetaboNet, we also applied multiple metrics from graph theory (**Table S2**) to describe and compare our metabolic networks (**Table 2**). These bipartite networks have distinct metrics of centrality, centralization, path-length, cluster coefficient, small-world coefficient, and degree assortativity relative to their nodes for metabolites and reactions [43]. We chose to concentrate our analyses on these metrics relative to metabolites (**Table 2**), as our primary interest is in the flow of mass within the metabolic network and its measurement in metabolomic experiments. Complete metrics for all networks are available in an archive of MetaboNet's complete export data [15].

Like many other real systems, metabolism is a small world [9]. The small-world pattern appears pervasively across networks representing real systems, including friendships between people and connections between internet servers. In a small world of friendships, any person knows another person vicariously through a few other people, whereas in a small world of internet servers, any computer communicates with another computer by transferring information through a few intermediate servers. In addition to their short path lengths, small-world networks share strong modularity. This structure favors specialization and versatility while also allowing for cooperative communication. These characteristics imply some combination of stochasticity and selection in the formation of these networks, and the same principles apply to the evolution of biological systems [9]. All of our metabolic networks have extreme values ($\gg 1.0$) of the sigma small-word coefficient (**Table S2**, **Table 2**), and both compartmental and non-compartmental networks with hubs have mean path lengths that are less than the natural logarithms of their orders (**Table S2**, **Table 2**). This strong, small-world character suggests that the metabolic system relies heavily on modularity but also that there is efficient communication and cooperation between these modules [9]. Importantly, this structure implies that both regulatory signals and perturbations pervade the entire system readily.

Compartmentalization confers major structural properties to metabolic networks. In their global visualizations, compartmental (**Figure 3A,B**) and non-compartmental (**Figure 3C,D**) metabolic networks are noticeably distinct; however, this difference is most apparent between the networks with hubs (**Figure 3A,C**). In this case, compartmentalization introduces dramatic clusters or modules throughout the network (**Figure 3A**), giving the impression that compartments divide and disperse the metabolic system, decreasing its connectivity. This effect is less apparent between the networks without hubs (**Figure 3B,D**). To explore these differences further, we applied multiple metrics from graph theory (**Table S2**, **Table 2**). The most obvious observation from this analysis is that both compartmental networks have greater orders and sizes than their non-compartmental counterparts (**Table 2**). This difference in non-compartmental networks reflects the absence of replicate nodes for compartmental instances of chemically-identical metabolites and reactions (**Figure 1**) along with the exclusion of reactions that mediate transport across membranes (**Figure 1**). Another observation is that compartmentalization creates networks with lesser density and centralization (**Table 2**). Consistent with their global visualizations (**Figure 3**), these shifts in density, close-range (degree) centralization, and long-range (betweenness) centralization, are greater in the networks with hubs (1.79, 4.24, and 2.93 fold respectively) than in those without hubs (1.47, 3.05, and 2.03 fold respectively) (**Table 2**). Compartmentalization also imparts greater cluster coefficients, with the shift greater with hubs (2.85 fold) than without them (1.90 fold) (**Table 2**). Whereas mean path lengths and degree assortativity vary little, compartmentalization imparts greater small-world coefficients both with hubs (3.70 fold) and without (2.79 fold) (**Table 2**). Together these observations have interesting biological implications. Compartmentalization decreases connectivity in metabolism (density, centralization) to avoid excessive communication and interaction, such as through enzyme promiscuity and spurious allosteric interactions between metabolites and proteins [44]. Conversely, compartmentalization also increases modularity (cluster coefficient), allowing for specialization and regulation within separate chemical environments. Surprisingly, this increase in modularity combines with subtle changes to path lengths such that compartmentalization actually enhances the small-world character of metabolic networks. Furthermore, it is interesting that hubs effectively exaggerate most of these effects, and we consider them next.

Metabolite hubs dominate connectivity within metabolic networks. In their global visualizations, networks with (**Figure 3A,C**) and without (**Figure 3B,D**) hubs are strikingly distinct. These hubs introduce apparent connectivity to both compartmental (**Figure 3A,B**) and non-compartmental (**Figure 3C,D**) networks. As before, metrics from graph theory (**Table S2**, **Table 2**) elucidate these differences. An obvious observation is that networks without hubs have lesser orders and sizes than their comparisons (**Table 2**), due to the exclusion of nodes for these metabolites (**Table S1**). Also intuitive from their definition is the observation that hubs impart greater density and centralization (**Table 2**). These shifts in density, close-range (degree) centralization, and long-range (betweenness) centralization, are greater in non-compartmental networks (1.86, 15.9, and 3.63 fold respectively) than in compartmental networks (1.53, 11.5, and 2.51 fold respectively) (**Table 2**). Notably, hubs decrease mean path lengths (**Table 2**) for both non-compartmental (3.07 fold) and compartmental networks (2.01 fold), suggesting that these hubs dominate the majority of shortest paths in their networks. Hubs also decrease cluster coefficients and small world coefficients (**Table 2**) both without (2.07 fold and 2.23 fold respectively) and with (1.38 fold and 1.68 fold respectively) compartments. Hubs also decrease assortativity with and without compartmentalization (**Table 2**). Together these observations are relevant to the study of metabolic networks. Metabolite hubs are likely to dominate shortest paths in network traversal queries, and they also are likely to obscure detection of clusters of interest. Their influence is even more profound in non-compartmental networks. Accordingly, the selection of hub metabolites for exclusion (**Table S1**) from the network is an important parameter.

Ranks of metabolites in metabolic networks

To compare metabolic networks from a complementary perspective, we considered their profiles of prominent metabolite nodes. We noticed that metabolites' nodes in these networks have degrees that follow roughly exponential distributions (**Figure 4A,F**), an indication of a scale-free network [9]. Importantly, the exclusion of metabolite hubs has its greatest effect on those few nodes with the greatest degrees in both compartmental (**Figure 4A**) and non-compartmental (**Figure 4F**) networks. We next sought to rank metabolites by relative influence or weight, combining metrics for degree centrality and betweenness centrality. Hence, our ranks (**Figure 4B-C,G-H**) represent the close range (degree centrality) and long range (betweenness centrality) influence of metabolites in metabolic networks [45]. In both compartmental and non-compartmental networks, the exclusion of hubs changes dramatically the metabolites with prominent influences (**Figure 4B-E,G-J**). Specifically, the exclusion of hubs such as proton, water, coenzyme A, nicotinamide adenine dinucleotides, adenosine triphosphate, hydrogen phosphate, and dioxygen (**Figure 4B,D,G,I**) allows for other metabolites to rise to prominence, such as glutamate, pyruvate, glycine, oxoglutarate, and cholesterol (**Figure 4C,E,H,J**). These latter metabolites are more common targets of interest in metabolic regulation and metabolomic experiments.

Glutamate is an impressive example of connection and cooperation in metabolism. While not equal to hub status, this metabolite is particularly promiscuous in metabolic reactions. In both the compartmental and non-compartmental networks without hubs, glutamate is the top-ranking metabolite in terms of both its close and long-range influence (**Figure 4C,H**). In the non-compartmental metabolic network without hubs, this amino acid participates in more than 60 reactions in at least 3 different cellular compartments, belonging to about 30 different metabolic processes. Furthermore, glutamate belongs to 25 different sets within MetaboAnalyst's default library for metabolite set enrichment analysis [46]. Whereas analyses of sets isolate Glutamate's various roles, analyses of networks integrate these for a holistic perspective. Glutamate illustrates the importance of studying metabolism as an entire system, not as arbitrarily separate sets of distinct pathways. Perturbations of this central metabolite are likely to have pervasive effects on the metabolic system, but they might also be difficult to interpret in any specific context.

Glutamate's metabolic sets [46]

Malate-Aspartate Shuttle; Glucose-Alanine Cycle; Alanine Metabolism; Glutathione Metabolism; Cysteine Metabolism; Phenylalanine and Tyrosine Metabolism; Folate Metabolism; Urea Cycle; Lysine Degradation; Ammonia Recycling; Amino Sugar Metabolism; Beta-Alanine Metabolism; Aspartate Metabolism; Nicotinate and Nicotinamide Metabolism; Propanoate Metabolism; Histidine Metabolism; Glutamate Metabolism; Arginine and Proline Metabolism; Warburg Effect; Glycine and Serine Metabolism; Tryptophan Metabolism; Valine, Leucine and Isoleucine Degradation; Arachidonic Acid Metabolism; Tyrosine Metabolism; Purine Metabolism

Selection of metabolic networks for application to metabolomic experiments

Reasonable definitions of metabolic networks differ to the extent that their application to metabolic experiments warrants careful selection. These networks offer potential to facilitate design of experiments and to contextualize functional interpretations of metabolomic measurements. Importantly, our analyses demonstrate that constraints by compartmentalization and metabolite hubs alter the structure of the metabolic network substantially (**Figure 3**, **Table 2**, **Figure 4**). Hence, it is reasonable to assume that any subsequent integration and analysis of measurements will depend on the definition of the network itself. The first constraint to consider is compartmentalization. Standard metabolomic techniques do not distinguish between cellular compartments; rather, a measurement for an analyte, such as glutamate, represents the total abundance of that analyte in all types of cells and in all sub-cellular organelles within a sample. Mapping non-compartmental metabolomic measurements onto a compartmental metabolic network would require replication across compartmental instances of each metabolite, and it would be difficult or impossible for this replication to represent the respective sizes of compartmental pools of the metabolite accurately. Selection of a non-compartmental network would avoid the risk of introducing artifacts or bias from this replication of measurements. On the other hand, selection of a compartmental network could enhance functional interpretation by introducing relevant biological context. The second constraint to consider is the inclusion of metabolite hubs. These hubs would tend to dominate topological queries on the network, and they would also obscure the detection of clusters of relevant measurements within the network. Hence the careful selection of hubs for exclusion is very important. Rather than attempting an exhaustive comparison, we selected the non-compartmental network with exclusion of default metabolite hubs (**Table S1**). We then performed a trial of integration and analysis of metabolomic measurements on this network.

Preparation of metabolomic measurements

Having selected the non-compartmental metabolic network without hubs, we next prepared to evaluate its application to retrospective analyses on real metabolomic measurements. As our model and networks represent intracellular human metabolism, we searched for studies on solid human tissues, rather than plasma, serum, other body fluids, or excrement. We selected 5 studies with publicly-accessible metabolomic measurements [41] on samples from fat, lung, liver, and muscle tissues from human participants (**Table S3**) [47, 48, 49, 50, 51, 52, 53]. Studies 1, 2, and 5 are of particular interest as they include pairs of samples from the same persons across experimental groups, and studies 1, 2, 3, and 4 have previous publications that analyze and interpret trends in metabolites [47, 49, 51].

We organized metabolomic measurements to compare experimental groups in each study. Each of the five studies includes measurements for 125 or more identifiable analytes, of which at least 70 (> 57%) match to metabolites in our model of human metabolism (**Table S3**). We normalized these measurements to the total signals for each sample to control for confounding variance from sample loss or instrument sensitivity. We then compared each metabolite's abundance between experimental groups (**Table S3**), calculating probabilities (p-values) by the student's t-test and also calculating the base-two logarithms of fold changes. For each study, we visualized these fold changes and probabilities simultaneously in volcano plots (**Figures 5A, S1A, S2A, S3A, S4A**). These plots effectively emphasized metabolites with both great differential abundance and great precision in their measurements, and demonstrated trends of accumulation and depletion in metabolites (**Figures 5A, S1A, S2A, S3A, S4A**) that were consistent with those in previous publications of these studies

[47, 49, 51].

Analysis of metabolomic measurements by set and network enrichment strategies

As a trial application, we compared analyses of metabolomic measurements by a standard set enrichment method and by a general cluster enrichment method on our metabolic network. For the analysis by metabolite set enrichment, we used MetaboAnalyst [46], a versatile and popular tool with an accessible web interface. For the analyses by enrichment in the network's clusters, we integrated fold changes and probabilities (p-values) from metabolomic measurements to matching metabolites in the non-compartmental metabolic network without hubs. We then exported this network with measurements to Cytoscape [22], within which we used the jActiveModules application [54, 55] to detect clusters with enrichment in measurement probabilites (p-values). Finally, we searched for clusters exhibiting patterns of both accumulation and depletion in proximal metabolites (Figure 5, supplemental figures, **Table S4**, **Table S5**). Further details on these analyses including methods, observations, and preliminary interpretations are in this article's sections for Methods and Supplement.

Our analyses reiterated some of the advantages of modeling biological systems as networks rather than disjoint sets [56, 11]. We found that the set enrichment analysis was prone to over interpretation of measurements from a few prominent analytes, with vulnerability to artifacts and false-positives (**Table S6**). In contrast, searching for network clusters exposed trends in a greater diversity of analytes (**Figures 5, S1, S2, S3, S4; Table S4**), most of which did not occur in the top ten hits from set enrichment analysis (**Table S6, Table S4, Table S5**). Furthermore, several clusters were novel even after comparison to the original publications on these studies [47, 49, 51]. Interestingly, several of these clusters occupied intersections between major metabolic processes and between separate intracellular compartments (**Figures 5, S1, S2, S3, S4; Table S5**). In conclusion, the network enrichment analysis demonstrated sensitivity to even subtle trends, with resolution to identify individual genes, transcripts, and proteins that were candidates for differential regulation between experimental conditions (**Table S5**). Our definition of the metabolic network suited these analyses and demonstrated potential for further integration in methods for high-resolution and high-throughput analysis of -omic measurements from metabolic experiments.

Discussion

In this project, we describe the effects of compartmentalization and metabolite hubs on distinct definitions of systemic metabolic networks. To do so, we derived information about metabolic reactions from our own custom curation of the latest model of human metabolism [12] and its integration with other databases [16, 17, 18]. We developed both a visually interactive web application, DyMetaboNet [13], and a customizable package of parameters and code, MetaboNet [14], to define different network representations of human cellular metabolism. By applying metrics from graph theory–such as centralization, mean shortest path, cluster coefficient, small-world coefficient, and assortativity–to these networks, we described major structural distinctions that depend on compartmentalization and metabolite hubs. These factors differentiate the biological context accessible for integration and analysis of -omic measurements within these networks. As a trial application, we selected the non-compartmental network without metabolite hubs for a retrospective analysis of metabolomic measurements [41] from multiple studies on human tissues (**Table S3**) [47, 49, 51]. We found that a general network enrichment strategy [22, 54, 55] has potential to detect biologically relevant differences at junctions between metabolic pathways.

Compartmentalization of metabolic reactions and intermediates within intracellular organelles and membranes establishes regulatory environments with chemical specialization. Extensive interconnectivity between reactions within these separate environments contributes to the overall modularity of the network, a structure that enhances the evolution, versatility, and robustness of the entire system [9, 57]. Indeed, we observed that compartmentalization increases the cluster coefficients and small-world coefficients of metabolic networks. Compartments are also important to avoid excessive interactions within metabolism, such as through feed-forward and feed-back allosteric activation or inhibition [44], as well as enzyme promiscuity; however, these partitions do not entirely isolate their environments. There is extensive, efficient communication and cooperation across cellular borders, with specific signaling mechanisms, transport events, and even physical connections between organelles to regulate and enhance these processes [58]. Consistent with this communication between compartments was our somewhat surprising observation that compartmentalization does not appreciably alter the mean shortest path length between metabolite nodes within the network. Compartmentalization certainly contributes major regulation to Eukaryotic metabolism, and its representation in metabolic networks warrants careful consideration.

Hubs are a common and influential pattern in network representations of real systems, with particular relevance to metabolism. Early studies on the topological structures of biological networks (gene interactions, protein interactions, and metabolic reactions) [9, 57] described their dissortativity, with selection against direct connections between hubs. This structure contrasts with the assortativity that is common in other real systems, such as social networks in which very friendly people are more likely to know other very friendly people. While dissortativity enhances modularity in biological networks, at the extreme it leaves these systems vulnerable to loss of essential modules. Further analysis of biological networks revealed a dichotomous combination of dissortative major hubs with assortative minor hubs [59], balancing the benefits of modularity while mitigating the vulnerabilities of disconnection. Here, we studied structural dependencies by omitting metabolite hubs from metabolic networks; although, we acknowledge that an alternative and more moderate simplification of these hubs would be to assign weights to all links in the network, with lesser weights for links to and from hubs. We designated metabolite hubs on the bases of their chemical and metabolic properties and their connections within the network; very small molecules and ions with prolific abundances in and around the cell (proton, water, dioxygen, carbon dioxide, phosphate, etc.) were hubs along with metabolites with degrees beyond a specific threshold (coenzyme A, nicotinamide and flavin adenine dinucleotides, adenosine phosphates, etc). We found that excluding nodes and links for these metabolite hubs exposed impressive structural dependencies in metabolic networks. Not only do hubs dominate shortest path lengths between metabolite nodes, they also decrease the apparent modularity in terms of cluster coefficients and small-world coefficients. Furthermore, we found that these hubs also decrease the assortativity (increase the disassortativity) of their networks, emphasizing the relevance of the dissortative and assortative dichotomy

in metabolism as a quantitative explanation and justification for this strategy of simplification [59, 11]. Careful representation of hubs in metabolic networks can expose subtle structure and also improve resolution in network traversal queries.

Together, MetaboNet and DyMetaboNet demonstrate useful methods and designs for analysis of metabolomic data. DyMetaboNet emphasizes qualitative exploration of a coherent metabolic system, by integrating the definition and query of networks with their visual representations in an interactive interface. In particular, network queries by proximity (breadth-first search) and paths between two or more targets (pairwise shortest simple paths) enhance this exploration. DyMetaboNet's perspective contrasts with other tools that represent metabolism as a collection of discrete pathways, each with its own static, manually-drawn map. Examples include KEGG Atlas [26], Reactome [28], and Escher [60]. A limitation is DyMetaboNet's requirement for automatic layouts in order to draw diagrams of custom networks. These automatic layouts are often less readable than manual layouts and are computationally expensive for large networks. Also, DyMetaboNet's compact web application excels at accessibility, interactivity, and integration of controls and visualizations; but these advantages disappear due to latency for larger networks or tasks that require more functionality. In these scenarios, broader-feature applications such as Cytoscape and Metscape [22, 61] are preferable. Indeed, after defining global metabolic networks in MetaboNet, we transferred these to Cytoscape for further visualization and analysis. Within Cytoscape, we used the jActiveModules [54, 55] application to detect generic enrichment in probabilities (p-values) on clusters of proximal nodes. Our use of this general cluster enrichment method was exploratory, and we acknowledge the potential for novel network enrichment algorithms to account for reaction directionality and patterns of accumulation and depletion in proximal metabolites. Integrating such a metabolism-specific clustering algorithm, together with detection of patterns across functional categories of reactions (processes, compartments) [46] and chemical classes of metabolites [11] might help to prioritize and quantify targets in metabolomic measurements [10].

Potential implications

Biological models offer the potential to integrate holistic, functional context in interpretations of -omic measurements [7, 8]. As biological systems thrive on cooperative interactions between diverse types of entities, computational models tend to simplify these systems within distinct dimensions, such as networks of gene-gene, protein-protein, and protein-metabolite interactions [1]. At the systemic scale, comparatively little is known about this last dimension of protein-metabolite interactions [62]; although, decades of reductionist experiments demonstrate the functional relevance of catalysis, transport, and allosteric regulation. Whereas our work here emphasizes only the representation of catalysis and transport in metabolic reactions, we await further exploration of allosteric interactions between proteins and metabolites, whether within an enzymatic active site or otherwise. This exploration requires technological innovation to accommodate low-affinity interactions and the chemical diversity of the metabolome. Pioneering work employs either informatic datamining [44, 63] or measurements by mass spectrometry to detect physical protein-metabolite interactions [64, 65, 66]. We anticipate that forthcoming, systemic models of allosteric protein-metabolite interactions will be valuable to integrate with those in metabolic models. These developments will advance the goal of integrating multidimensional representations of molecular biology [1].

Methods

Procedures for curation, definition, and analysis of human metabolic networks

We developed the MetaboNet package [14] as a transparent and reproducible record of our curation of the Recon 2M.2 metabolic model [12] and our definition, and analysis of human metabolic networks. This package includes editable tables of parameters to customize curation and definition of these networks. Collections of scripts in the Python programming language automate these procedures. MetaboNet employs functionality from the SciPy [67], NumPy [68], NetworkX [69], MatPlotLib [70, 71], and WordCloud [72] packages.

MetaboNet requires sources of information from the Recon 2M.2 model of human metabolism [12], version 4.0 of HMDB [17], and metabolomic measurements from studies in the Metabolomics Workbench [41]. MetaboNet produces exports for integration and further analysis in MetaNetX [16], DyMetaboNet [13], NetworkX [69], MetaboAnalyst [46], and Cytoscape [22]. MetaboNet's README [14] gives more information about installation, customization, and execution of these procedures. MetaboNet is available on GitHub under version 3 of the GNU General Public License [14].

Curation and adaptation of human metabolic model

We accessed the latest model of human metabolism (**Table 1**). We accessed information for the Recon 2M.2 model of human metabolism [12] from file "Recon2M.2_MNX_Entrez_Gene.xml" (14.2 Megabytes) in record "583326" of the Zenodo repository [42]. The format of this file is consistent with level 2 and version 4 of the Systems Biology Markup Language (SBML) [40], a specification of the Extensible Markup Language (XML). This version of the Recon 2M.2 model uses derivatives of identifiers and names for metabolites from the MetaNetX [16] name space and references records in Entrez Gene [19] for specific genes relevant to reactions.

We used the tools and repository of MetaNetX [16] and version 3.2 of the MNXref namespace to check for consistency and quality and to standardize the identifiers and names of metabolites and reactions. To facilitate integration with MetaNetX, we edited content of the original file for Recon 2M.2 in SBML format. We changed identifiers of metabolites to remove unnecessary prefixes and change the designation of the boundary compartment. We also changed identifiers or names of 104 metabolites and 3 compartments to correct errors and improve mapping to the MetaNetX name space. We imported this new version of Recon 2M.2 to MetaNetX, which matched information about reactions, metabolites, and compartments to its own records. Whereas Recon 2M.2 includes distinct entries for compartmental instances of metabolites, MetaNetX [16] consolidates information for chemically-identical metabolites. After reconciliation and integration to MetaNetX, we exported consensus, standard information about reactions, enzymes, metabolites, and compartments in text tables with tab delimiters. We derived our own version of the metabolic model from this information (**Table 1**).

We curated and enhanced information about metabolites in our model of human metabolism. We made 197 custom curations to information about metabolites, especially to correct and enhance references to external databases. We accessed information for all 114100 records about metabolites in version 4.0 of HMDB [17], file "hmdb_metabolites.xml" (4.2 Gigabytes). We matched the majority of metabolites in the model to records in HMDB, and derived names from these records. Also from records in HMDB we derived references to PubChem [18]. Table 1 describes the extent of curation and coverage of references for metabolites in the model of human metabolism.

We also curated and filtered information about reactions in our model of human metabolism. We made 102 custom curations to information about reactions, especially to clarify names on the basis of their references to genes [19]. We interpreted the behavior of reactions in either chemical conversion or compartmental transport of metabolites. We then included transport reactions in processes (metabolic pathways) that span multiple compartments and include matching metabolites and compartments with the reaction. We also filtered reactions to enhance the model's relevance to our analyses. The original Recon 2M.2 model [12] included many reactions involving the exchange of metabolites with the model's boundary and the extracellular compartment, the accumulation of biomass, and the assembly and degradation of proteins. While these reactions' are relevant to simulations of metabolic flux, they do not provide relevant context for interpretation of intracellular metabolomic measurements. We removed them from the model. Table 1 describes the extent of curation and filtration of reactions in the model of human metabolism. We used the final human metabolic model for further definition and analysis of metabolic networks both in MetaboNet [14] and DyMetaboNet [13].

Web application for definition and visual exploration of metabolic networks

We designed and developed the DyMetaboNet web application [13] for basic definition and exploration of human metabolic networks. We implemented the application's interface in the web document and its behavior in the JavaScript programming language. We used the Data-Driven Documents (D3) [73] library for JavaScript to represent dynamic information visually. The application runs in the user's internet browser independently of any server. When the user navigates in the internet browser to the Uniform Resource Locator (URL) of DyMetaboNet's host (https://tcameronwaller.github.io/dymetabonet/), all necessary source files and code download to the user's computer, and the entire application runs locally on the user's computer. The internet browser has a firewall to contain this information from web applications and thereby protect the client's computer. DyMetaboNet imports information about metabolites, reactions, compartments, and processes that MetaboNet [14] exports in a file in JavaScript Object Notation (JSON) format. From controls in its interface, DyMetaboNet defines custom networks by a similar method to MetaboNet [13]. Dynamic queries select subnetworks of interest from these custom networks using our own custom implementations of common algorithms for proximity (breadth-first search) and paths between two or more nodes (simple shortest paths) [74]. DyMetaboNet also exports tables of information about metabolites and reactions in these networks and subnetworks.

Definition of custom metabolic networks

We defined networks to represent human metabolism. We selected a representation as a directional, bipartite network with distinct types of nodes for reactions and metabolites (**Figure 1**). In this representation, nodes for metabolites only relate to each other through nodes for reactions, such that reactant metabolites have links to their reactions and product metabolites have links from their reactions. Reversible reactions define these links in both directions.

We defined metabolic networks to represent metabolism both with and without compartmentalization (**Figure 1A-B**). Our compartmental networks include distinct nodes to distinguish between chemically-identical metabolites and reactions that occur in separate cellular compartments. Many of these reactions do not mediate any chemical change between metabolites but instead facilitate transport of metabolites between separate compartments. Our non-compartmental representation is much more concise. We only include nodes for chemically-unique metabolites and reactions. Without compartments, many reactions are chemically-redundant, and we represent these redundant replicates by a single, consensus reaction. Also, reactions that mediate compartmental transport of metabolites are irrelevant without compartments, and we exclude these from the network.

We exert customizable criteria for reactions and metabolites to qualify for representation in the network. In our model of metabolism, reactions specify the compartments in which they occur, and they also specify metabolic processes to which they belong. Hence these compartments and processes define sets of reactions and metabolites, and the relevance of these sets depends on the context of experiments. Our procedure accommodates customizable lists of compartments and processes to apply as filters. Similarly, the relevance of individual reactions and metabolites depends on the context of experiments. Our procedure also accommodates customizable lists of reactions and metabolites to include or exclude from the network. By default, we exclude metabolite hubs from the network (**Figure 1C-D, Table S1**). To qualify for representation in the network, reactions must themselves not have designations for exclusion, and they must also belong to sets of compartments and processes that pass filters. Similarly, metabolites must participate in relevant reactions in order to be part of the network. After definition of nodes and links, we selected only the largest connected component from the network. We then converted the format of information about human metabolic networks for further analyses in NetworkX [69], and Cytoscape [22].

Analysis of custom metabolic networks

We applied algorithms and metrics from graph theory to describe our metabolic networks. Bipartite networks [43] such as ours require specific constraints. Where available, we selected implementations of appropriate algorithms in version 2.3 of NetworkX [69]. Where these were unavailable, we implemented our own tools in the MetaboNet package [14]. Several algorithms calculate metrics relative only to a single bipartite set of nodes, either metabolites or reactions. We specify this type of metric by the phrase "single-mode". For most single-mode metrics, we only report the values relative to metabolites (**Table 2**); however, complete metrics for all networks are available in an archive of MetaboNet's complete export data [15]. Furthermore, several al-

gorithms normalize metrics by comparison to their maximal possibility for a bipartite network with directional links and with identical counts of nodes in each of its bipartite sets. We specify this normalization by the phrase "comparison to maximum" or "comparison to maxima". Other algorithms normalize metrics by comparison to their mean across multiple simulations of random bipartite networks with directional links and identical counts of nodes in each of their bipartite sets. We specify this normalization by the phrase "comparison to random".

- To measure density, we used an algorithm from NetworkX [69] that normalizes the network's actual size by comparison to maximum.
- To measure the centralities of individual nodes, we used algorithms from NetworkX [69] that calculate single-mode degree and betweenness centralities and normalize these by comparison to maximum [75]. MetaboNet [14] calculates these centralities relative to the bipartite sets of nodes for both metabolites and reactions respectively. We used these centralities further to rank metabolites by a combination of their close (degree) and long-range (betweenness) influences in the metabolic networks [45].
- To measure centralization of the entire network, we implemented our own versions of algorithms that calculate single-mode degree and betweenness centralities and normalize these by comparison to maximum [76, 75]. MetaboNet [14] calculates these centralizations relative to the bipartite sets of nodes for both metabolites and reactions respectively.
- To measure cluster coefficients of individual nodes, we used an algorithm from NetworkX [69] that calculates single-mode coefficients [77].
- To measure mean cluster coefficient of the entire network, we used an algorithm from NetworkX [69] that calculates the mean of single-mode coefficients [77]. MetaboNet [14] calculates these mean cluster coefficients relative to the bipartite sets of nodes for both metabolites and reactions respectively.
- To measure the mean path length of the entire network, we implemented our own custom version of an algorithm that calculates the mean of lengths of shortest paths between all single-mode pairs of nodes [69].
- To measure the small-world coefficient of the entire network, we adapted the sigma coefficient [78] for a bipartite network. Our custom implementation of the sigma coefficient algorithm normalizes mean cluster coefficient and mean path length by comparison to random [69].
- To measure the degree assortativity coefficient of the entire network, we used algorithms from NetworkX [69]. We first projected the bipartite network to a directional, unipartite network relative to either metabolites or reactions, respectively. We then calculated the degree assortativity coefficient of each single-mode projection.

Processing of metabolomic measurements

We curated and processed public metabolomic measurements for general analyses. We accessed metabolomic measurements from records for projects and studies within the Metabolomics Workbench [41] (**Table S3**). From these records, we extracted information about pairs and experimental groups of samples, total identifiable and unidentifiable signals for each sample, and measurements of identifiable analytes for each sample. We selected conceptual case and control experimental groups of samples to use for dividend (numerator) and divisor (denominator) respectively (**Table S3**) in calculations of fold changes. We removed analytes with inadequate coverage of measurements. If multiple analytes represented the same chemical entity redundantly, we prioritized the analyte with the least relative variance (index of dispersion or variance-to-mean ratio) in its measurements for the control experimental group. We normalized measurements for each sample to the total sum of signals in that sample. After normalization, we calculated fold changes, base-2 logarithms of fold changes, and probabilities (p-values) between measurements for each analyte in samples from each experimental group. These calculations depended on whether a study's samples were in dependent pairs from the same patient. For pairs of dependent samples, we calculated the mean of base-2 logarithms of fold changes for measurements from each pair, and we calculated the p-value using a two-side t-test for dependent populations. For independent samples, we calculated the base-2 logarithm of the fold change between the means of measurements from each group, and we calculated the p-value using a two-side t-test for independent populations. Our subsequent analyses used the mean base-2 logarithm of fold change and the p-value to compare each analyte between experimental groups. We visualized these values in custom volcano plots that we implemented using version 3.1.1 of MatPlotLib [70, 71].

We integrated metabolomic measurements in metabolic networks for further analysis. Most analytes in Metabolomics Workbench [41] include references to PubChem [18], and we used these references to match analytes to metabolites in our metabolic model. We manually critiqued all matches between analytes and metabolites for accuracy.

Analysis of metabolomic measurements in metabolic sets

We performed metabolite set enrichment analysis using version 4.0 of MetaboAnalyst [46]. We organized metabolomic measurements in a format appropriate for export to MetaboAnalyst. For compatibility, it was necessary to prepare measurements from all studies as though samples were independent, without pairs. We specified not to use any of the normalization options in MetaboAnalyst. We tested for enrichment in MetaboAnalyst's default library of 99 metabolic sets [46], considering those with 2 or more members. For each study, we summarized the sets with the top 5 ranks by p-value (**Table S6**).

Integration and analysis of metabolomic measurements in metabolic network

We integrated metabolomic measurements from each study (**Table S3**) with our metabolic network and searched for interesting clusters. We used our non-compartmental metabolic network without hubs for analyses of metabolomic measurements. We matched analytes and measurements to metabolites by common references to PubChem [18]. We imported information about the network and measurements into version 3.7.1 of Cytoscape [22] and used version 3.2.1 of the jActiveModules application [54, 55] in Cytoscape to detect raw clusters of proximal metabolites with enrichment in p-values. We detected these raw clusters in sets of 25 at search depths of 2 links with overlap thresholds of 0.25, 0.50, and 0.75. On nodes for metabolites in these raw clusters we represented the base-2 logarithm fold change in bidirectional color saturation. We then searched these raw clusters prioritizing

those with 3 or fewer reactions in which the majority of metabolites had measurements, and in which proximal metabolites demonstrated both accumulation and depletion. From these raw clusters we curated final clusters of interest (**Figures 5, S1, S2, S3, S4**; **Table S4**; **Table S5**), excluding metabolites without measurements and including proximal metabolites with measurements that are biologically relevant. We curated names and confirmed accuracy of genes for all reactions in these final clusters. We also collected references to Entrez Gene [19] and UniProt [32] for these reactions. We summarized measurements and information about metabolites (**Table S4**) and reactions (**Table S5**) within these clusters.

Availability of source code and requirements

Curation of the human metabolic model; definition, analysis, and export of custom metabolic networks; processing metabolomic measurements and integration with metabolic networks.

- Project name: MetaboNet
- Project home page: https://github.com/tcameronwaller/metabonet
- Operating system(s): platform independent
- Programming language: Python 3
- Other requirements: SciPy, NumPy, NetworkX, MatPlotLib, WordCloud
- License: GNU General Public License version 3

Dynamic definition and visual exploration of metabolic networks.

- Project name: DyMetaboNet
- Project home page: https://github.com/tcameronwaller/dymetabonet
- Operating system(s): platform independent
- Programming language: JavaScript
- Other requirements: Data-Driven Documents (D3)
- License: GNU General Public License version 3

Availability of supporting data and materials

This article's analyses used v1.0.0 of MetaboNet [14] and v1.0.0 of DyMetaboNet [13]. The data for these analyses are available in record "3382296" of the Zenodo repository [15]. Snapshots of our code and other supporting data are available in the GigaScience repository GigaDB [79].

Declarations

List of abbreviations

- ABP: Adenosine 3',5'-bisphosphate
- ADP: Adenosine 5'-diphosphate
- AMP: Adenosine 5'-monophosphate
- ATP: Adenosine 5'-triphosphate
- BCAA: Branched Chain Amino Acid
- ChEBI: Chemical Entities of Biological Interest
- CMP: Cytidine 5'-monophosphate
- D3: Data-Driven Documents
- EC: Enzyme Commission
- FAD2+: Flavin adenine dinucleotide
- FADH2: Flavin adenine dinucleotide reduced
- GC: Gas chromatography
- GDP: Guanosine 5'-diphosphate
- HGNC: HUGO Gene Nomenclature Committee
- HHMI: Howard Hughes Medical Institute
- HMDB: Human Metabolome Database
- HUGO: Human Genome Organization
- JSON: JavaScript Object Notation
- KEGG: Kyoto Encyclopedia of Genes and Genomes
- LC: Liquid chromatography
- MS: Mass spectrometry
- NAD1+: Nicotinamide adenine dinucleotide
- NADH: Nicotinamide adenine dinucleotide reduced
- NADP1+: Nicotinamide adenine dinucleotide phosphate
- NADPH: Nicotinamide adenine dinucleotide phosphate reduced
- NCI: National Cancer Institute
- NIH: National Institutes of Health
- NMR: Nuclear Magnetic Resonance
- p53: Tumour suppressor protein 53
- RefSeq: Reference Sequence
- SBML: Systems Biology Markup Language
- TIGAR: Tumour suppressor protein 53-Induced Glycolysis and Apoptosis Regulator
- UDP: Uridine 5'-diphosphate
- URL: Uniform Resource Locator
- XML: Extensible Markup Language

Ethical Approval (optional)

Not applicable.

Consent for publication

Not applicable.

Competing Interests

The authors declare that they have no conflicting or competing interests.

Funding

T.C.W. and J.A.B. received support from the National Institutes of Health (NIH) Interdisciplinary Training Grant T32 Program in Computational Approaches to Diabetes and Metabolism Research, 1T32DK11096601 to Wendy W. Chapman and Simon J. Fisher. B.E.C. received support from the office of the Senior Vice President for University of Utah Health Sciences. T.C.W. and J.R. received support from the National Cancer Institute (NCI) grant CA228346 to J.R. J.R. is also an Investigator of the Howard Hughes Medical Institute (HHMI).

Author's Contributions

- **Conceptualization:** T.C.W., J.R.
- **Supervision:** T.C.W., J.A.B., A.L., B.E.C., J.R.
- **Project Administration:** T.C.W., J.A.B., A.L., B.E.C., J.R.
- **Investigation:** T.C.W.
- **Formal Analysis:** T.C.W.

- **Software:** T.C.W., J.A.B.
- **Methodology:** T.C.W.
- **Validation:** T.C.W.
- **Data Curation:** T.C.W.
- **Resources:** T.C.W., J.A.B., A.L., B.E.C., J.R.
- **Funding Acquisition:** T.C.W., J.A.B., A.L., B.E.C., J.R.
- **Writing – Original Draft Preparation:** T.C.W.
- **Writing – Review and Editing:** T.C.W., J.A.B., A.L., B.E.C., J.R.
- **Visualization:** T.C.W.

Acknowledgements

We acknowledge and thank Kathryn A. Waller for consultation on the visual design of DyMetaboNet; James E. Cox, Kevin G. Hicks, and Alex J. Bott for consultation on metabolomic technologies and research; and Sara L. Johnson for consultation on comparison of our analyses to standard practice.

Authors' information (optional)

Not applicable.

References

1. Haas R, Zelezniak A, Iacovacci J, Kamrad S, Townsend S, Ralser M. Designing and interpreting 'multi-omic' experiments that may change our understanding of biology. Current Opinion in Systems Biology 2017 Dec;6:37–45. https://linkinghub.elsevier.com/retrieve/pii/S2452310017300835.
2. Pavlova NN, Thompson CB. The Emerging Hallmarks of Cancer Metabolism. Cell Metabolism 2016 Jan;23(1):27–47.
3. Vander Heiden MG, DeBerardinis RJ. Understanding the Intersections between Metabolism and Cancer Biology. Cell 2017;168(4):657–669.
4. Hotamisligil GS. Inflammation, metaflammation and immunometabolic disorders. Nature 2017;542(7640):177–185.
5. Hu JX, Thomas CE, Brunak S. Network biology concepts in complex disease comorbidities. Nature Reviews Genetics 2016;17(10):615–629.
6. Kaushik AK, DeBerardinis RJ. Applications of metabolomics to study cancer metabolism. Biochimica Et Biophysica Acta Reviews on Cancer 2018 Aug;1870(1):2–14.
7. Ritchie MD, Holzinger ER, Li R, Pendergrass SA, Kim D. Methods of integrating data to uncover genotype-phenotype interactions. Nature Reviews Genetics 2015 Feb;16(2):85–97.
8. Karczewski KJ, Snyder MP. Integrative omics for health and disease. Nature Reviews Genetics 2018 May;19(5):299–310.
9. Barabási AL, Oltvai ZN. Network biology: understanding the cell's functional organization. Nature Reviews Genetics 2004 Feb;5(2):101–113.
10. Mitra K, Carvunis AR, Ramesh SK, Ideker T. Integrative approaches for finding modular structure in biological networks. Nature Reviews Genetics 2013 Oct;14(10):719–732.
11. Barupal DK, Fan S, Fiehn O. Integrating bioinformatics approaches for a comprehensive interpretation of metabolomics datasets. Current Opinion in Biotechnology 2018 Dec;54:1–9.
12. Ryu JY, Kim HU, Lee SY. Framework and resource for more than 11,000 gene-transcript-protein-reaction associations in human metabolism. Proceedings of the National Academy of Sciences of the United States of America 2017;114(45):E9740–E9749.
13. Waller TC, DyMetaboNet. GitHub; 2019. http://doi.org/10.5281/zenodo.3382315.
14. Waller TC, MetaboNet. GitHub; 2019. http://doi.org/10.5281/zenodo.3382317.
15. Waller TC, Berg JA, Compartment and Hub Definitions Tune Metabolic Networks for Metabolomic Interpretations. Zenodo; 2019. http://doi.org/10.5281/zenodo.3382296.
16. Moretti S, Martin O, Van Du Tran T, Bridge A, Morgat A, Pagni M. MetaNetX/MNXref–reconciliation of metabolites and biochemical reactions to bring together genome-scale metabolic networks. Nucleic Acids Research 2016 Jan;44(D1):D523–526.
17. Wishart DS, Feunang YD, Marcu A, Guo AC, Liang K, Vázquez-Fresno R, et al. HMDB 4.0: the human metabolome database for 2018. Nucleic Acids Research 2018 Jan;46(D1):D608–D617.
18. Kim S, Thiessen PA, Bolton EE, Chen J, Fu G, Gindulyte A, et al. PubChem Substance and Compound databases. Nucleic Acids Research 2016 Jan;44(D1):D1202–1213.
19. Brown GR, Hem V, Katz KS, Ovetsky M, Wallin C, Ermolaeva O, et al. Gene: a gene-centered information resource at NCBI. Nucleic Acids Research 2015 Jan;43(Database issue):D36–42.
20. Bairoch A. The ENZYME database in 2000. Nucleic Acids Research 2000 Jan;28(1):304–305.
21. Artimo P, Jonnalagedda M, Arnold K, Baratin D, Csardi G, de Castro E, et al. ExPASy: SIB bioinformatics resource portal. Nucleic Acids Research 2012 Jul;40(Web Server issue):W597–603.
22. Shannon P, Markiel A, Ozier O, Baliga NS, Wang JT, Ramage D, et al. Cytoscape: a software environment for integrated models of biomolecular interaction networks. Genome Research 2003 Nov;13(11):2498–2504.
23. Le Novère N. Quantitative and logic modelling of molecular and gene networks. Nature Reviews Genetics 2015 Mar;16(3):146–158.
24. O'Brien EJ, Monk JM, Palsson BO. Using Genome-scale Models to Predict Biological Capabilities. Cell 2015 May;161(5):971–987.
25. Hastings J, Owen G, Dekker A, Ennis M, Kale N, Muthukrishnan V, et al. ChEBI in 2016: Improved services and an expanding collection of metabolites. Nucleic Acids Research 2016 Jan;44(D1):D1214–1219.
26. Kanehisa M, Furumichi M, Tanabe M, Sato Y, Morishima K. KEGG: new perspectives on genomes, pathways, diseases and drugs. Nucleic Acids Research 2017;45(D1):D353–D361.
27. Caspi R, Billington R, Fulcher CA, Keseler IM, Kothari A, Krummenacker M, et al. The MetaCyc database of metabolic pathways and enzymes. Nucleic Acids Research 2018 Jan;46(D1):D633–D639.
28. Fabregat A, Jupe S, Matthews L, Sidiropoulos K, Gillespie M, Garapati P, et al. The Reactome Pathway Knowledgebase. Nucleic Acids Research 2018 Jan;46(D1):D649–D655.
29. Braschi B, Denny P, Gray K, Jones T, Seal R, Tweedie S, et al. Genenames.org: the HGNC and VGNC resources in 2019. Nucleic Acids Research 2018 Oct;.
30. O'Leary NA, Wright MW, Brister JR, Ciufo S, Haddad D, McVeigh R, et al. Reference sequence (RefSeq) database at NCBI: current status, taxonomic expansion, and functional annotation. Nucleic Acids Research 2016 Jan;44(D1):D733–745.
31. Zerbino DR, Achuthan P, Akanni W, Amode MR, Barrell D,

Bhai J, et al. Ensembl 2018. Nucleic Acids Research 2018 Jan;46(D1):D754–D761.
32. UniProt Consortium T. UniProt: the universal protein knowledgebase. Nucleic Acids Research 2018 Mar;46(5):2699.
33. Duarte NC, Becker SA, Jamshidi N, Thiele I, Mo ML, Vo TD, et al. Global reconstruction of the human metabolic network based on genomic and bibliomic data. Proceedings of the National Academy of Sciences of the United States of America 2007 Feb;104(6):1777–1782.
34. Hao T, Ma HW, Zhao XM, Goryanin I. Compartmentalization of the Edinburgh Human Metabolic Network. BMC bioinformatics 2010 Jul;11:393.
35. Thiele I, Swainston N, Fleming RMT, Hoppe A, Sahoo S, Aurich MK, et al. A community-driven global reconstruction of human metabolism. Nature Biotechnology 2013 May;31(5):419–425.
36. Mardinoglu A, Agren R, Kampf C, Asplund A, Uhlen M, Nielsen J. Genome-scale metabolic modelling of hepatocytes reveals serine deficiency in patients with non-alcoholic fatty liver disease. Nature Communications 2014;5:3083.
37. Swainston N, Smallbone K, Hefzi H, Dobson PD, Brewer J, Hanscho M, et al. Recon 2.2: from reconstruction to model of human metabolism. Metabolomics: Official Journal of the Metabolomic Society 2016;12:109.
38. King ZA, Lu J, Dräger A, Miller P, Federowicz S, Lerman JA, et al. BiGG Models: A platform for integrating, standardizing and sharing genome-scale models. Nucleic Acids Research 2016 Jan;44(D1):D515–522.
39. Cottret L, Frainay C, Chazalviel M, Cabanettes F, Gloaguen Y, Camenen E, et al. MetExplore: collaborative edition and exploration of metabolic networks. Nucleic Acids Research 2018 Jul;46(W1):W495–W502.
40. Hucka M, Finney A, Sauro HM, Bolouri H, Doyle JC, Kitano H, et al. The systems biology markup language (SBML): a medium for representation and exchange of biochemical network models. Bioinformatics (Oxford, England) 2003 Mar;19(4):524–531.
41. Sud M, Fahy E, Cotter D, Azam K, Vadivelu I, Burant C, et al. Metabolomics Workbench: An international repository for metabolomics data and metadata, metabolite standards, protocols, tutorials and training, and analysis tools. Nucleic Acids Research 2016 Jan;44(D1):D463–470.
42. Ryu JY, Kim HU, Lee SY, Framework and resource for more than 11,000 gene-transcript-protein-reaction associations in human metabolism. Zenodo; 2017. https://zenodo.org/record/583326.
43. Pavlopoulos GA, Kontou PI, Pavlopoulou A, Bouyioukos C, Markou E, Bagos PG. Bipartite graphs in systems biology and medicine: a survey of methods and applications. GigaScience 2018;7(4):1–31.
44. Alam MT, Olin-Sandoval V, Stincone A, Keller MA, Zelezniak A, Luisi BF, et al. The self-inhibitory nature of metabolic networks and its alleviation through compartmentalization. Nature Communications 2017;8:16018.
45. Liao H, Mariani MS, Medo M, Zhang YC, Zhou MY. Ranking in evolving complex networks. Physics Reports 2017 May;689:1–54. https://linkinghub.elsevier.com/retrieve/pii/S0370157317300935.
46. Chong J, Soufan O, Li C, Caraus I, Li S, Bourque G, et al. MetaboAnalyst 4.0: towards more transparent and integrative metabolomics analysis. Nucleic Acids Research 2018 Jul;46(W1):W486–W494.
47. Liesenfeld DB, Grapov D, Fahrmann JF, Salou M, Scherer D, Toth R, et al. Metabolomics and transcriptomics identify pathway differences between visceral and subcutaneous adipose tissue in colorectal cancer patients: the ColoCare study. The American Journal of Clinical Nutrition 2015 Aug;102(2):433–443.
48. Fiehn O, Project PR000058, Study ST000061. Metabolomics Workbench; 2014. https://doi.org/10.21228/M80018.
49. Wikoff WR, Grapov D, Fahrmann JF, DeFelice B, Rom WN, Pass HI, et al. Metabolomic markers of altered nucleotide metabolism in early stage adenocarcinoma. Cancer Prevention Research (Philadelphia, Pa) 2015 May;8(5):410–418.
50. Fiehn O, Project PR000305, Study ST000390. Metabolomics Workbench; 2010. https://doi.org/10.21228/M8PG66.
51. Bruinsma BG, Sridharan GV, Weeder PD, Avruch JH, Saeidi N, Özer S, et al. Metabolic profiling during ex vivo machine perfusion of the human liver. Scientific Reports 2016 Mar;6:22415.
52. Fiehn O, Project PR000322, Study ST000412. Metabolomics Workbench; 2016. https://doi.org/10.21228/M8V312.
53. Kachman M, Horowitz J, Project PR000599, Study ST000842. Metabolomics Workbench; 2017. https://doi.org/10.21228/M87Q3F.
54. Ideker T, Ozier O, Schwikowski B, Siegel AF. Discovering regulatory and signalling circuits in molecular interaction networks. Bioinformatics (Oxford, England) 2002;18 Suppl 1:S233–240.
55. Saito R, Smoot ME, Ono K, Ruscheinski J, Wang PL, Lotia S, et al. A travel guide to Cytoscape plugins. Nature Methods 2012 Nov;9(11):1069–1076.
56. García-Campos MA, Espinal-Enríquez J, Hernández-Lemus E. Pathway Analysis: State of the Art. Frontiers in Physiology 2015;6:383.
57. Yamada T, Bork P. Evolution of biomolecular networks: lessons from metabolic and protein interactions. Nature Reviews Molecular Cell Biology 2009 Nov;10(11):791–803.
58. Gottschling DE, Nyström T. The Upsides and Downsides of Organelle Interconnectivity. Cell 2017;169(1):24–34.
59. Hao D, Li C. The dichotomy in degree correlation of biological networks. PloS One 2011;6(12):e28322.
60. King ZA, Dräger A, Ebrahim A, Sonnenschein N, Lewis NE, Palsson BO. Escher: A Web Application for Building, Sharing, and Embedding Data-Rich Visualizations of Biological Pathways. PLoS computational biology 2015 Aug;11(8):e1004321.
61. Karnovsky A, Weymouth T, Hull T, Tarcea VG, Scardoni G, Laudanna C, et al. Metscape 2 bioinformatics tool for the analysis and visualization of metabolomics and gene expression data. Bioinformatics (Oxford, England) 2012 Feb;28(3):373–380.
62. Lindsley JE, Rutter J. Whence cometh the allosterome? Proceedings of the National Academy of Sciences of the United States of America 2006 Jul;103(28):10533–10535.
63. Reznik E, Christodoulou D, Goldford JE, Briars E, Sauer U, Segrè D, et al. Genome-Scale Architecture of Small Molecule Regulatory Networks and the Fundamental Trade-Off between Regulation and Enzymatic Activity. Cell Reports 2017 Sep;20(11):2666–2677.
64. Orsak T, Smith TL, Eckert D, Lindsley JE, Borges CR, Rutter J. Revealing the allosterome: systematic identification of metabolite-protein interactions. Biochemistry 2012 Jan;51(1):225–232.
65. Diether M, Sauer U. Towards detecting regulatory protein-metabolite interactions. Current Opinion in Microbiology 2017 Oct;39:16–23.

66. Piazza I, Kochanowski K, Cappelletti V, Fuhrer T, Noor E, Sauer U, et al. A Map of Protein-Metabolite Interactions Reveals Principles of Chemical Communication. Cell 2018;172(1-2):358–372.e23.
67. Jones E, Oliphant TE, Peterson P, SciPy: Open source scientific tools for Python; 2001. http://www.scipy.org/.
68. Oliphant TE. Guide to NumPy; 2015. OCLC: 1030608394.
69. Hagberg AA, Schult DA, Swart PJ. Exploring Network Structure, Dynamics, and Function using NetworkX. In: Varoquaux G, Vaught T, Millman J, editors. Proceedings of the 7th Python in Science Conference Pasadena, California; 2008. p. 11–15. http://conference.scipy.org/proceedings/SciPy2008/paper_2/.
70. Hunter JD. Matplotlib: A 2D Graphics Environment. Computing in Science & Engineering 2007;9(3):90–95. http://ieeexplore.ieee.org/document/4160265/.
71. Caswell TA, Droettboom M, Hunter J, Firing E, Lee A, Stansby D, et al., Matplotlib/Matplotlib V3.0.2. Zenodo; 2018. https://zenodo.org/record/1482099.
72. Mueller A, WordCloud for Python; 2018. https://amueller.github.io/word_cloud/.
73. Bostock M, Ogievetsky V, Heer J. D^3: Data-Driven Documents. IEEE transactions on visualization and computer graphics 2011 Dec;17(12):2301–2309.
74. Yen JY. Finding the K Shortest Loopless Paths in a Network. Management Science 1971 Jul;17(11):712–716. http://pubsonline.informs.org/doi/abs/10.1287/mnsc.17.11.712.
75. Borgatti SP, Halgin DS, Scott J, Carrington PJ, editors, Analyzing Affiliation Networks. 1 Oliver's Yard, 55 City Road, London EC1Y 1SP United Kingdom: SAGE Publications Ltd; 2014. http://methods.sagepub.com/book/the-sage-handbook-of-social-network-analysis/n28.xml.
76. Borgatti SP, Everett MG. Network analysis of 2-mode data. Social Networks 1997 Aug;19(3):243–269. http://linkinghub.elsevier.com/retrieve/pii/S0378873396003012.
77. Latapy M, Magnien C, Vecchio ND. Basic notions for the analysis of large two-mode networks. Social Networks 2008 Jan;30(1):31–48. http://linkinghub.elsevier.com/retrieve/pii/S0378873307000494.
78. Humphries MD, Gurney K. Network 'small-world-ness': a quantitative method for determining canonical network equivalence. PloS One 2008 Apr;3(4):e0002051.
79. Waller TC, Berg JA, Lex A, Chapman BE, Rutter J. Supporting data for "Compartment and Hub Definitions Tune Metabolic Networks for Metabolomic Interpretations". GigaScience Database 2019;http://dx.doi.org/10.5524/100659.

Response to reviewers

Click here to access/download
Supplementary Material
waller_response R1.pdf

Click here to access/download
Supplementary Material
article.tex

Click here to access/download
Supplementary Material
float.tex

Click here to access/download
Supplementary Material
gigascience-logo.pdf

Click here to access/download
Supplementary Material
main.tex

Click here to access/download
Supplementary Material
oup-contemporary.cls

Click here to access/download
Supplementary Material
oup.pdf

Click here to access/download
Supplementary Material
reference.bib

Click here to access/download
Supplementary Material
section.tex

Click here to access/download
Supplementary Material
vancouver-authoryear.bst

Click here to access/download
Supplementary Material
figure_study-2.pdf

Click here to access/download
Supplementary Material
figure_study-2_script.tex

Click here to access/download
Supplementary Material
figure_study-3.pdf

Click here to access/download
Supplementary Material
figure_study-3_script.tex

Click here to access/download
Supplementary Material
figure_study-4.pdf

Click here to access/download
Supplementary Material
figure_study-4_script.tex

Click here to access/download
Supplementary Material
figure_study-5.pdf

Click here to access/download
Supplementary Material
figure_study-5_script.tex

Click here to access/download
Supplementary Material
table_hubs.tex

Click here to access/download
Supplementary Material
table_metabolites.tex

Click here to access/download
Supplementary Material
table_metric.tex

Click here to access/download
Supplementary Material
table_reactions.tex

Click here to access/download
Supplementary Material
table_sets.tex

Click here to access/download
Supplementary Material
table_studies.tex
