# Supplementary material for: Compartment and hub definitions tune metabolic networks for metabolomic interpretations
Source: Gigascience. 2020 Jan 23;9(1):giz137. doi: 10.1093/gigascience/giz137 (PMC6977586; doi:10.1093/gigascience/giz137)
Supplement: giz137_Supplemental_File [file giz137_supplemental_file.pdf]

Supplement

Analysis of metabolomic measurements in sets

Metabolite set enrichment analysis is an accessible and high-throughput method to interpret metabolomic measurements within a biological context. This analytical strategy is a foundation of the widely-used platform, MetaboAnalyst [1]. We found that MetaboAnalyst's visual, step-wise interface on the web was accessible, convenient, and easy to use. Users upload tables of measurements for each metabolite in samples within each experimental group. MetaboAnalyst processes these measurements, compares experimental groups, and evaluates enrichment of metabolites in functionally-relevant sets. MetaboAnalyst's default set library includes ninety-nine sets of metabolites that participate in common metabolic processes [1]. We included this strategy in our retrospective analysis of metabolomic measurements as a comparison to a current standard.

Metabolite set enrichment analysis in MetaboAnalyst [1], demonstrated some limitations in the set enrichment strategy. In this analysis, several sets ranked highly as hits even when measurements were only available for a small fraction of their total metabolites. In the most extreme example for Study 2, a set of thirty-five metabolites had measurements for only one matching metabolite (**Table S4**). This set still ranked in the top five hits with a p-value less than 1E-06 (**Table S4**). Also in this analysis, a few metabolites dominated multiple metabolite sets that were top hits. For Studies 1-5, a single metabolite belonged to at least three of the top five sets (**Table S4**). For Studies 1, 2, 4, and 5, a single metabolite belonged to at least four of the top five sets (**Table S4**). For Studies 1, 2, and 4, all five top sets included the same metabolite (**Table S4**). This overlap between sets and the tendency for a small count of metabolites to dominate top hits risks over-interpretation of artifactual measurements and false positives.

Analysis of metabolomic measurements in network clusters

To detect clusters of measurements on our metabolic network, we applied general functionality from Cytoscape [2]. Cytoscape [2] required local installation, installation of the jActiveModules application [3, 4], and familiarization with many settings for both analyses and visualizations. Still, Cytoscape and jActiveModules provided a specific and consistent protocol to search for preliminary clusters of measurements. The jActiveModules algorithm [3] detects enrichment of p-values across multiple nodes in clusters across the network, and this combination is sensitive even to weak differences in measurements.

This cluster enrichment strategy identified individual genes, transcripts, and proteins that were candidates for differential regulation between experimental conditions (**Table S6**). While we used a general algorithm [3] to search for our initial clusters, a more specific algorithm might reasonably automate cluster detection and quantify confidence in each cluster [5]. A more specific algorithm might replicate our analysis by detecting clusters both by enrichment of p-values and by prioritizing clusters with both accumulation and depletion of analytes. Also, alternative search strategies might excel at finding broader, more systemic changes, such as patterns of depletion in similar categories of metabolites, or accumulation of many metabolites within a single compartment.

Integration and analysis of metabolomic measurements with our metabolic network discovered multiple unique clusters suggestive of specific perturbations at individual reactions. We matched analytes from metabolomic studies to nodes for metabolites in our metabolic network. To these nodes we integrated the fold changes and p-values that we previously calculated to compare the measurements of each metabolite between experimental groups in each study (**Table S3**). We applied the jActiveModules algorithm [3] in Cytoscape [2] to detect preliminary clusters with metabolites and reactions of interest. As this search algorithm and its clusters base on the general enrichment of p-values, we integrated fold changes for an additional dimension in the search. We searched these clusters for patterns of both accumulation and depletion in metabolites that related closely by proximal reactions (**Figures 5, S1, S2, S3, S4; Table S5**). These patterns implicated individual reactions in differential regulation between experimental groups (**Table S6**). We identified genes and proteins for enzymes in these reactions and consulted Entrez Gene [6] and UniProt [7] databases to contextualize their roles and regulation in metabolism (**Table S6**).

Here we describe some of the more interesting clusters we found. For the sake of brevity, we limit our discussion to clusters from Studies 1, 2, and 5. We also discuss potential hypotheses and interpretations of these clusters. Importantly, our exploratory analyses of these studies are preliminary and are most suitable for the generation of speculative hypotheses.

Study 1: Cancerous versus Normal Lung

Many types of cancer alter metabolic flux to enhance macromolecular biosynthesis and thereby sustain cellular proliferation in tumors [8, 9]. Tumors also often experience microenvironments of hypoxia [8]. Study 1 (**Table S3**, **Figure 5**) collected samples of early-stage adenocarcinoma tumors and adjacent nonmalignant tissue from the lungs of thirty-nine human patients who were either current or former smokers [10]. The publication of Study 1 emphasized several observations from metabolomic measurements on cancerous lung relative to normal lung [10]. There was evidence of enhanced synthesis of nucleotides and increased response to reactive oxygen species along with surprisingly sparse synthesis of polyamines [10].

Cluster 1: Glycerate kinase. Study 1, Cluster 1 (**Figure 5C**) suggests that Glycerate kinase has differential activity in cancerous relative to normal lung, and this difference might partly sustain the synthesis of serine. Relative to normal lung, cancerous lung had accumulation of glyceric acid (p-value: **4.002E-03**) and depletion of 3-phosphoglyceric acid (p-value: **2.599E-03**) (**Figure 5C**, **Table S5**). 3-Phosphoglyceric acid is the product of dephosphorylation of 1,3-diphosphoglyceric acid by the enzyme Phosphoglycerate kinase in the Glycolysis metabolic pathway. 3-Phosphoglyceric acid is an allosteric inhibitor of 6-Phosphogluconate dehydrogenase [11], which has a role in the Pentose Phosphate metabolic pathway [12]. 3-Phosphoglyceric acid also supplies the main route for synthesis of serine through the enzyme 3-Phosphoglycerate dehydrogenase [13, 8]. Conversely, glyceric acid is a major product of the degradation of serine [13]. Hence, the enzyme Glycerate kinase in this cluster occupies an intersection of dynamic metabolic processes that shift to sustain cancerous metabolism [11, 12, 13, 14]. The original publication of this study acknowledged accumulation of metabolites in the Pentose Phosphate Pathway but did not emphasize Glycerate kinase as a candidate enzyme of interest [10]. The metabolite set "Glycine and Serine Metabolism" most closely represents this

cluster, and this set ranked 38th in metabolite set enrichment analysis.

Bisphosphoglycerate mutase might also be more active in cancerous than normal lung to enhance oxygenation or mitigate oxidative stress in tumors. 3-Phosphoglyceric acid is also the product of dephosphorylation of 2,3-diphosphoglyceric acid by Bisphosphoglycerate mutase [14]. Within red blood cells, 2,3-diphosphoglyceric acid binds to Hemoglobin and regulates its affinity for and release of dioxygen [15]. Placental cells also produce and release 2,3-diphosphoglyceric acid to promote the exchange of dioxygen between maternal and fetal blood [15]. By increasing activity of Bisphosphoglycerate mutase, cancerous lung might divert some 1,3-diphosphoglyceric acid away from Glycolysis to produce 2,3-diphosphoglyceric acid and thereby partially remedy the hypoxia in lung tumors [16]. Even minor variations in expression of Bisphosphoglycerate mutase seem to be relevant [14], and a preliminary search of transcriptomic data sets [17] demonstrated over-expression in pancreatic [18, 19] and thyroid [20, 21] cancers. Furthermore, the Tumour suppressor protein 53 (p53)-Induced Glycolysis and Apoptosis Regulator (TIGAR) resists cancerous metabolism [22] in part by degrading 2,3-diphosphoglyceric acid [23]. In tumors, increasing activity of Bisphosphoglycerate mutase and decreasing activity of TIGAR might benefit cancerous metabolism either by enhancing dioxygen delivery or by mitigating oxidative stress [24].

Cluster 2: Spermidine synthase. Study 1, Cluster 2 (**Figure 5D**) suggests that Spermidine synthase has differential activity in cancerous relative to normal lung, and this difference might allow proximal ornithine to supply synthesis of proline [10]. Relative to normal lung, cancerous lung had depletion of citrulline (p-value: **6.918E-04**), ornithine (p-value: **1.011E-05**), and spermidine (p-value: **2.531E-03**) (**Figure 5D**, **Table S5**). There was also accumulation of 5'-methylthioadenosine (p-value: **2.242E-03**) and adenine (p-value: **3.919E-04**) (**Figure 5D**, **Table S5**). Spermidine synthase in this cluster converts putrescine to spermidine and experiences inhibition from its product 5'-methylthioadenosine [25]. Accumulation of both 5'-methylthioadenosine and its degradation product adenine might indicate less activity in Methylthioadenosine phosphorylase and purine salvage [10]. The polyamines putrescine, spermidine, and spermine are abundant stabilizers of cell growth and survival, and many types of cancerous cells enhance synthesis of polyamines to facilitate proliferation [26, 8, 25]. The surprising depletion of spermidine in cancerous lung might be due to inhibition of Spermidine synthase by 5'-methylthioadenosine, and this decrease in activity might also allow ornithine to supply proline synthesis rather than polyamine synthesis [10]. Furthermore, inflammation of lung tissue, such as by smoking, enhances degradation of polyamines, and this response itself can favor carcinogenesis [26, 25]. The original publication of this study acknowledged accumulation of 5'-methylthioadenosine and depletion of both spermidine and ornithine [10]. It also emphasized Methylthioadenosine phosphorylase as a candidate enzyme of interest, but it did not emphasize Spermidine synthase as a candidate enzyme of interest [10]. The metabolite set "Spermidine and Spermine Biosynthesis" most closely represents this cluster, and this set ranked 33rd in metabolite set enrichment analysis.

Cluster 3: Uridine phosphorylase. Study 1, Cluster 3 (**Figure 5E**) suggests that Uridine phosphorylase has differential activity in cancerous relative to normal lung, and this difference might support the salvage of pyrimidines for proliferation. Relative to normal lung, cancerous lung had depletion of uridine (p-value: **4.385E-03**) and accumulation of both uracil (p-value: **1.057E-02**) and dihydrouracil (p-value: **1.449E-06**) (**Figure 5E**, **Table S5**). Uridine phosphorylase in this cluster interconverts uridine to uracil either for pyrimidine degradation or salvage [27]. Cancer cells require nucleotides for growth and proliferation [8, 9], and they must obtain these either by synthesis or salvage. Inhibition of pyrimidine synthesis by the uracil analogue 5-fluorouracil has been an effective chemotherapeutic strategy for decades [28]. The original publication of this study acknowledged accumulation of both uracil and dihydrouracil and emphasized Dihydropyrimidine dehydrogenase as a candidate enzyme of interest, but it did not emphasize Uridine phosphorylase as a candidate enzyme of interest [10]. The metabolite set "Pyrimidine Metabolism" most closely represents this cluster, and this set ranked 40th in metabolite set enrichment analysis.

Study 2: Visceral versus Subcutaneous Adipose

Visceral and subcutaneous white adipose tissues differ both in their roles in metabolism and in their contributions to morbidity in obesity [29]. Visceral and subcutaneous fat depots develop from distinct cellular lineages and grow in separate parts of the body [30, 31, 29]. Whereas subcutaneous adipose grows between the skin and muscle, visceral adipose grows within the abdominal cavity around internal organs. These fat depots also contribute differently to the morbidity of obesity, with visceral adipose promoting more inflammation and also imparting greater risk for metabolic syndrome, including diabetes, cardiovascular disease, and even cancer [30, 31, 29]. Study 2 (**Table S3**, **Figure S1**) collected samples of visceral and subcutaneous adipose tissues from fifty-nine human patients during surgery for colon cancer [32]. The publication of Study 2 emphasized several observations from integration of metabolomic and transcriptomic measurements on visceral adipose relative to subcutaneous adipose [32]. These observations were consistent with a primary role of visceral adipose as a metabolically-active endocrine organ and a primary role of subcutaneous adipose as a metabolically-dormant storage organ [32].

Cluster 1: Glucosidase alpha. Study 2, Cluster 1 (**Figure S1C**) suggests that Glucosidase alpha has differential activity in visceral relative to subcutaneous adipose, and this difference might be a selective therapeutic target in diabetes. Relative to subcutaneous adipose, visceral adipose had depletion of glucose (p-value: **2.557E-02**) and accumulation of both maltotriose (p-value: **1.229E-03**) and maltose (p-value: **1.942E-06**) (**Figure S1C**, **Table S5**). Glucosidase alpha in this cluster degrades polysaccharides to release glucose. Its activity is especially important to degrade complex sugars in the intestine, and it also functions in liver and muscle tissues to degrade glycogen and release glucose. A class of anti-diabetic drugs inhibit intestinal Glucosidase alpha to moderate the postprandial (after meal) absorption of glucose [31]. One member of this class of inhibitors of Glucosidase alpha, Miglitol, also moderates metabolic syndrome in obesity, partly by direct influence on both brown and white adipose tissues [33]. Visceral adipose absorbs more glucose than does subcutaneous adipose [30], and if the activity of Glucosidase alpha differs between visceral and subcutaneous white adipose as this cluster suggests, then Miglitol might also influence these depots selectively. The original publication of this study acknowledged depletion of glucose but did not emphasize Glucosidase alpha as a candidate enzyme of interest [32]. The metabolite set "Glycolysis" most closely represents this cluster, and this set ranked 10th in metabo-

lite set enrichment analysis.

Cluster 2: Branched-chain amino acid transaminase. Study 2, Cluster 2 (**Figure S1D**) suggests that Branched-chain amino acid (BCAA) transaminase has differential activity in visceral relative to subcutaneous adipose, and this difference might contribute to morbidity in obesity. Relative to subcutaneous adipose, visceral adipose had accumulation of all three BCAAs, leucine (p-value: **5.915E-06**), isoleucine (p-value: **5.240E-05**), and valine (p-value: **3.764E-04**) (**Figure S1D**, **Table S5**). There was also a slight depletion of ketoleucine (p-value: **6.385E-02**), the initial product of leucine degradation (**Figure S1D**, **Table S5**). The enzyme BCAA transaminase in this cluster catalyzes the first reaction in degradation of the BCAAs. Metabolic syndrome in obesity correlates with BCAA accumulation, partly attributable to a decrease in the expression of enzymes for BCAA degradation in visceral adipose tissue [34, 35, 36]. BCAAs influence the master nutrient sensor, Mammalian Target of Rapamycin (mTOR), which in turn regulates anabolism and catabolism of glucose and lipids [37]. The original publication of this study acknowledged accumulation of most amino acids but did not acknowledge accumulation of BCAAs specifically or emphasize BCAA transaminase as a candidate enzyme of interest [32]. The metabolite set "Valine, Leucine and Isoleucine Degradation" most closely represents this cluster, and this set ranked 71st in metabolite set enrichment analysis.

Cluster 3: Arginase. Study 2, Cluster 3 (**Figure S1E**) suggests that Arginase has differential activity in visceral relative to subcutaneous adipose, and this difference might exacerbate inflammation, oxidative stress, and insulin resistance. Relative to subcutaneous adipose, visceral adipose had depletion of urea (p-value: **2.795E-02**) and accumulation of both ornithine (p-value: **2.859E-05**) and putrescine (p-value: **1.900E-02**) (**Figure S1E**, **Table S5**). Arginase in this cluster converts arginine to urea and ornithine in the last reaction of the Urea Cycle [38]. Urea is a waste product to eliminate amines from the body, while ornithine supplies synthesis of proline and polyamines for cellular proliferation [38]. Arginase's activity also reduces the availability of arginine for synthesis of nitric oxide by Nitric oxide synthase [38]. Via both polyamines and nitric oxide, Arginase correlates with inflammation and oxidative stress [38]. Inhibition of Arginase ameliorates inflammation and insulin resistance in diabetes and obesity [38]. The original publication of this study did not emphasize Arginase as a candidate enzyme of interest [32]. The metabolite set "Urea Cycle" most closely represents this cluster, and this set ranked 23rd in metabolite set enrichment analysis.

Study 5: Obese versus Post-Exercise Muscle

Obesity is a major detriment to public health, and exercise is a life-style behavior that can mitigate this and other disease conditions profoundly [39]. Study 5 (**Table S3**, **Figure S4**) collected samples of muscle from ten obese human patients both before and after three months of a mild exercise program [40]. We are unaware of a publication on this study. Surprisingly, we found clusters for Study 5, which had weak fold changes and p-values for all but a few analytes.

Cluster 1: Malic enzyme. Study 5, Cluster 1 (**Figure S4C**) suggests that Malic enzyme has differential activity in obese muscle relative to muscle after exercise, and this difference might balance metabolism in response to slight hypoxia. Relative to muscle in obese patients before exercise, muscle in obese patients after exercise had slight accumulation of fumaric acid (p-value: **5.084E-02**) and slight depletion of pyruvate (p-value: **5.513E-02**) (**Figure S4C**, **Table S5**). These metabolites both participate in reactions with L-malic acid, which did not change appreciably (p-value: **8.337E-01**). Malic enzyme in this cluster converts malic acid to pyruvic acid, with various isozymes in the Cytosol and Mitochondrion. Malic enzyme cooperates with Pyruvate dehydrogenase and the Malate-Aspartate Shuttle as a metabolic bridge between Glycolysis in the Cytosol and the Citric Acid Cycle in the Mitochondrion. This bridge balances anapleurotic demands for materials and also balances reductive and oxidative nicotinamide adenine dinucleotides for both anabolic and catabolic processes [41, 42, 43]. Interestingly, fumaric acid allosterically activates Malic enzyme in the Mitochondrion [44]. During exercise, muscles might be slightly hypoxic, causing a stall in the Citric Acid Cycle and an imbalance in reduced and oxidized nicotinamide adenine dinucleotides. Malic enzyme might help to restore balance [41, 42, 43]. The metabolite set "Citric Acid Cycle" most closely represents this cluster, and this set ranked 42nd in metabolite set enrichment analysis.

Cluster 2: Xanthine dehydrogenase. Study 5, Cluster 2 (**Figure S4D**) suggests that Xanthine dehydrogenase has differential activity in obese muscle relative to muscle after exercise, and this difference might facilitate autophagy and moderate oxidative stress. Relative to muscle in obese patients before exercise, muscle in obese patients after exercise had accumulation of uric acid (p-value: **3.688E-02**) but no appreciable change in either xanthine (p-value: **3.144E-01**) or hypoxanthine (p-value: **1.777E-01**) (**Figure S4D**, **Table S5**). Xanthine dehydrogenase in this cluster catalyzes multiple reactions in the degradation of purines, of which uric acid is the terminal product [45]. In skeletal muscle, exercise enhances both the production of reactive oxygen species and the rate of autophagy, and both of these metabolic responses are important for muscle's adaptation to exercise [46, 47]. Xanthine dehydrogenase is likely to have a role in autophagy, and uric acid has complex contributions to oxidative stress [45]. The metabolite set "Purine Metabolism" most closely represents this cluster, and this set ranked 66th in metabolite set enrichment analysis.

Cluster 3: Histidine decarboxylase. Study 5, Cluster 3 (**Figure S4E**) suggests that Histidine decarboxylase has differential activity in obese muscle relative to muscle after exercise, and this difference might facilitate signalling for muscle recovery. Relative to muscle in obese patients before exercise, muscle in obese patients after exercise had depletion of histidine (p-value: **4.395E-02**) but no appreciable change in 1-methylhistamine (p-value: **1.348E-01**) (**Figure S4E**, **Table S5**). Histamine did not change appreciably. Histidine decarboxylase catalyzes synthesis of histamine from histidine, and Histamine N-methyltransferase catalyzes its degradation to 1-methylhistamine [48]. This cluster might indicate an enhancement in synthesis of histamine and its release from the cell. In skeletal muscle, exercise promotes the synthesis of histamine, where it acts as a local paracrine signal to promote blood circulation, delivery of glucose, and inflammation [48]. The metabolite set "Histidine Metabolism" most closely represents this cluster, and this set ranked 62nd in metabolite set enrichment analysis.

Table 1. Definition of metabolite hubs.

Name	Non-Compartmental Degree	Compartmental Degree
Proton	1494	1871
Water	1070	1240
Dioxygen	270	300
Phosphate	189	290
Diphosphate	126	183
Carbon dioxide	123	160
Sulfate	109	131
Hydrogen peroxide	107	132
Ammonium	68	84
Sulfite	7	24
Sodium	0	20
Hydrogen carbonate	0	0
Hydroxide	0	0

Name	Non-Compartmental Degree	Compartmental Degree
Coenzyme-A	473	593
Nicotinamide adenine dinucleotide (NAD1+)	349	421
Nicotinamide adenine dinucleotide reduced (NADH)	345	405
Nicotinamide adenine dinucleotide phosphate (NADP1+)	284	316
Nicotinamide adenine dinucleotide phosphate reduced (NADPH)	282	314
Acetyl coenzyme-A	169	215
(R)-Carnitine	149	276
Flavin adenine dinucleotide (FAD2+)	100	112
Flavin adenine dinucleotide reduced (FADH2)	98	106
Adenosine 5'-triphosphate (ATP)	245	392
Adenosine 5'-diphosphate (ADP)	167	280
Uridine 5'-diphosphate (UDP)	164	191
Adenosine 5'-monophosphate (AMP)	94	137
Cytidine 5'-monophosphate (CMP)	59	83
Adenosine 3',5'-bisphosphate (ABP)	55	57
Guanosine 5'-diphosphate (GDP)	52	75

Names and degrees of metabolite hubs for exclusion from metabolic networks. The degree of a metabolite's node is the count of links to reactions in which it participates as reactant or product. A metabolite's non-compartmental degree is the degree of its node in the non-compartmental network. A metabolite's compartmental degree is the cumulative degree across all nodes for its instances in the compartmental network. Metabolites on top are in Category 1. Metabolites on bottom are in Category 2.

Table 2. Definition of network metrics.

Name	Definition
**Order:**	Order is the count of nodes in a network. Bipartite networks have distinct orders for each of their two sets of nodes.
**Size:**	Size is the count of links between nodes in a network. Bipartite networks only have links between nodes of different types.
**Density:**	Density is proportional to a network's size normalized to the maximal size possible in a network of comparable order. Networks with greater density have more interconnections, more links per nodes.
**Centrality:**	Individual nodes within a network have centralities. Degree centrality relates to a node's close-range influence, and it is proportional to the count of links that connect to the node. Betweenness centrality relates to a node's long-range influence. It is proportional to the count of shortest paths between all pairs of other nodes in a network that pass through the focal node. Together, degree and betweenness centralities are useful to rank or prioritize nodes in a network [49].
**Centralization:**	Centralization is the extent to which a network has a single, central node of maximal centrality, surrounded by all other nodes of minimal centrality. The star network defines maximal centralization.
**Cluster coefficient:**	Each of a network's nodes has a cluster coefficient to describe the local density of connections proximal to individual nodes.
**Mean cluster coefficient:**	A network has a mean cluster coefficient across all of its nodes.
**Mean shortest path:**	A network has a mean length of shortest paths between all pairs of nodes. This mean path length indicates the efficiency of communication, or the pervasiveness of signals.
**Small-world coefficient:**	Like random networks, small-world networks have small mean path lengths that scale proportionately to the natural logarithm of their orders [50]. However, unlike random networks, small-world networks have large mean cluster coefficients [50]. The sigma small-world coefficient [50] measures the small-world character of a network by comparing both mean cluster coefficient and mean path length to random bipartite networks with identical orders and sizes. A value of the sigma coefficient greater than 1 suggests that a network is small-world.
**Assortativity:**	Assortativity is the tendency of nodes within a network to connect to other nodes that are similar by some attribute. Degree assortativity describes the tendency for nodes to connect to other nodes with similar degrees. The assortativity coefficient is a type of correlation coefficient.

Common network metrics applied to describe custom metabolic networks. Special considerations are necessary to adapt these metrics to bipartite networks [51].

Table 3. Curation of metabolomic measurements.

Study	Tissue	Group, Dividend	Group, Divisor	Pairs	Analytes	Metabolites	Project	Study	Reference
1	Lung	Tumor	Normal	True	177	119 (67.23%)	PR000305	ST000390	[10, 52]
2	Adipose	Viscera	Subcutane	True	132	91 (68.94%)	PR000058	ST000061	[32, 53]
3	Liver	Ischemia	Normal	False	151	108 (71.52%)	PR000322	ST000412	[54, 55]
4	Liver	Steatosis	Normal	False	151	108 (71.52%)	PR000322	ST000412	[54, 55]
5	Muscle	Exercise	Obese	True	125	73 (58.40%)	PR000599	ST000842	[40]

All studies use tissues from people of species Homo sapiens. Fold change calculations represent quotients of measurements from dividend (numerator) groups to divisor (denominator) groups. Information from all studies is accessible on Metabolomics Workbench [56] in specific records for each project and study.

Table 4. Metabolite Set Enrichment Analysis.

Study 1	Name	P-Value	Total	Hits	Metabolites
-	Lysine Degradation	3.961E-06	30	6	L-Glutamic acid, L-Lysine, Oxoglutaric acid, Aminoadipic acid, L-Pipecolic acid, FAD
-	Beta-Alanine Metabolism	8.478E-05	34	8	Beta-Alanine, Dihydrouracil, L-Glutamic acid, L-Histidine, L-Aspartic acid, Oxoglutaric acid, Uracil, FAD
-	Nicotinate and Nicotinamide Metabolism	8.569E-05	37	6	Adenosine monophosphate, L-Glutamic acid, L-Glutamine, FAD, Niacinamide, Phosphoric acid
-	Cysteine Metabolism	8.840E-05	26	4	Adenosine monophosphate, L-Glutamic acid, Oxoglutaric acid, Phosphoric acid
-	Warburg Effect	8.955E-05	58	12	Citric acid, Fructose 6-phosphate, Fumaric acid, L-Glutamic acid, L-Malic acid, Oxoglutaric acid, Succinic acid, L-Glutamine, 3-Phosphoglyceric acid, FAD, Glucose 6-phosphate, Phosphoric acid
**Study 2**	**Name**	**P-Value**	**Total**	**Hits**	**Metabolites**
-	Fatty Acid Biosynthesis	8.694E-07	35	6	Palmitic acid, 3-Hydroxybutyric acid, Caprylic acid, Capric acid, Dodecanoic acid, Myristic acid
-	Fatty Acid Elongation in Mitochondria	9.679E-07	35	1	Palmitic acid
-	Fatty Acid Metabolism	9.691E-07	43	3	Adenosine monophosphate, Palmitic acid, Pyrophosphate
-	Steroid Biosynthesis	9.696E-07	48	2	Palmitic acid, Pyrophosphate
-	Bile Acid Biosynthesis	1.054E-06	65	4	Glycine, Palmitic acid, Pyrophosphate, Taurine
**Study 3**	**Name**	**P-Value**	**Total**	**Hits**	**Metabolites**
-	Gluconeogenesis	1.030E-30	35	6	Oxoglutaric acid, Beta-D-Glucose, Malic acid, 3-Phosphoglyceric acid, Glucose 6-phosphate, Phosphoric acid
-	Trehalose Degradation	1.032E-30	11	1	Beta-D-Glucose
-	Glycolysis	1.034E-30	25	4	Beta-D-Glucose, 3-Phosphoglyceric acid, Glucose 6-phosphate, Phosphoric acid
-	Nucleotide Sugars Metabolism	1.293E-11	20	2	Uridine diphosphate glucuronic acid, Glucose 6-phosphate
-	Starch and Sucrose Metabolism	1.404E-11	31	6	Sucrose, D-Fructose, 3-Phosphoglyceric acid, Uridine diphosphate glucuronic acid, Glucose 6-phosphate, Beta-D-Fructose 6-phosphate
**Study 4**	**Name**	**P-Value**	**Total**	**Hits**	**Metabolites**
-	Butyrate Metabolism	1.830E-12	19	2	Adenosine monophosphate, Succinic acid
-	Mitochondrial Beta-Oxidation of Medium Chain Saturated Fatty Acids	1.217E-10	27	2	Adenosine monophosphate, Dodecanoic acid
-	Riboflavin Metabolism	1.226E-10	20	1	Adenosine monophosphate
-	Thiamine Metabolism	1.226E-10	9	1	Adenosine monophosphate
-	Ethanol Degradation	1.226E-10	19	1	Adenosine monophosphate
**Study 5**	**Name**	**P-Value**	**Total**	**Hits**	**Metabolites**
-	Spermidine and Spermine Biosynthesis	1.430E-02	18	1	L-Methionine
-	Methionine Metabolism	1.430E-02	43	3	L-Methionine, NAD, S-Adenosylhomocysteine
-	Betaine Metabolism	1.430E-02	21	3	L-Methionine, NAD, S-Adenosylhomocysteine
-	Glycine and Serine Metabolism	1.988E-02	59	7	Creatine, Glyceric acid, Oxoglutaric acid, Pyruvic acid, L-Methionine, NAD, S-Adenosylhomocysteine
-	Phospholipid Biosynthesis	4.631E-02	29	3	NAD, DG(16:0/16:0/0:0), LysoPC(16:0)

Metabolite set enrichment analysis in MetaboAnalyst [1]. Top 5 ranking sets for each study ranked by p-value. Column "Total" reports the cardinality or total count of metabolites in each set. Column "Hits" reports the count of metabolites from the set with measurements.

Table 5. Cluster Metabolites.

Study	Cluster	Analyte	Log-2 Fold	P-Value
1	1	Maltotriose	4.978E-01	1.229E-03
1	1	D-Glucose	-7.350E-01	2.557E-02
1	1	D-Maltose	1.679E+00	1.942E-06
1	2	L-Leucine	8.110E-01	5.915E-06
1	2	Ketoleucine	-1.997E-01	6.385E-02
1	2	L-Isoleucine	5.929E-01	5.240E-05
1	2	L-Valine	5.378E-01	3.764E-04
1	3	Citrulline	1.910E-01	1.960E-02
1	3	Ornithine	5.311E-01	2.859E-05
1	3	Putrescine	4.528E-01	1.900E-02
1	3	Urea	-2.626E-01	2.795E-02
1	3	Spermidine	1.773E-01	8.122E-02
2	1	Glyceric acid	3.996E-01	4.002E-03
2	1	3-Phosphoglyceric acid	-1.057E+00	2.599E-03
2	2	Citrulline	-4.962E-01	6.918E-04
2	2	Ornithine	-8.687E-01	1.011E-05
2	2	Putrescine	9.096E-02	4.030E-01
2	2	Spermidine	-5.751E-01	2.531E-03
2	2	5'-Methylthioadenosine	7.321E-01	2.242E-03
2	2	Adenine	4.932E-01	3.919E-04
2	3	Dihydrouracil	1.122E+00	1.449E-06
2	3	Uracil	5.588E-01	1.057E-02
2	3	Uridine	-8.510E-01	4.385E-03
3	1	Uridine diphosphate-N-acetylglucosamine	-7.965E-01	4.822E-05
3	1	Uridine 5'-monophosphate	9.399E-01	6.016E-03
3	2	Guanosine	1.626E+00	1.066E-06
3	2	Xanthine	-3.705E-01	2.079E-02
3	2	Hypoxanthine	1.420E+00	2.893E-04
3	2	Xanthosine	-1.045E+00	3.631E-08
3	2	Uric acid	-3.008E+00	1.205E-25
3	3	Glyceric acid	1.389E+00	4.517E-04
3	3	Glycerol	-1.741E+00	4.139E-10
3	3	Glycerol 3-phosphate	-2.083E-01	2.727E-01
4	1	L-Serine	7.594E-01	2.077E-02
4	1	L-Alanine	-5.580E-01	4.062E-03
4	1	Glycine	-6.363E-01	9.534E-03
4	2	Glyceric acid	2.284E+00	3.472E-03
4	2	Glycerol	-1.671E+00	5.360E-05
4	2	Glycerol 3-phosphate	-2.033E+00	6.463E-09
4	3	Guanosine	1.745E+00	1.674E-03
4	3	Xanthine	-8.043E-01	9.214E-04
4	3	Hypoxanthine	9.819E-01	1.233E-03
4	3	Xanthosine	-9.010E-01	2.006E-05
4	3	Uric acid	-1.974E+00	1.574E-09
5	1	Fumaric acid	7.357E-01	5.084E-02
5	1	L-Malic acid	-8.227E-02	8.337E-01
5	1	Pyruvic acid	-7.935E-01	5.513E-02
5	2	Hypoxanthine	-3.013E-01	1.777E-01
5	2	Xanthine	-1.462E-01	3.144E-01
5	2	Uric acid	2.562E+00	3.688E-02
5	3	L-Histidine	-3.593E-01	4.395E-02
5	3	Histamine	-3.444E-02	7.294E-01
5	3	1-Methylhistamine	2.873E-01	1.348E-01
5	3	S-Adenosylhomocysteine	4.790E-01	6.171E-02

Differential abundance in metabolomic measurements of metabolites in or relevant to clusters.

Table 6. Cluster Reactions.

Study 1	Cluster	Reaction	Gene Identifiers	Protein Identifiers
–	1	**Glucosidase alpha**	2548, 2595	P10253, Q8TET4
–	2	**Branched chain amino acid transaminase**	586, 587	
–	3	Ornithine carbamoyltransferase	5009	P00480
–	3	Ornithine decarboxylase	4953	P11926
–	3	**Arginase**	383, 384	P05089, P78540
–	3	Agmatinase	79814	Q9BSE5
–	3	Spermidine synthase	6723	P19623
**Study 2**	**Cluster**	**Reaction**	**Gene Identifiers**	**Protein Identifiers**
–	1	**Glycerate kinase**	132158	Q8IVS8
–	1	Phosphoglycerate mutase	669, 5223, 5224	P07738
–	2	Ornithine carbamoyltransferase	5009	P00480
–	2	Ornithine decarboxylase	4953	P11926
–	2	**Spermidine synthase**	6723	P19623
–	2	Methylthioadenosine phosphorylase	4507	Q13126
–	3	Dihydropyrimidine dehydrogenase	1806	
–	3	**Uridine phosphorylase**	7378, 151531	
**Study 3**	**Cluster**	**Reaction**	**Gene Identifiers**	**Protein Identifiers**
–	1	Dolichyl-phosphate N-acetyl-glucosamine-phospho-transferase	1798	
–	2	Purine nucleoside phosphorylase	4860	
–	2	Guanine deaminase	9615	
–	2	Xanthine dehydrogenase	7498	P47989
–	3	Aldehyde dehydrogenase	217, 219, 223, 224, 501	
–	3	Aldo-keto reductase	231, 10327	
–	3	Glycerol kinase	2710, 2712	
**Study 4**	**Cluster**	**Reaction**	**Gene Identifiers**	**Protein Identifiers**
–	1	Aminotransferase	189	
–	2	Aldehyde dehydrogenase	217, 219, 223, 224, 501	
–	2	Aldo-keto reductase	231, 10327	
–	2	Glycerol kinase	2710, 2712	
–	3	Purine nucleoside phosphorylase	4860	
–	3	Guanine deaminase	9615	
–	3	Xanthine dehydrogenase	7498	P47989
**Study 5**	**Cluster**	**Reaction**	**Gene Identifiers**	**Protein Identifiers**
–	1	Fumarate hydratase	2271	P07954
–	1	**Malic enzyme**	4199, 4200, 10873	P48163, P23368, Q16798
–	2	**Xanthine dehydrogenase**	7498	P47989
–	3	**Histidine decarboxylase**	3067	
–	3	Histamine N-methyltransferase	3176	

Reactions in clusters. Bold font denotes reactions of special interest in each cluster. Gene identifiers match records in Entrez Gene [6]. Protein identifiers match records in UniProt [7].

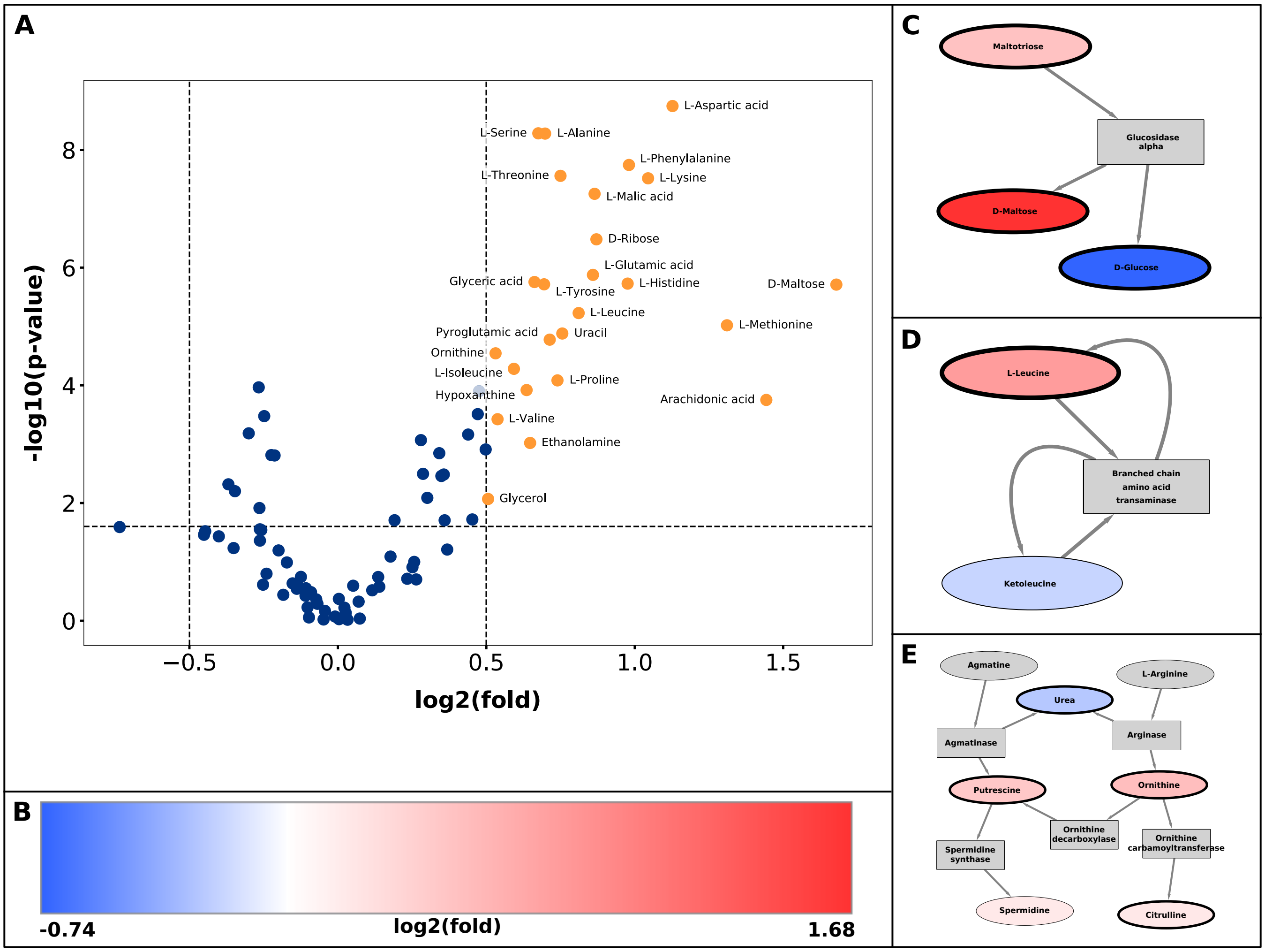

Figure 1. Integration and analysis of metabolomic measurements on metabolic networks, Study 2. Metabolomic Study 2 (**Table 3**) compared the abundances of 132 metabolites between visceral and subcutaneous adipose tissues. Clusters of enrichment in fold changes are detectable by integrating measurements within the non-compartmental network without hubs (**Figure 3D**). **A.** Volcano plot of p-values and fold changes in metabolites. **B.** Scale for color representation of fold changes on nodes in clusters. Extremes of color scale represent the minimal and maximal fold changes in the entire study. **C–E.** Clusters in metabolic network are detectable by enrichment of p-values and fold changes. Metabolite nodes in clusters represent fold changes by color fill, and they represent p-value by border thickness (p-value < 0.05).

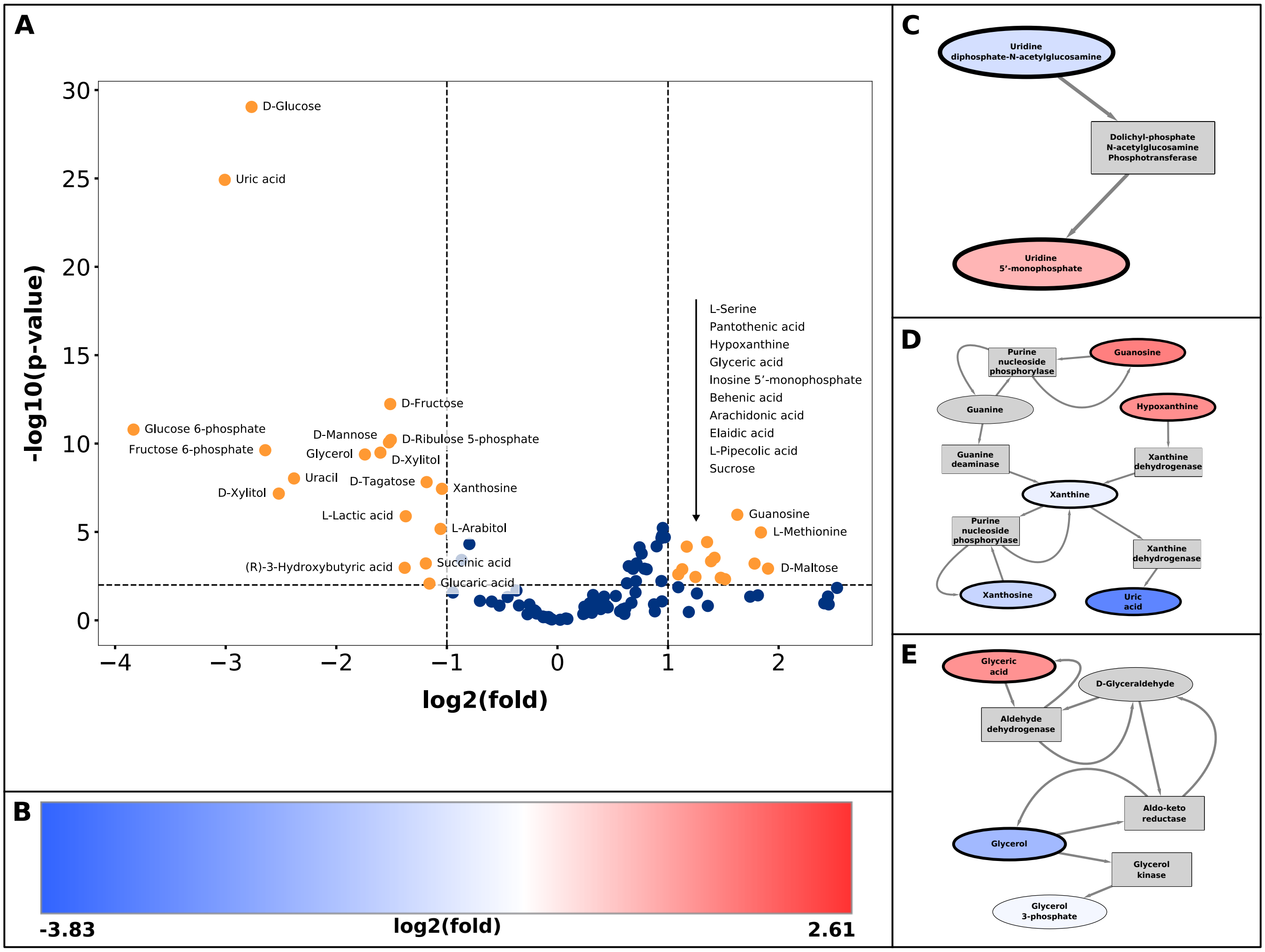

Figure 2. Integration and analysis of metabolomic measurements on metabolic networks, Study 3. Metabolomic Study 3 (**Table 3**) compared the abundances of 151 metabolites between ischemic and normal liver tissues. Clusters of enrichment in fold changes are detectable by integrating measurements within the non-compartmental network without hubs (**Figure 3D**). **A.** Volcano plot of p-values and fold changes in metabolites. **B.** Scale for color representation of fold changes on nodes in clusters. Extremes of color scale represent the minimal and maximal fold changes in the entire study. **C–E.** Clusters in metabolic network are detectable by enrichment of p-values and fold changes. Metabolite nodes in clusters represent fold changes by color fill, and they represent p-value by border thickness (p-value < 0.05).

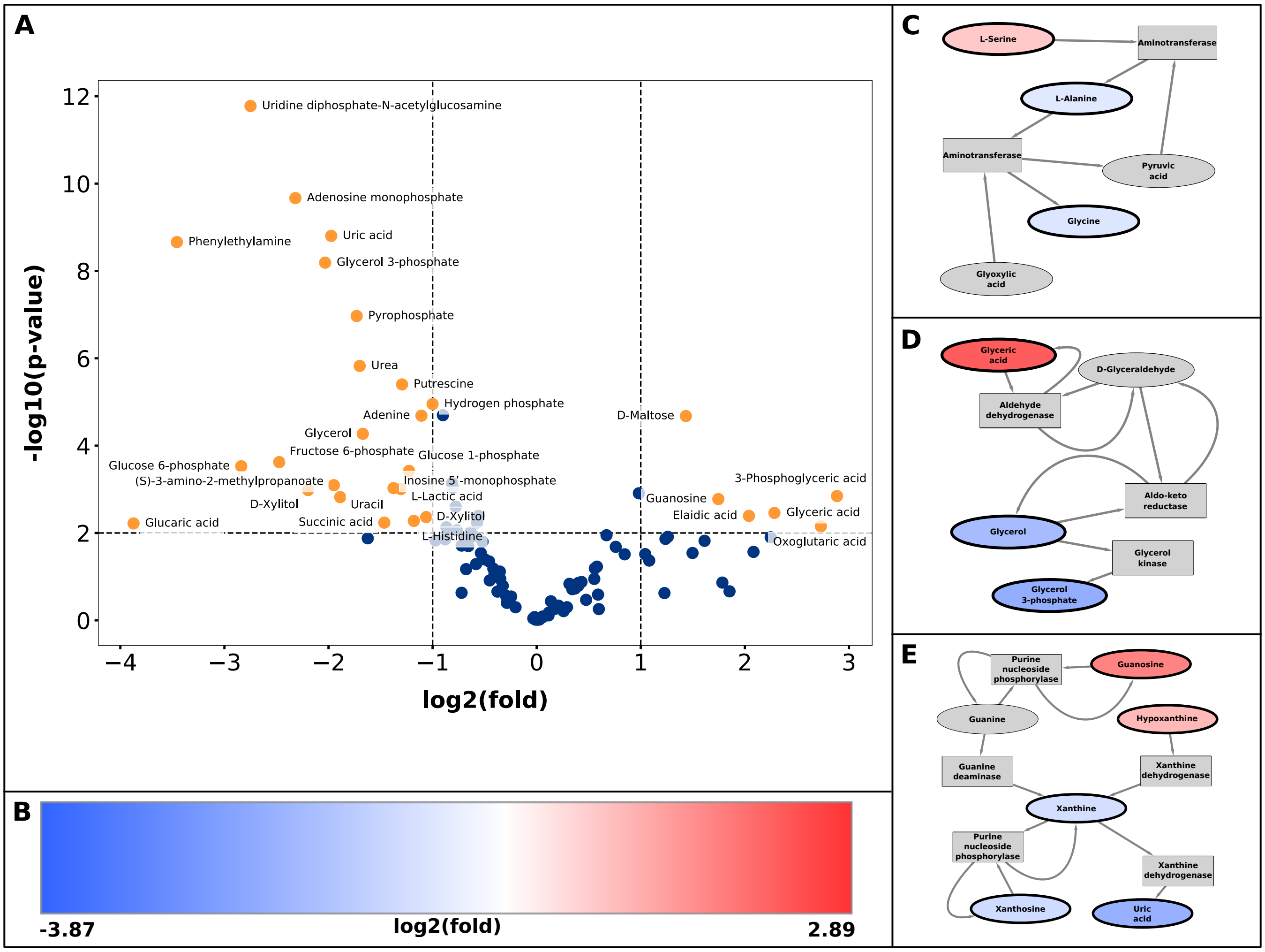

Figure 3. Integration and analysis of metabolomic measurements on metabolic networks, Study 4. Metabolomic Study 4 (**Table 3**) compared the abundances of 151 metabolites between steatotic and normal liver tissues. Clusters of enrichment in fold changes are detectable by integrating measurements within the non-compartmental network without hubs (**Figure 3D**). **A.** Volcano plot of p-values and fold changes in metabolites. **B.** Scale for color representation of fold changes on nodes in clusters. Extremes of color scale represent the minimal and maximal fold changes in the entire study. **C–E.** Clusters in metabolic network are detectable by enrichment of p-values and fold changes. Metabolite nodes in clusters represent fold changes by color fill, and they represent p-value by border thickness (p-value < 0.05).

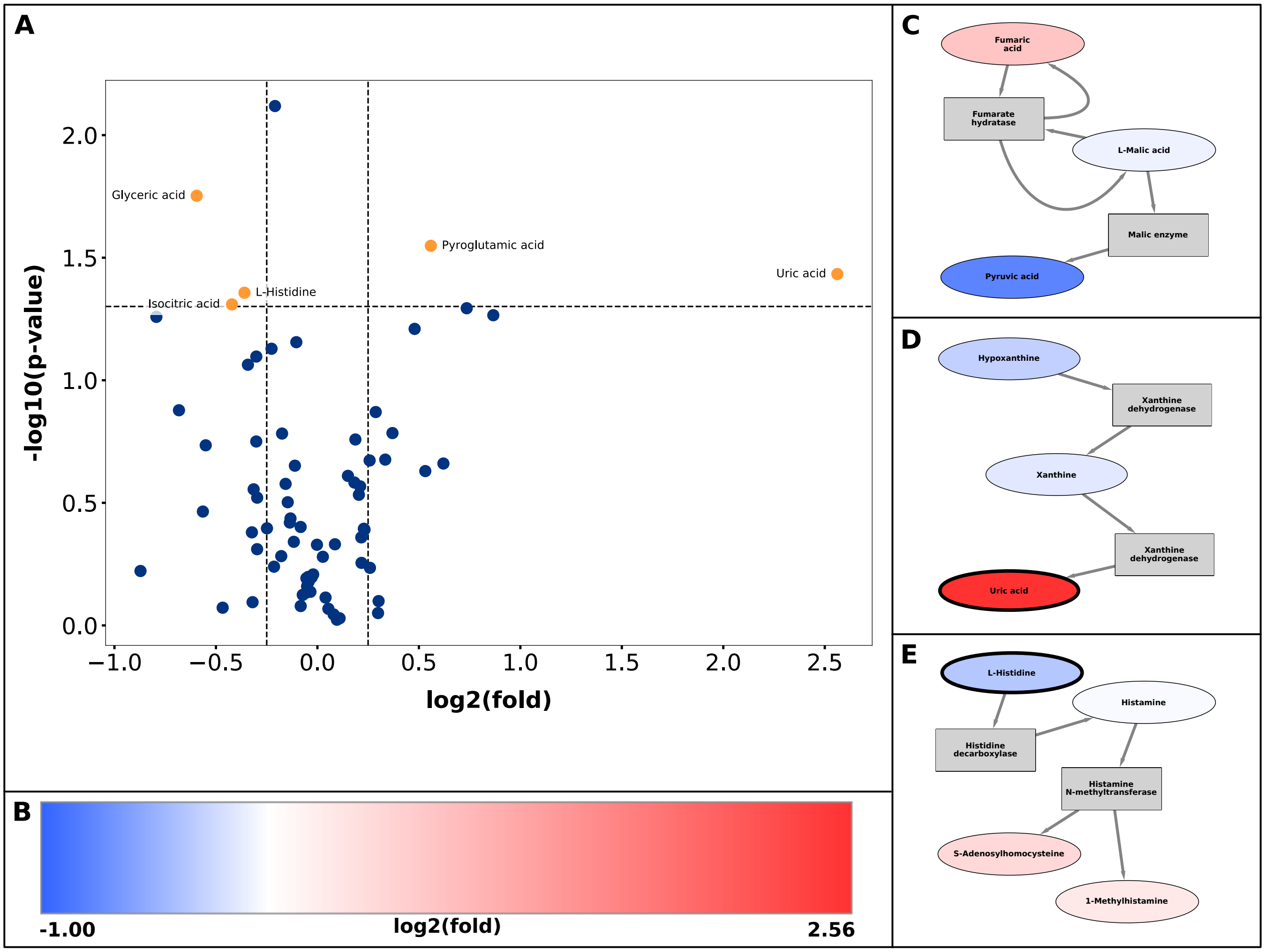

Figure 4. Integration and analysis of metabolomic measurements on metabolic networks, Study 5. Metabolomic Study 5 (**Table 3**) compared the abundances of 125 metabolites between muscle tissues before and after persons with obesity participated in a three-month exercise program. Clusters of enrichment in fold changes are detectable by integrating measurements within the non-compartmental network without hubs (**Figure 3D**). **A.** Volcano plot of p-values and fold changes in metabolites. **B.** Scale for color representation of fold changes on nodes in clusters. Extremes of color scale represent the minimal and maximal fold changes in the entire study. **C–E.** Clusters in metabolic network are detectable by enrichment of p-values and fold changes. Metabolite nodes in clusters represent fold changes by color fill, and they represent p-value by border thickness (p-value < 0.05).

References

1. Chong J, Soufan O, Li C, Caraus I, Li S, Bourque G, et al. MetaboAnalyst 4.0: towards more transparent and integrative metabolomics analysis. Nucleic Acids Research 2018 Jul;46(W1):W486–W494.
2. Shannon P, Markiel A, Ozier O, Baliga NS, Wang JT, Ramage D, et al. Cytoscape: a software environment for integrated models of biomolecular interaction networks. Genome Research 2003 Nov;13(11):2498–2504.
3. Ideker T, Ozier O, Schwikowski B, Siegel AF. Discovering regulatory and signalling circuits in molecular interaction networks. Bioinformatics (Oxford, England) 2002;18 Suppl 1:S233–240.
4. Saito R, Smoot ME, Ono K, Ruscheinski J, Wang PL, Lotia S, et al. A travel guide to Cytoscape plugins. Nature Methods 2012 Nov;9(11):1069–1076.
5. Mitra K, Carvunis AR, Ramesh SK, Ideker T. Integrative approaches for finding modular structure in biological networks. Nature Reviews Genetics 2013 Oct;14(10):719–732.
6. Brown GR, Hem V, Katz KS, Ovetsky M, Wallin C, Ermolaeva O, et al. Gene: a gene-centered information resource at NCBI. Nucleic Acids Research 2015 Jan;43(Database issue):D36–42.
7. UniProt Consortium T. UniProt: the universal protein knowledgebase. Nucleic Acids Research 2018 Mar;46(5):2699.
8. Pavlova NN, Thompson CB. The Emerging Hallmarks of Cancer Metabolism. Cell Metabolism 2016 Jan;23(1):27–47.
9. Vander Heiden MG, DeBerardinis RJ. Understanding the Intersections between Metabolism and Cancer Biology. Cell 2017;168(4):657–669.
10. Wikoff WR, Grapov D, Fahrmann JF, DeFelice B, Rom WN, Pass HI, et al. Metabolomic markers of altered nucleotide metabolism in early stage adenocarcinoma. Cancer Prevention Research (Philadelphia, Pa) 2015 May;8(5):410–418.
11. Hitosugi T, Zhou L, Elf S, Fan J, Kang HB, Seo JH, et al. Phosphoglycerate mutase 1 coordinates glycolysis and biosynthesis to promote tumor growth. Cancer Cell 2012 Nov;22(5):585–600.
12. Lin R, Elf S, Shan C, Kang HB, Ji Q, Zhou L, et al. 6-Phosphogluconate dehydrogenase links oxidative PPP, lipogenesis and tumour growth by inhibiting LKB1-AMPK signalling. Nature Cell Biology 2015 Nov;17(11):1484–1496.
13. Mattaini KR, Sullivan MR, Vander Heiden MG. The importance of serine metabolism in cancer. The Journal of Cell Biology 2016;214(3):249–257.
14. Oslund RC, Su X, Haugbro M, Kee JM, Esposito M, David Y, et al. Bisphosphoglycerate mutase controls serine pathway flux via 3-phosphoglycerate. Nature Chemical Biology 2017 Oct;13(10):1081–1087.
15. Pritlove DC, Gu M, Boyd CaR, Randeva HS, Vatish M. Novel placental expression of 2,3-bisphosphoglycerate mutase. Placenta 2006 Aug;27(8):924–927.
16. Salem A, Asselin MC, Reymen B, Jackson A, Lambin P, West CML, et al. Targeting Hypoxia to Improve Non-Small Cell Lung Cancer Outcome. Journal of the National Cancer Institute 2018 Jan;110(1).
17. Barrett T, Wilhite SE, Ledoux P, Evangelista C, Kim IF, Tomashevsky M, et al. NCBI GEO: archive for functional genomics data sets–update. Nucleic Acids Research 2013 Jan;41(Database issue):D991–995.
18. Wang L, Series GSE16515, Data Set GDS4102, Profile 78838150. Gene Expression Omnibus (GEO); 2009. https://www.ncbi.nlm.nih.gov/geoprofiles/78838150.
19. Pei H, Li L, Fridley BL, Jenkins GD, Kalari KR, Lingle W, et al. FKBP51 affects cancer cell response to chemotherapy by negatively regulating Akt. Cancer Cell 2009 Sep;16(3):259–266.
20. Reyes I, Series GSE3678, Data Set GDS1732, Profile 18862150. Gene Expression Omnibus (GEO); 2005. https://www.ncbi.nlm.nih.gov/geoprofiles/18862150.
21. Kummer NT, Nowicki TS, Azzi JP, Reyes I, Iacob C, Xie S, et al. Arachidonate 5 lipoxygenase expression in papillary thyroid carcinoma promotes invasion via MMP-9 induction. Journal of Cellular Biochemistry 2012 Jun;113(6):1998–2008.
22. Flöter J, Kaymak I, Schulze A. Regulation of Metabolic Activity by p53. Metabolites 2017 May;7(2).
23. Gerin I, Noël G, Bolsée J, Haumont O, Van Schaftingen E, Bommer GT. Identification of TP53-induced glycolysis and apoptosis regulator (TIGAR) as the phosphoglycolate-independent 2,3-bisphosphoglycerate phosphatase. The Biochemical Journal 2014 Mar;458(3):439–448.
24. Bolaños JP. TIGAR's promiscuity. The Biochemical Journal 2014 Mar;458(3):e5–7.

25. Casero RA, Murray Stewart T, Pegg AE. Polyamine metabolism and cancer: treatments, challenges and opportunities. Nature Reviews Cancer 2018 Nov;18(11):681–695.
26. Murray-Stewart TR, Woster PM, Casero RA. Targeting polyamine metabolism for cancer therapy and prevention. The Biochemical Journal 2016;473(19):2937–2953.
27. Garavito MF, Narváez-Ortiz HY, Zimmermann BH. Pyrimidine Metabolism: Dynamic and Versatile Pathways in Pathogens and Cellular Development. Journal of Genetics and Genomics = Yi Chuan Xue Bao 2015 May;42(5):195–205.
28. Luengo A, Gui DY, Vander Heiden MG. Targeting Metabolism for Cancer Therapy. Cell Chemical Biology 2017 Sep;24(9):1161–1180.
29. Lynes MD, Tseng YH. Deciphering adipose tissue heterogeneity. Annals of the New York Academy of Sciences 2018 Jan;1411(1):5–20.
30. Ibrahim MM. Subcutaneous and visceral adipose tissue: structural and functional differences. Obesity Reviews: An Official Journal of the International Association for the Study of Obesity 2010 Jan;11(1):11–18.
31. Xourgia E, Papazafiropoulou A, Melidonis A. Effects of antidiabetic drugs on epicardial fat. World Journal of Diabetes 2018 Sep;9(9):141–148.
32. Liesenfeld DB, Grapov D, Fahrmann JF, Salou M, Scherer D, Toth R, et al. Metabolomics and transcriptomics identify pathway differences between visceral and subcutaneous adipose tissue in colorectal cancer patients: the ColoCare study. The American Journal of Clinical Nutrition 2015 Aug;102(2):433–443.
33. Sugimoto S, Nakajima H, Kosaka K, Hosoi H. Review: Miglitol has potential as a therapeutic drug against obesity. Nutrition & Metabolism 2015;12:51.
34. Lynch CJ, Adams SH. Branched-chain amino acids in metabolic signalling and insulin resistance. Nature Reviews Endocrinology 2014 Dec;10(12):723–736.
35. Zhao X, Han Q, Liu Y, Sun C, Gang X, Wang G. The Relationship between Branched-Chain Amino Acid Related Metabolomic Signature and Insulin Resistance: A Systematic Review. Journal of Diabetes Research 2016;2016:2794591.
36. Arany Z, Neinast M. Branched Chain Amino Acids in Metabolic Disease. Current Diabetes Reports 2018 Aug;18(10):76.
37. Lee PL, Jung SM, Guertin DA. The Complex Roles of Mechanistic Target of Rapamycin in Adipocytes and Beyond. Trends in endocrinology and metabolism: TEM 2017;28(5):319–339.
38. Caldwell RW, Rodriguez PC, Toque HA, Narayanan SP, Caldwell RB. Arginase: A Multifaceted Enzyme Important in Health and Disease. Physiological Reviews 2018;98(2):641–665.
39. Zafar U, Khaliq S, Ahmad HU, Manzoor S, Lone KP. Metabolic syndrome: an update on diagnostic criteria, pathogenesis, and genetic links. Hormones (Athens, Greece) 2018 Sep;17(3):299–313.
40. Kachman M, Horowitz J, Project PR000599, Study ST000842. Metabolomics Workbench; 2017. https://doi.org/10.21228/M87Q3F.
41. Nielsen TT, Støttrup NB, Løfgren B, Bøtker HE. Metabolic fingerprint of ischaemic cardioprotection: importance of the malate-aspartate shuttle. Cardiovascular Research 2011 Aug;91(3):382–391.
42. Doenst T, Nguyen TD, Abel ED. Cardiac metabolism in heart failure: implications beyond ATP production. Circulation Research 2013 Aug;113(6):709–724.
43. Chicco AJ, Le CH, Gnaiger E, Dreyer HC, Muyskens JB, D'Alessandro A, et al. Adaptive remodeling of skeletal muscle energy metabolism in high-altitude hypoxia: Lessons from AltitudeOmics. The Journal of Biological Chemistry 2018 May;293(18):6659–6671.
44. Yang Z, Lanks CW, Tong L. Molecular mechanism for the regulation of human mitochondrial NAD(P)+-dependent malic enzyme by ATP and fumarate. Structure (London, England: 1993) 2002 Jul;10(7):951–960.
45. Kang DH, Ha SK. Uric Acid Puzzle: Dual Role as Antioxidantand Pro-oxidant. Electrolyte & blood pressure: E & BP 2014 Jun;12(1):1–6.
46. Ferraro E, Giammarioli AM, Chiandotto S, Spoletini I, Rosano G. Exercise-induced skeletal muscle remodeling and metabolic adaptation: redox signaling and role of autophagy. Antioxidants & Redox Signaling 2014 Jul;21(1):154–176.
47. Trewin AJ, Berry BJ, Wojtovich AP. Exercise and Mitochondrial Dynamics: Keeping in Shape with ROS and AMPK. Antioxidants (Basel, Switzerland) 2018 Jan;7(1).
48. Luttrell MJ, Halliwill JR. The Intriguing Role of Histamine in Exercise Responses. Exercise and Sport Sciences Reviews 2017;45(1):16–23.
49. Liao H, Mariani MS, Medo M, Zhang YC, Zhou MY. Ranking in evolving complex networks. Physics Reports 2017 May;689:1–54. https://linkinghub.elsevier.com/retrieve/pii/S0370157317300935.
50. Humphries MD, Gurney K. Network 'small-world-ness': a quantitative method for determining canonical network equivalence. PloS One 2008 Apr;3(4):e0002051.
51. Pavlopoulos GA, Kontou PI, Pavlopoulou A, Bouyioukos C, Markou E, Bagos PG. Bipartite graphs in systems biology and medicine: a survey of methods and applications. GigaScience 2018;7(4):1–31.
52. Fiehn O, Project PR000305, Study ST000390. Metabolomics Workbench; 2010. https://doi.org/10.21228/M8PG66.
53. Fiehn O, Project PR000058, Study ST000061. Metabolomics Workbench; 2014. https://doi.org/10.21228/M80018.
54. Bruinsma BG, Sridharan GV, Weeder PD, Avruch JH, Saeidi N, Özer S, et al. Metabolic profiling during ex vivo machine perfusion of the human liver. Scientific Reports 2016 Mar;6:22415.
55. Fiehn O, Project PR000322, Study ST000412. Metabolomics Workbench; 2016. https://doi.org/10.21228/M8V312.
56. Sud M, Fahy E, Cotter D, Azam K, Vadivelu I, Burant C, et al. Metabolomics Workbench: An international repository for metabolomics data and metadata, metabolite standards, protocols, tutorials and training, and analysis tools. Nucleic Acids Research 2016 Jan;44(D1):D463–470.
