# Supplementary material for: Compartment and hub definitions tune metabolic networks for metabolomic interpretations
Source: Gigascience. 2020 Jan 23;9(1):giz137. doi: 10.1093/gigascience/giz137 (PMC6977586; doi:10.1093/gigascience/giz137)
Supplement: giz137_Response_to_Reviewer_Comments_Original_Submission [file giz137_response_to_reviewer_comments_original_submission.pdf]

Author's Response To Reviewer Comments

Close

Please refer to file "waller_response.pdf" for letter response to reviewer comments.

Close
