# Supplementary material for: Compartment and hub definitions tune metabolic networks for metabolomic interpretations
Source: Gigascience. 2020 Jan 23;9(1):giz137. doi: 10.1093/gigascience/giz137 (PMC6977586; doi:10.1093/gigascience/giz137)
Supplement: giz137_Reviewer_2_Report_Original_Submission — Shen Tong -- 1/8/2019 Reviewed [file giz137_reviewer_2_report_original_submission.pdf]

Reviewer Report

Title: Compartment and Hub Definitions Tune Metabolic Networks for Metabolomic Interpretations

Version: Original Submission **Date:** 1/8/2019

Reviewer name: Shen Tong

Reviewer Comments to Author:

The reviewer strongly encourages the development and debate for metabolomics data visualization to aid interpretation, as the current resources are simply scarce. The manuscript is technically sound, however, the written presentation needs to be drastically improved. There are redundant descriptions throughout the manuscript which hinder the readers' comprehension. The reviewer regrets to see the redundancy blurs the focus of the paper.

The authors ought to present their work concisely and precisely. The reviewer suggests to simply the structure of the manuscript to enhance clarity, shorten non-essential content e.g. Metabolomics measurement, Table 2, numbers of test studies to present. Eventually, the reviewer suggests the authors to have a peer scholar outside the disciplinary to proofread and to be able to comprehend prior to re-submission.

The structure of the paper could primarily reflect:

* Define compartment and hub in metabolic network visualization.In DyMetaboNet, what are the main functions to modulate compartment and hub [as key background]
* Comparison of compartmental vs non-compartmental, with hubs vs without hubs (like figure 2, 3 and 4) [as why]
* How does DyMetaboNet outperform other existing visualization tools? Quantitatively? [as why]
* Additional technical functionalities to use DyMetaboNet, such as centralization as in table 3. [as how]

The above are the most important messages to reviewers and readers, therefore should be written with high priority and clarity.

Despite of that,

1) Hubs appear to exert a dominant influence on the data visualization. The reviewer is convinced that excluding "hub" metabolites adds the benefit to enlighten metabolites' relationships in a complex network.

2) Non-compartmental network seems to have smaller impact on simplifying metabolites' relationship compared to hubs. As so, the reviewer is not totally convinced to remove compartmental information merely on the argument that metabolomics measurement is not compartment-specific. Therefore, to leave this function optional to users might be a stronger argument.

2b) Additionally, it is because the measurement is not compartment-specific that providing biochemical compartmental information for specific metabolites could be beneficial. The authors could consider preserving the metabolites that are strongly shown to be compartment-specific from database mining (e.g. cardiolipins in mitochondria).

3) The reviewer is happy to see more computational tools being developed to aid data interpretation.

Could I ask how would the authors plan to maintain and expand DyMetaboNet as entries of metabolites and reactions expand in queried databases?
4) How reproducible the DyMetaboNet outcome is, using the same dataset by different users?

Methods

Are the methods appropriate to the aims of the study, are they well described, and are necessary controls included? Choose an item.

Conclusions

Are the conclusions adequately supported by the data shown? Choose an item.

Reporting Standards

Does the manuscript adhere to the journal's guidelines on minimum standards of reporting? Choose an item.

Choose an item.

Statistics

Are you able to assess all statistics in the manuscript, including the appropriateness of statistical tests used? Choose an item.

Quality of Written English

Please indicate the quality of language in the manuscript: Choose an item.

Declaration of Competing Interests

Please complete a declaration of competing interests, considering the following questions:

- Have you in the past five years received reimbursements, fees, funding, or salary from an organisation that may in any way gain or lose financially from the publication of this manuscript, either now or in the future?
- Do you hold any stocks or shares in an organisation that may in any way gain or lose financially from the publication of this manuscript, either now or in the future?
- Do you hold or are you currently applying for any patents relating to the content of the manuscript?
- Have you received reimbursements, fees, funding, or salary from an organization that holds or has applied for patents relating to the content of the manuscript?
- Do you have any other financial competing interests?
- Do you have any non-financial competing interests in relation to this paper?

If you can answer no to all of the above, write 'I declare that I have no competing interests' below. If your reply is yes to any, please give details below.

I declare that I have no competing interests

I agree to the open peer review policy of the journal. I understand that my name will be included on my report to the authors and, if the manuscript is accepted for publication, my named report including any attachments I upload will be posted on the website along with the authors' responses. I agree for my report to be made available under an Open Access Creative Commons CC-BY license (http://creativecommons.org/licenses/by/4.0/). I understand that any comments which I do not wish to be included in my named report can be included as confidential comments to the editors, which will not be published.

Choose an item.

To further support our reviewers, we have joined with Publons, where you can gain additional credit to further highlight your hard work (see: https://publons.com/journal/530/gigascience). On publication of this paper, your review will be automatically added to Publons, you can then choose whether or not to claim your Publons credit. I understand this statement.

Yes Choose an item.
